# βIII-tubulin can act as a brake on extrinsic apoptosis in pancreatic cancer

**DOI:** 10.1038/s41419-026-08657-6

**Published:** 2026-04-24

**Authors:** George Sharbeen, John Kokkinos, Grace Schulstad, Elvis Pandzic, Janet Youkhana, Zerong Ma, Rosa Mistica C. Ignacio, Aparna S. Raina, Shannon Chiang, Cyrille Boyer, Koroush S. Haghighi, Matthew Gunawarman, David Goldstein, Val Gebski, Marina Pajic, Meagan E. Davis, Oliver S. M. Arkell, Chantal Kopecky, Estrella Gonzales-Aloy, Alexander Ishak, Mert Erkan, Jennifer P. Morton, Maria Kavallaris, Peter W. Gunning, Edna C. Hardeman, Amber Johns, Anthony J. Gill, Renee M. Whan, Amanda Mawson, Omali Pitiyarachchi, Australian Pancreatic Cancer Genome Initiative, Marina Pajic, Amber Johns, Anthony J. Gill, Joshua A. McCarroll, Phoebe A. Phillips

**Affiliations:** 1 Pancreatic Cancer Translational Research Group, School of Biomedical Sciences, Lowy Cancer Research Centre, UNSW Sydney, Sydney, NSW Australia; 2 Australian Centre for NanoMedicine, UNSW Sydney, Sydney, NSW Australia; 3 School of Medicine, Sydney Campus, University of Notre Dame Australia, Sydney, NSW Australia; 4 Katharina Gaus Light Microscopy Facility, Mark Wainwright Analytical Centre, Lowy Cancer Research Centre, UNSW Sydney, Sydney, NSW Australia; 5 Children’s Cancer Institute, Health Translation Hub, UNSW Sydney, Sydney, NSW Australia; 6 School of Clinical Medicine, UNSW Medicine and Health, UNSW Sydney, Sydney, NSW Australia; 7 Cluster for Advanced Macromolecular Design, School of Chemical Engineering, UNSW Sydney, Sydney, NSW Australia; 8 Prince of Wales Hospital, School of Clinical Medicine, Randwick Clinical Campus, UNSW Sydney, Sydney, NSW Australia; 9 NHMRC Clinical Trials Centre, The University of Sydney, Sydney, NSW Australia; 10 The Kinghorn Cancer Centre, Garvan Institute of Medical Research, Sydney, NSW Australia; 11 School of Clinical Medicine, St Vincent’s Healthcare Campus, UNSW Sydney, Sydney, NSW Australia; 12 School of Chemistry, UNSW Sydney, Sydney, NSW Australia; 13 Mehmet Ali Aydinlar Acibadem University, Atasehir Hospital, Istanbul, Turkey; 14 School of Cancer Sciences, University of Glasgow, Glasgow, UK; 15 Cancer Research UK Scotland Institute, Glasgow, UK; 16 UNSW RNA Institute, UNSW Sydney, Sydney, NSW Australia; 17 School of Biomedical Sciences, Faculty of Medicine and Health, UNSW Sydney, Sydney, NSW Australia; 18 Australian Pancreatic Cancer Genome Initiative (APGI), Garvan Institute of Medical Research, Sydney, NSW Australia; 19 Cancer Diagnosis and Pathology Group, Kolling Institute of Medical Research, Royal North Shore Hospital, Sydney, NSW Australia; 20 University of Sydney, Sydney, NSW Australia; 21 Garvan Institute of Medical Research, Sydney, NSW Australia

**Keywords:** Pancreatic cancer, Targeted therapies

## Abstract

The microtubule protein βIII-tubulin is a prognostic, pro-survival, and chemoresistance factor in multiple malignancies, including pancreatic ductal adenocarcinoma (PDAC). However, the precise survival mechanisms controlled by βIII-tubulin in cancer remain unknown. Here, we discovered a link between βIII-tubulin and the activation of caspase 8-mediated extrinsic apoptosis. Silencing βIII-tubulin in PDAC cells activated caspase 8, leading to decreased cell viability and growth both in vitro and in vivo. βIII-tubulin knockdown also increased the sensitivity of PDAC cells to extrinsic cell death signals, including TNF-related apoptosis-inducing ligand (TRAIL), TNFα, and FasL. Furthermore, we demonstrated that βIII-tubulin knockdown in PDAC cells, in the absence or presence of TRAIL, increased diffusion and clustering of the TRAIL death receptor DR5 at the cell membrane, inducing extrinsic apoptosis. Nanoparticle delivery of βIII-tubulin siRNA to mouse PDAC tumours reduced tumour growth and increased responsiveness to TRAIL therapy. In patient-derived human PDAC explants, βIII-tubulin silencing reduced tumour cell frequency and improved sensitivity to TRAIL. Finally, we showed that high βIII-tubulin expression in the human PDAC stroma was independently prognostic for poor overall survival. Taken together, silencing βIII-tubulin represents an innovative strategy to activate a suicide signal in PDAC cells and render them more sensitive to microenvironment- and chemotherapy-derived death signals.

## Introduction

Pancreatic ductal adenocarcinoma (PDAC) is one of the deadliest malignancies, with a 5-year survival of <11% [1]. This is attributed to diagnosis at late stage, with chemoresistance, metastases, and a complex multicellular microenvironment that drives tumour progression [2, 3]. The highly fibrotic stroma produced by cancer-associated fibroblasts (CAFs) can hinder drug delivery and, together with pro-tumourigenic crosstalk between PDAC cells and CAFs, promotes metastases and chemoresistance [2–4]. Therapeutic strategies should not simply target PDAC cells alone, but address the complex and heterogeneous multicellular microenvironment that characterises PDAC [2–4].

The microtubule protein, βIII-tubulin, is upregulated in solid tumours and correlates with poor patient outcome [5–11]. In PDAC, βIII-tubulin is upregulated in patient tumour specimens [12–14]. Several studies in multiple cancers have reported links between βIII-tubulin and pro-tumour signalling pathways [15], protection against nutrient deprivation and microenvironment-derived stress [16], and promotion of chemoresistance [12, 17–20] and metastasis [20, 21]. However, the mechanism by which βIII-tubulin regulates these survival pathways and protects cancer cells from chemotherapy and microenvironment-derived stress remains unknown.

Apoptosis in cancer cells is activated through the intrinsic and extrinsic pathways, both of which have been extensively studied and characterised [22]. Briefly, the intrinsic pathway is triggered by the permeabilisation of the mitochondrial outer membrane, leading to the release of cytochrome C into the cytosol, activation of initiator caspase 9, and subsequent activation of executioner caspases 3 and 7. Extrinsic apoptosis is initiated by clustering of death receptors (TNFR1, Fas, TRAIL-R1/DR4, TRAIL-R2/DR5) upon binding with ligands such as TRAIL, TNFα, and FasL. Upon activation, these receptors serve as docking sites for several key intracellular proteins, including caspase 8, that form a death-inducing signalling complex (DISC), which in turn activates caspases 3 and 7, leading to apoptosis. Previously, we demonstrated that knockdown of βIII-tubulin expression in PDAC cells led to apoptosis [12]. However, the roles of the intrinsic and extrinsic pathways in regulating βIII-tubulin’s effect on PDAC apoptosis remain unknown. Here, we discovered that βIII-tubulin confines TRAIL Death Receptor 5 (DR5) at the cell membrane and limits its activation of caspase 8-dependent extrinsic apoptosis in PDAC cells. Silencing βIII-tubulin expression allowed DR5 to cluster at the cell membrane, activate extrinsic apoptosis, and increase sensitivity to extrinsic apoptosis inducers (TNF-α, FasL, TRAIL).

We used polymeric nanoparticles [23] to deliver βIII-tubulin siRNA in PDAC mouse models and in patient-derived PDAC explants ex vivo [24]. In orthotopic PDAC mouse tumours, βIII-tubulin siRNA reduced tumour growth and increased intracellular cleaved caspase-8 positive cell numbers. In a subcutaneous mouse PDAC model, silencing βIII-tubulin also reduced tumour growth and increased the number of responders to TRAIL therapy. Additionally, in human patient-derived PDAC explants, βIII-tubulin silencing decreased tumour cell frequency and increased TRAIL sensitivity. Finally, using a PDAC patient cohort, we demonstrated that high βIII-tubulin in tumour cells correlates with poor overall survival and that high stromal βIII-tubulin was independently prognostic of poorer overall patient survival.

## Results

### Knockdown of βIII-tubulin in PDAC cells activated extrinsic apoptosis in vitro and in vivo

**Fig. 1:**
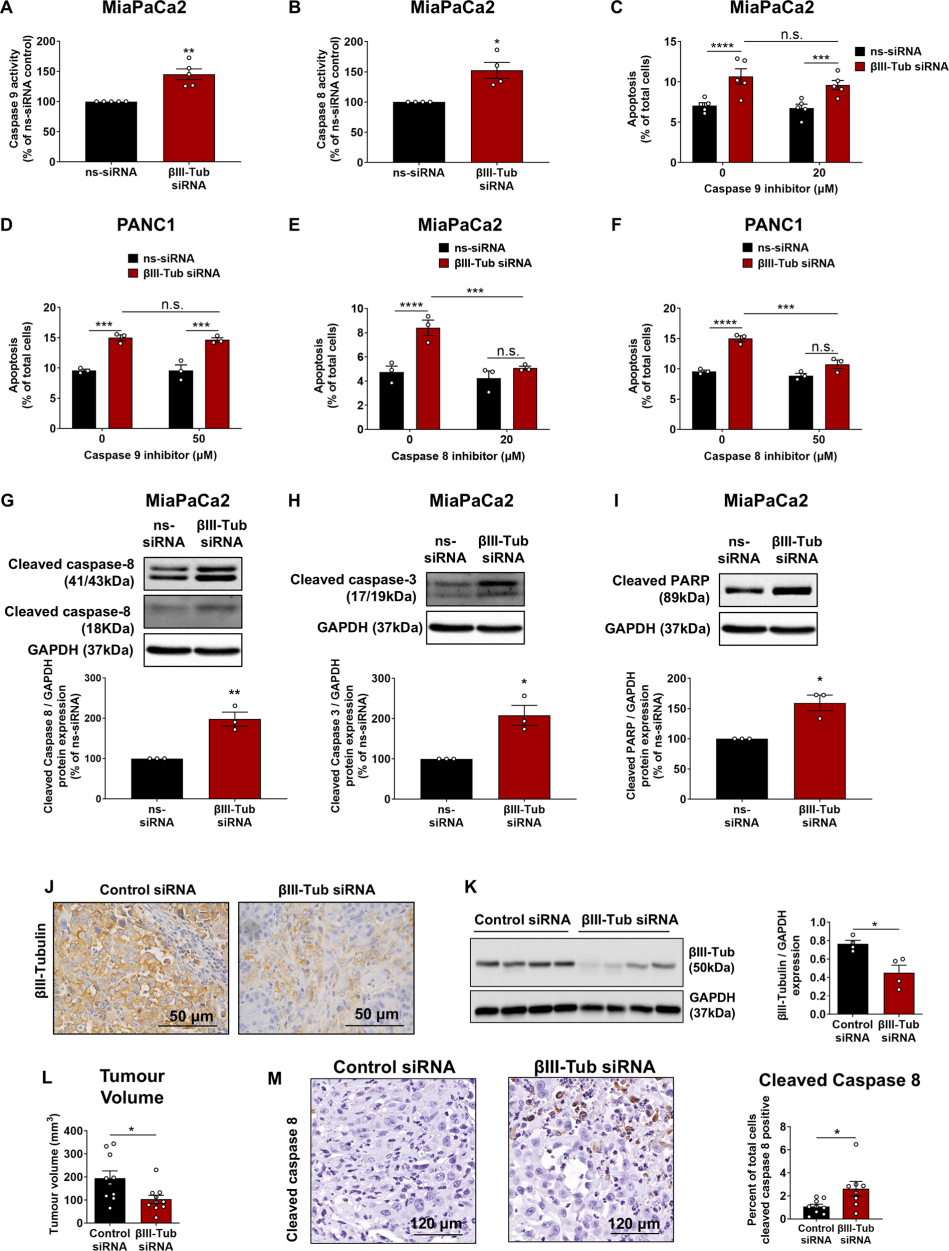
Knockdown of βIII-tubulin in PDAC cells activated the extrinsic pathway of apoptosis in vitro and in vivo. Caspase 9 (*n* = 5) **A** and caspase 8 (*n* = 4) **B** activities in MiaPaCa2 cells measured using CaspaseGlo assays, 72 h post-transfection with non-silencing (ns) or βIII-tubulin (βIII-Tub) siRNA. Activity normalised to cell numbers. **C–F** Apoptosis measured using flow cytometry for Annexin V/DAPI in PDAC cells, 72 hours post-transfection with βIII-tubulin siRNA, and 24 h post-treatment with caspase 9 inhibitor (Z-LEHD-FMK) **C, D** or caspase 8 inhibitor (Q-IETD-OPh) (**E**, **F**). Results from panels D and F were obtained using the same controls for 0 μM caspase inhibitor. Representative western blot and densitometry analysis showing cleaved caspase 8 (**G**), cleaved caspase 3 (**H**) and cleaved PARP (**I**) protein expression for MiaPaCa2 cells transfected with ns- or βIII-tubulin siRNA for 72 h. Bars represent mean of *n* ≥ 3 independent experiments (data points shown from independent experiments) ± SEM. Asterisks indicate significance as assessed by two-tailed paired *t*-tests or one-way ANOVA (*p ≤ 0.05, **p ≤ 0.01, ***p ≤ 0.001, ****p ≤ 0.0001; n.s: non-significant). **J** Representative immunohistochemistry for βIII-tubulin (βIII-Tub) in sections from tumours treated with Star 3+control siRNA or βIII-tubulin siRNA. **K** Western blot of protein extracts from 8 mouse tumours treated with Star 3+control siRNA (*n* = 4) or βIII-tubulin siRNA (*n* = 4) and densitometry analysis. GAPDH was used as loading control. **L** Tumour volume at endpoint. **M** Cleaved caspase 8 immunohistochemistry representative images and quantification in Star 3+control siRNA (*n* = 9) and Star 3 + βIII-tubulin siRNA (*n* = 8). One tumour was excluded from controls, and 2 tumours excluded from βIII-tubulin siRNA group due to insufficient tumour tissue. Bars represent mean of *n* ≥ 4 (data points indicate individual mice) ± SEM. Asterisks indicate significance by two-tailed unpaired *t*-tests (*p ≤ 0.05; n.s.: non-significant).

First, we validated that a pool of four individual βIII-tubulin siRNAs (Smartpool) and a single siRNA as well as shRNA designed to stably knockdown βIII-tubulin significantly silenced βIII-tubulin expression in human PDAC cells (Supplementary Fig. 1). Knocking down βIII-tubulin using siRNA in PDAC (MiaPaCa2) cells increased caspase-9 and caspase-8 activity compared to controls (Fig. 1A, B). However, when we used caspase-9 or caspase-8 inhibitors, only caspase-8 inhibition prevented cell death in βIII-tubulin knockdown PDAC cells (Fig. 1C–F). Western blotting confirmed that βIII-tubulin knockdown in PDAC cells induced cleavage of caspase 8, caspase-3 and Poly (ADP-ribose) polymerase (PARP) (apoptosis markers; Fig. 1G–I). Likewise, treatment of orthotopic PDAC mouse tumours with βIII-tubulin siRNA complexed to polymeric nanoparticles (Star 3) induced βIII-tubulin knockdown (Fig. 1J, K), significantly decreased pancreatic tumour volume (Fig. 1L) and increased cleaved caspase-8 positive cells (Fig. 1M).

### βIII-tubulin knockdown in PDAC cells enhanced cell death in the presence of tumour necrosis factor (TNF)-related apoptosis-inducing ligand (TRAIL)

**Fig. 2:**
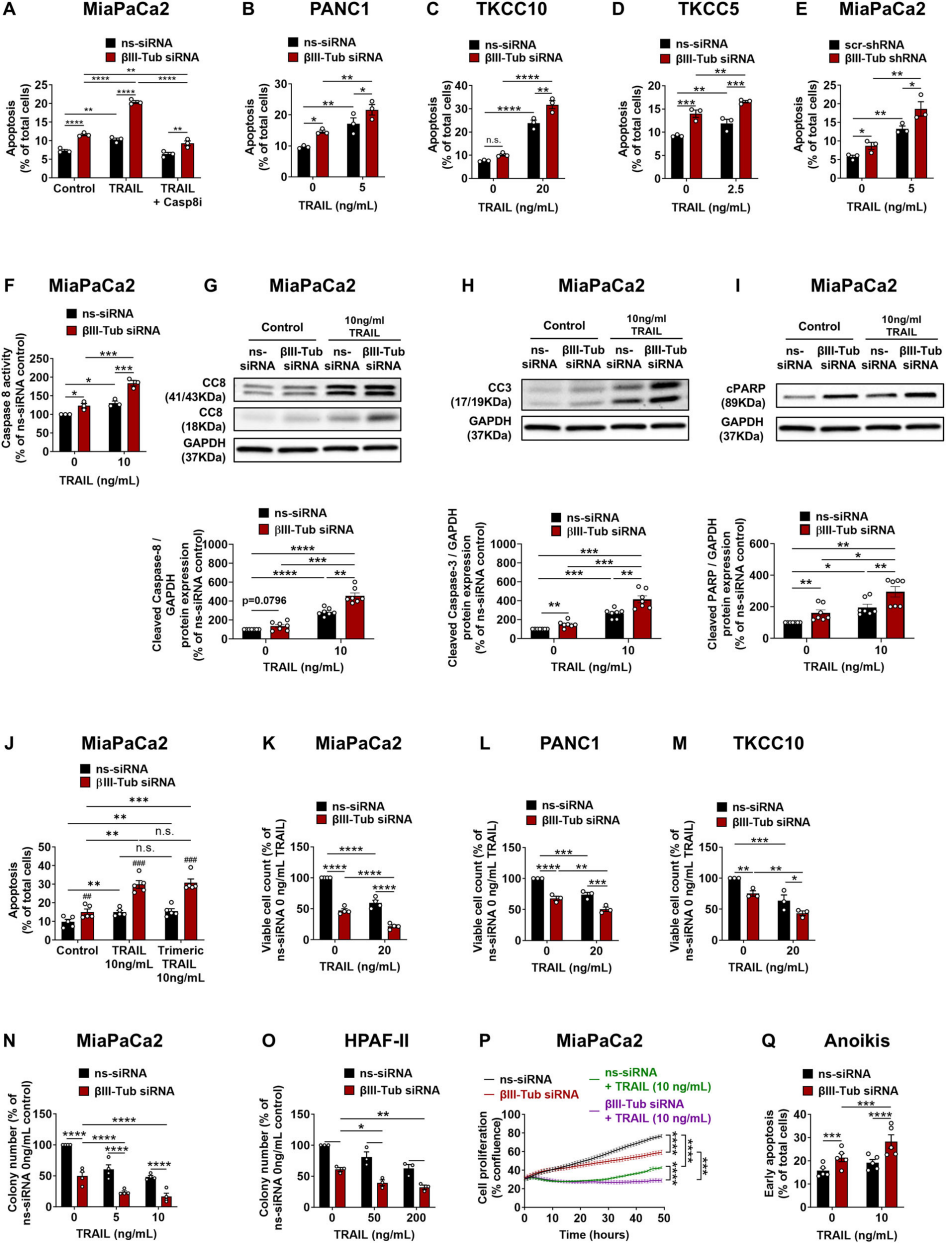
Knockdown of βIII-tubulin in PDAC cells increased sensitivity to TRAIL-induced extrinsic apoptosis. Apoptosis measured using flow cytometry for Annexin V/DAPI in PDAC cells transfected with non-silencing (ns) or βIII-tubulin (βIII-Tub) siRNA and treated ± TRAIL in MiaPaCa2 (*n* = 5) (**A**), PANC1 (n = 3) (**B**), TKCC10 (*n* = 3) (**C**), and TKCC5 (**D**) cells. MiaPaCa2 cells were pre-treated ± caspase 8 inhibitor (20 μM) for 1 h prior to addition of TRAIL (10 ng/mL) (**A**). **E** Apoptosis measured in MiaPaCa2 cells (*n* = 3) ± TRAIL with stable expression of βIII-tubulin or scramble (scr) shRNA. **F** Caspase 8 activity measured in MiaPaCa2 cells (*n* = 3) with βIII-tubulin knockdown ± TRAIL. Caspase 8 activity was normalised to cell numbers with CCK-8 assay. Confirmation of induction of apoptosis by βIII-tubulin knockdown ± TRAIL in MiaPaCa-2 cells, by Western blot for cleaved caspase 8 (CC8) (**G**), cleaved caspase 3 (CC3) (**H**), and cleaved PARP (cPARP) (**I**). **J** Apoptosis measured using flow cytometry for Annexin V/DAPI in MiaPaCa2 cells transfected with ns-siRNA or βIII-Tub-siRNA and treated ± TRAIL or ± trimeric TRAIL (*n* = 4). Viable cell count (trypan blue exclusion assay) of MiaPaCa2 (*n* = 4) (**K**), PANC1 (*n* = 3) (**L**), and TKCC10 (*n* = 3) (**M**) PDAC cells transfected with ns-siRNA or βIII-Tub siRNA and treated ± TRAIL. MiaPaCa2 (*n* = 4) (**N**) and HPAF-II (*n* = 3) (**O**) colonies formed from low seeding density, post-transfection with ns-siRNA or βIII-tubulin siRNA ± TRAIL. **P** IncuCyte® S3 live-cell analysis (proliferation) of MiaPaCa2 cells (*n* = 4) with βIII-tubulin knockdown ± TRAIL. **Q** MiaPaCa2 cells (*n* = 5) transfected with ns-siRNA or βIII-tubulin siRNA were cultured under anchorage independent conditions for 24 h, then treated with TRAIL for 9 h. Early apoptosis (Annexin V+/DAPI−) was measured using flow cytometry. Bars represent mean of *n* ≥ 3 independent experiments (data points shown from independent experiments) ± SEM. Asterisks indicate significance as assessed by one-way ANOVA (*p ≤ 0.05, **p ≤ 0.01, ***p ≤ 0.001, ****p ≤ 0.0001; n.s.: non-significant).

Combining βIII-tubulin smartpool siRNA with recombinant TRAIL in PDAC cells, shown to express both TRAIL receptors DR4 and DR5 (Supplementary Fig. 2A) increased apoptosis more than each single treatment (Fig. 2A, D). Comparable results were observed in MiaPaCa2 cells stably expressing βIII-tubulin shRNA (Fig. 2E) or treated with a single-sequence βIII-tubulin siRNA (Supplementary Fig. 2B). Combination treatment increased caspase-8 activity, and cleaved caspase-8/3/PARP compared to single treatments (Fig. 2F–I). Apoptosis induced by βIII-tubulin knockdown + TRAIL was inhibited by caspase-8 blockade (Fig. 2A). To determine if effects were driven by the oligomerisation state of native recombinant TRAIL used above (can exist in monomeric and reversible oligomeric forms), we repeated the experiments using TRAIL forced into a trimeric state through a fused isoleucine zipper. We observed that trimeric TRAIL and recombinant TRAIL were equally effective at inducing apoptosis and in enhancing βIII-tubulin knockdown-induced apoptosis in PDAC cells (Fig. 2J and Supplementary Fig. 2C–F). Increased apoptosis was associated with a significant reduction in cell viability and clonogenic growth (Fig. 2K–O). To understand if this was a conserved mechanism across other βIII-tubulin overexpressing tumours, we performed the same tests in non-small cell lung cancer cells. βIII-tubulin knockdown induced a similar induction of extrinsic apoptosis and sensitisation to TRAIL (Supplementary Fig. 3). While the majority of apoptosis and cell viability effects observed above were additive, it should be noted that these assays were static time points and that the cumulative effects are demonstrated by the complete ablation of cell proliferation in MiaPaCa2 and H460 cells (Fig. 2P and Supplementary Fig. 3G). Knockdown of βIII-tubulin + TRAIL also induced anchorage-independent cell death (anoikis) compared to single treatments (Fig. 2Q).

We examined the effects of βIII-tubulin knockdown with TRAIL in non-neoplastic pancreatic CAFs, which expressed similar levels of βIII-tubulin (Supplementary Figs. 1J and 4A). However, in contrast to PDAC cells, silencing βIII-tubulin in CAFs in the absence of TRAIL had no effect on apoptosis (Supplementary Fig. 4B). TRAIL treatment alone induced a downward trend in apoptosis in CAFs, but there was no difference in apoptosis between CAFs with βIII-tubulin knockdown alone and the combination treatment (Supplementary Fig. 4B). The statistically significant differences observed between ns-siRNA and βIII-tubulin-siRNA treated CAFs in the presence of TRAIL (Supplementary Fig. 4B) were likely due to TRAIL-induced downward trend in apoptosis, rather than sensitisation. CAF cell proliferation was reduced with βIII-tubulin knockdown but showed no further reduction with TRAIL (Supplementary Fig. 4C). There was no link between expression of TRAIL receptors and TRAIL sensitivity in CAFs, as relative to PDAC cells, patient-derived CAFs showed lower levels of DR4 (Supplementary Fig. 4D) and comparable levels of DR5 (Supplementary Fig. 4D). Finally, we observed that βIII-tubulin knockdown in CAFs increased cell senescence compared to controls (Supplementary Fig. 4E).

### Silencing of βIII-tubulin in PDAC cells increased sensitivity to tumour microenvironment-derived extrinsic apoptosis inducers - TNFα and FasL

**Fig. 3:**
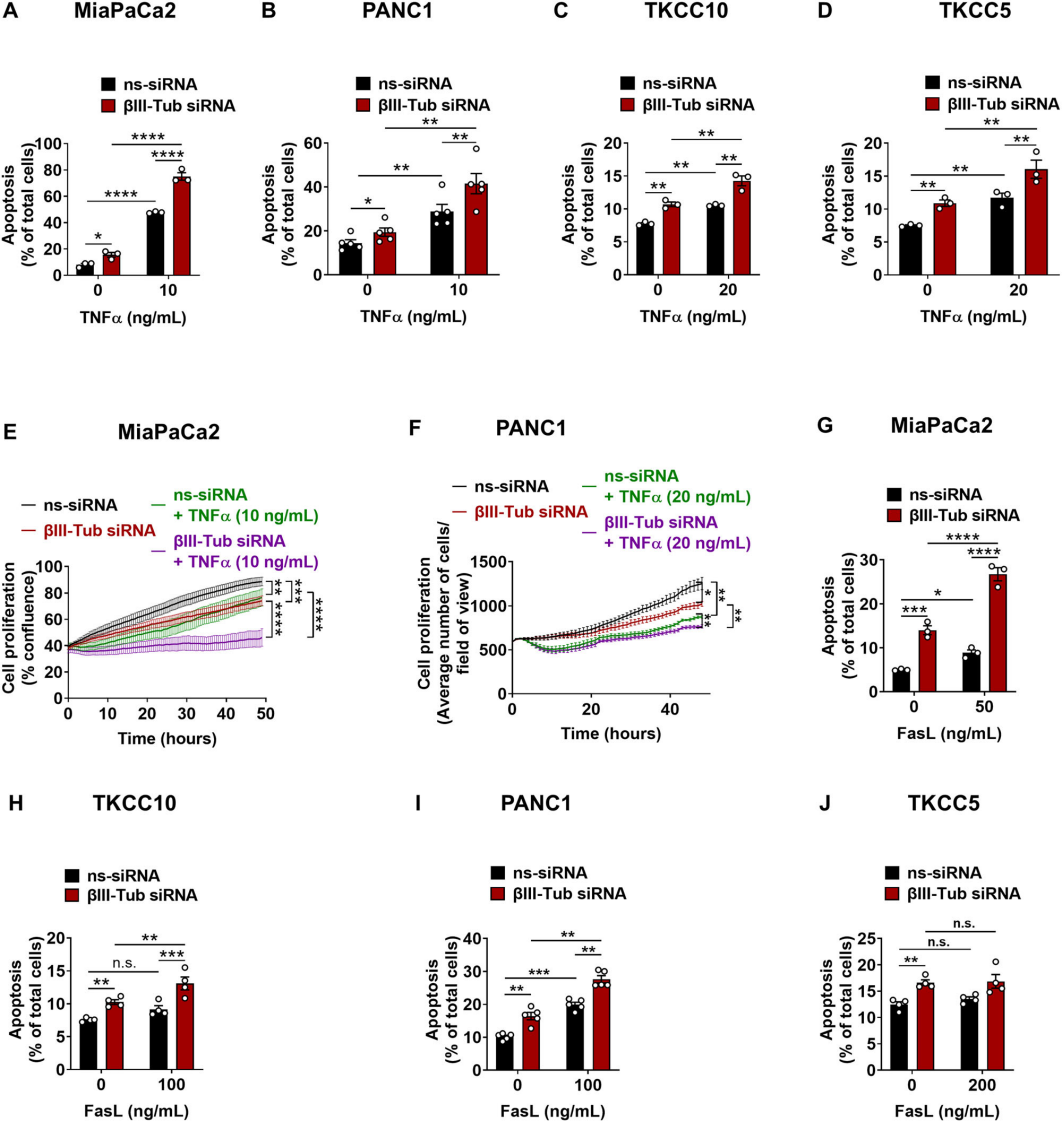
Knockdown of βIII-tubulin in PDAC cells increased sensitivity to TNFα and FasL. Apoptosis (Annexin V/DAPI) in MiaPaCa2 (*n* = 3) (**A**), PANC1 (*n* = 5) (**B**), TKCC10 (*n* = 3) (**C**) and TKCC5 **(D)** PDAC cells transfected with ns-siRNA or βIII-Tub siRNA and treated ± TNFα. IncuCyte® S3 live-cell analysis of (**E**) MiaPaCa2 cells (*n* = 4) and (**F**) PANC1 cells (*n* = 5; standardised to ensure equal starting cell count across independent experiments) with βIII-tubulin knockdown ±TNFα. Apoptosis measured in MiaPaCa2 (*n* = 3) (**G**), TKCC10 (*n* = 4) (**H**), PANC1 (n = 5) (**I**), or TKCC5 (n = 4) (**J**) cells transfected with ns-siRNA or βIII-tubulin siRNA and treated ± FasL. Bars represent mean of n ≥ 3 independent experiments (data points shown from independent experiments) ± SEM. Asterisks indicate significance as assessed by one-way ANOVA (*p ≤ 0.05, **p ≤ 0.01, ***p ≤ 0.001, ****p ≤ 0.0001; n.s.: non-significant).

We tested if βIII-tubulin silencing could enhance the anti-cancer cell effects of extrinsic apoptosis TNFα, and FasL. βIII-tubulin knockdown combined with TNFα induced apoptosis to a greater extent than either treatment alone in multiple PDAC cell lines (Fig. 3A–D). The cumulative effect over time was reduction of proliferation with the combination treatment, compared to single treatments and controls (Fig. 3E, F). Likewise, βIII-tubulin knockdown enhanced FasL-induced apoptosis, relative to single treatments and controls in MaPaCa-2, PANC1, and TKCC10 PDAC cell lines (Fig. 3G–I), but interestingly did not affect TKCC5 sensitivity to FasL (Fig. 3J). Unlike the other PDAC cell lines, TKCC5 showed no sensitivity to FasL alone (Fig. 3J), suggesting βIII-tubulin knockdown mediated sensitisation to FasL requires at least some level of basal FasL sensitivity.

### Knockdown of βIII-tubulin in PDAC cells triggered death receptor 5 (DR5) clustering

**Fig. 4:**
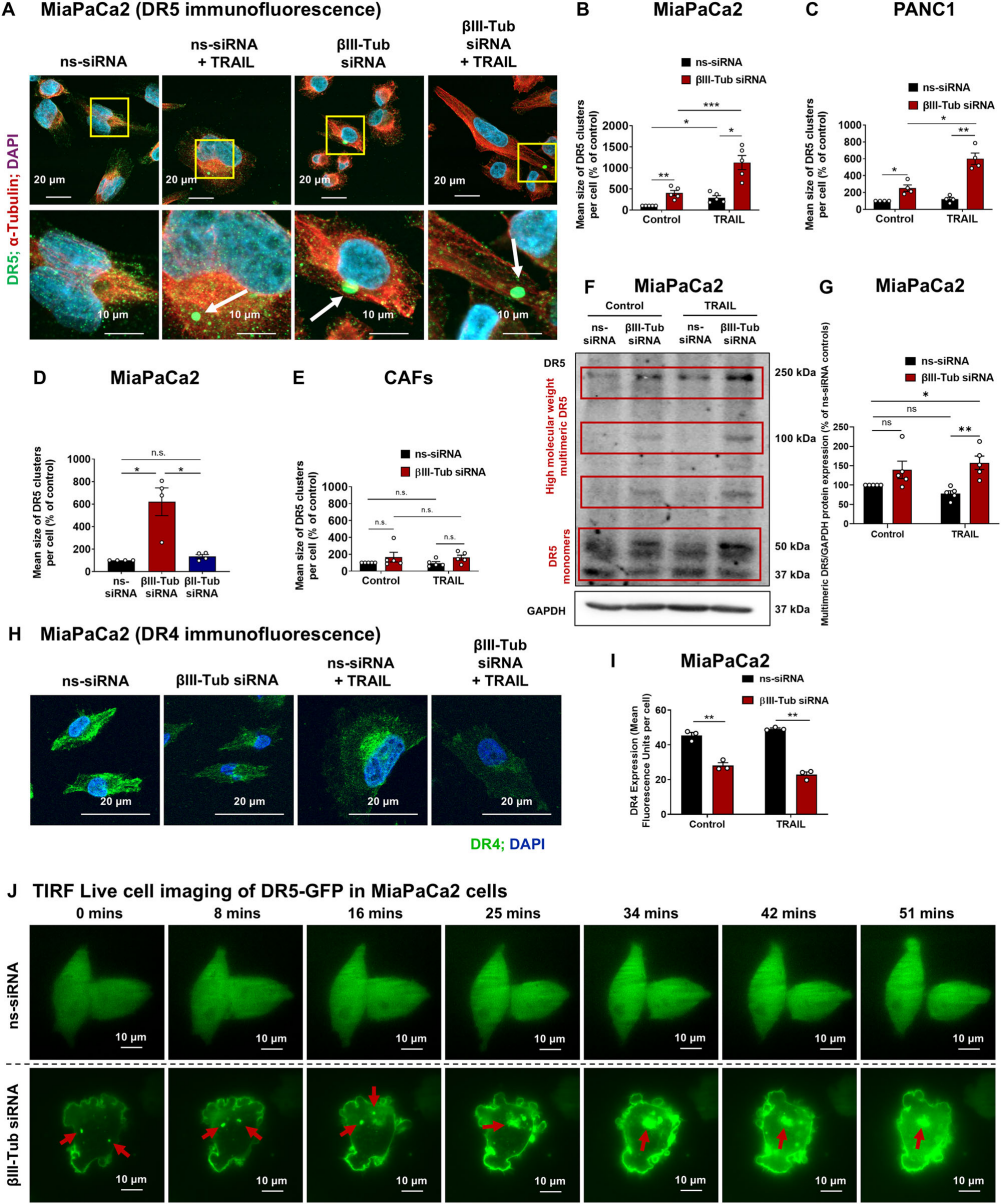
Knockdown of βIII-tubulin in PDAC cells triggered DR5 clustering at the plasma membrane. **A** Immunofluorescence staining for DR5 and α-tubulin in MiaPaCa2 cells transfected with non-silencing (ns) or βIII-tubulin (βIII-Tub) siRNA and treated ±TRAIL (250 ng/mL, 2 h). βIII-tubulin knockdown and TRAIL triggered formation of large membrane clusters of DR5 (white arrows). Merged images shown again for reference in Supplementary Fig. 11A with individual channel images. Quantification of DR5 cluster size (from z-stack maximum intensity projections) in MiaPaCa2 (*n* = 5) (**B**, **D**), PANC1 (*n* = 4) (**C**) and CAFs (*n* = 5) (**E**) cells transfected with ns-siRNA, βIII-tubulin siRNA, or βII-tubulin siRNA and co-treated with TRAIL. **F**, **G** Western blot (non-reducing conditions) using protein extracted from MiaPaCa2 cells transfected with ns- or βIII-tubulin siRNA and treated ±TRAIL (250 ng/mL; 2 h). **F** shows representative photos of *n* = 5 independent experiments, while **G** shows quantification of high molecular weight multimeric DR5 (>50 kDa) with GAPDH used as a loading control. **H** Immunofluorescence for DR4 in MiaPaCa2 cells showed with βIII-tubulin silencing + TRAIL. **I** Quantification of DR4 mean fluorescence intensity per cell (n = 3 independent experiments). Mean fluorescence intensity per cell was quantified using ImageJ software. **J** Live imaging of GFP-tagged DR5 on the cell membrane of MiaPaCa2 cells using total internal reflection (TIRF) microscopy. Cells were imaged 48 h post-transfection. Cells with βIII-tubulin knockdown that had visible DR5 membrane clustering (red arrows) showed characteristic features of apoptosis, such as membrane blebbing. See Supplementary Movie 1 for video of live cell imaging. Bars represent mean of *n* ≥ 3 independent experiments for cancer cells and independent patient-derived cells for CAFs (data points shown from independent experiments) ± SEM. Asterisks indicate significance as assessed by one-way ANOVA (*p ≤ 0.05, **p ≤ 0.01, ***p ≤ 0.001; n.s.: non-significant).

DR5 requires clustering at the cell membrane for activation of extrinsic apoptosis [25] and can bind to microtubules [26]. Using immunofluorescence, we observed that βIII-tubulin knockdown, exposure to TRAIL, or a combination of both in PDAC cells, triggered large membrane clusters of DR5 (Fig. 4A and Supplementary Fig. 5A, B). The combination also increased average cluster size relative to controls, to a significantly greater extent than either treatment alone (Fig. 4B, C). Interestingly, knockdown of βII-tubulin, which is also highly expressed in PDAC cells and has high structural homology to βIII-tubulin, did not promote DR5 clustering (Fig. 4D and Supplementary Fig. 5C). Knockdown of βIII-tubulin in pancreatic CAFs also failed to induce DR5 clustering (Fig. 4E and Supplementary Fig. 6A). Western blot for DR5 in PDAC cells where surface proteins had been crosslinked, showed that βIII-tubulin silencing had no effect on monomeric DR5 (37–50 kDa) (Fig. 4F). In contrast, cells treated with βIII-tubulin siRNA alone or βIII-tubulin siRNA + TRAIL had increased multimeric DR5 clusters (50–250 kDa), relative to control siRNA or TRAIL alone (Fig. 4F, G). βIII-tubulin silencing did not trigger clustering of DR4, but rather an overall reduction with βIII-tubulin knockdown (Fig. 4H, I and Supplementary Fig. 6B). We assessed the effect of βIII-tubulin silencing on DR5 dynamics at the cell membrane, using PDAC cells stably expressing GFP-DR5 and live total internal reflection (TIRF) microscopy [27]. Live cell imaging confirmed the presence of clusters of DR5 at the membrane of cells with βIII-tubulin knockdown, relative to controls (Supplementary Movie 1 and Fig. 4J). Notably, DR5-GFP membrane clustering was associated with apoptotic features such as membrane blebbing (Supplementary Movie 1 and Fig. 4J).

### βIII-tubulin regulates death receptor 5 dynamics in PDAC cells

**Fig. 5:**
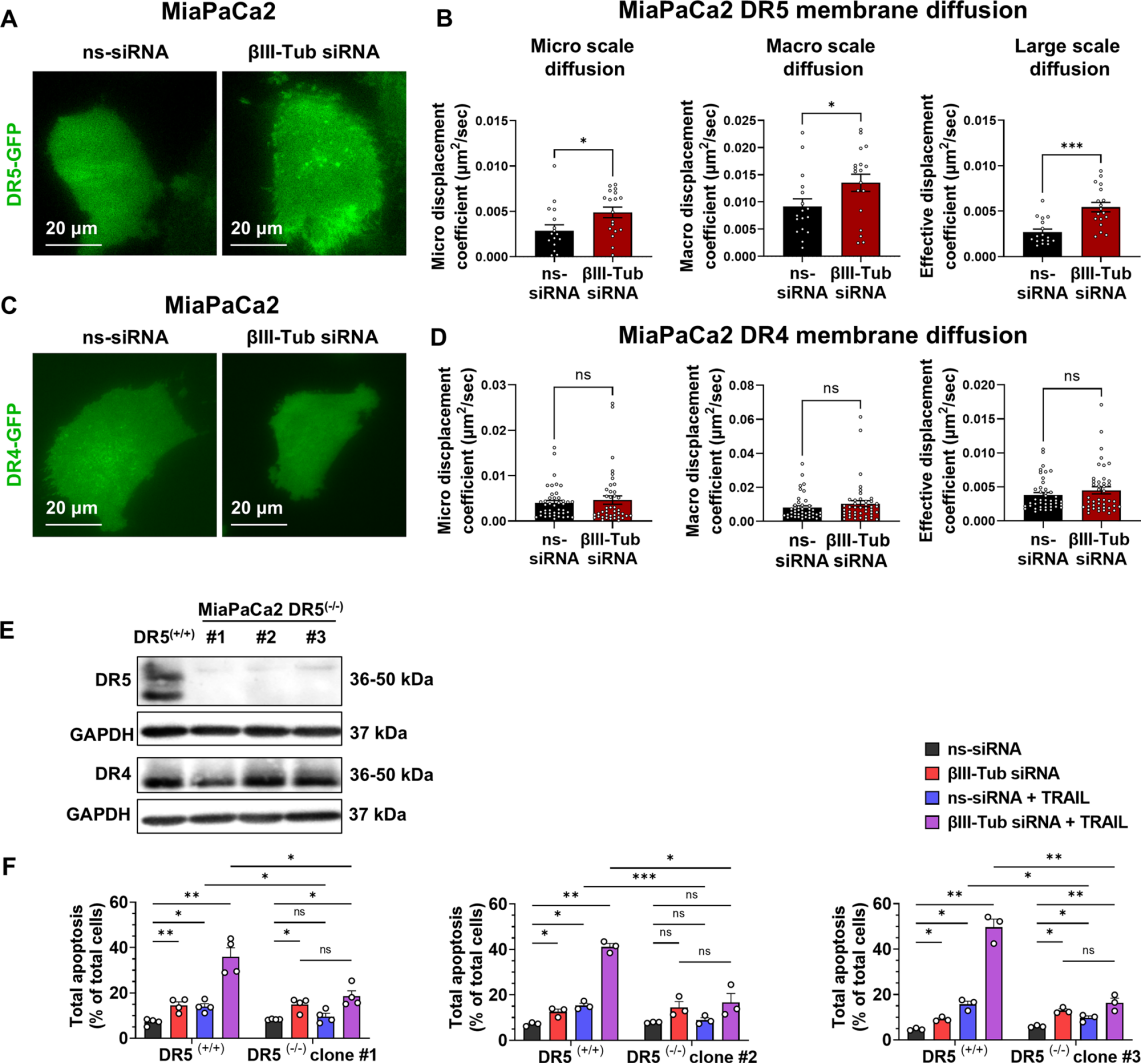
βIII-tubulin exerts its effect on TRAIL-induced extrinsic apoptosis via the DR5 receptor. Total internal reflection microscopy (TIRF) of the cell membrane of MiaPaCa2 cells expressing GFP-tagged DR5 (DR5-GFP) (**A**) or GFP-tagged DR4 (DR4-GFP) (**C**) following βIII-tubulin knockdown. Quantification of GFP-DR5 (**B**) and GFP-DR4 (**D**) membrane diffusion using live imaging in MiaPaCa2 cells with TIRF microscopy and k-space image correlation spectroscopy. See Supplementary Movie 2 for video of live cell imaging. Each data point indicates diffusion coefficient from analysis of a single cell. **E** MiaPaCa2 cells were generated with CRISPR knockout of DR5. Western blot shows knockout of DR5 in 3 subclones (DR5^(-/-)^) compared to wild-type (DR5^(+/+)^) controls and no compensation in DR4 protein levels. Protein was loaded onto two gels to allow imaging of both DR4 and DR5. Membranes were re-probed for GAPDH as a loading control. **F** MiaPaCa2 cells with wild-type DR5 or DR5 knockout were transfected with ns-siRNA or βIII-tubulin siRNA and treated ±TRAIL (10 ng/mL). Apoptosis measured with flow cytometry for Annexin V/DAPI. Bars represent mean of *n* ≥ 3 independent experiments (data points shown from independent experiments) ± SEM. Asterisks indicate significance as assessed by one-way ANOVA (**F**) or two-tailed *t*-test (**C**, **D**) (*p ≤ 0.05, **p ≤ 0.01, ***p ≤ 0.001; n.s.: non-significant).

To measure the movement of DR5 clusters at the cell membrane of PDAC cells, we repeated TIRF live-cell imaging with higher temporal resolution (Supplementary Movie 2). We quantified DR5 dynamics using k-space image correlation spectroscopy and demonstrated significantly increased diffusion coefficients (faster movement) across all spatial scales of movement with βIII-tubulin siRNA compared to controls (Fig. 5A, B). DR4 membrane diffusion coefficients were unaffected by βIII-tubulin knockdown in PDAC cells (Fig. 5C, D). To assess the dependence of βIII-tubulin knockdown-mediated sensitisation to TRAIL on DR5, we generated DR5 knockout PDAC cells (Fig. 5E), then measured apoptosis following βIII-tubulin knockdown + TRAIL. DR5 knockout in three different PDAC cell clones had no effect on apoptosis induced by βIII-tubulin knockdown alone, but completely blocked βIII-tubulin siRNA-induced TRAIL sensitisation (Fig. 5F). We also demonstrated that βIII-tubulin silencing in PDAC cells increased sensitivity to a DR5-specific agonist to a greater extent compared to a DR4-specific agonist (Supplementary Fig. 7), supporting a model where βIII-tubulin regulates sensitivity to TRAIL via DR5.

### Pro-apoptotic effects of βIII-tubulin silencing in PDAC cells are enhanced in the presence of cancer-associated fibroblasts (CAFs)

**Fig. 6:**
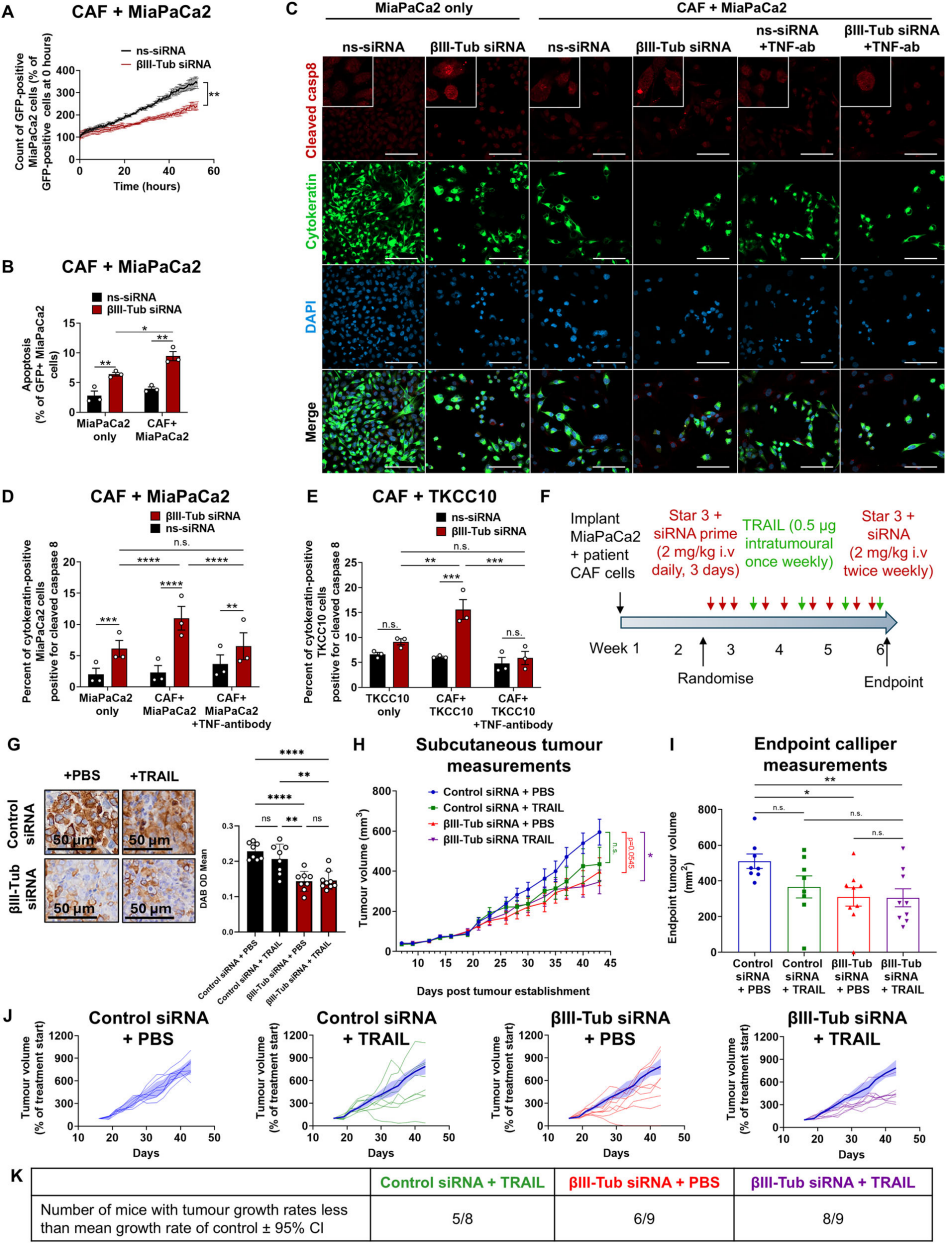
βIII-tubulin knockdown exerted pro-apoptotic effects in PDAC cells when co-cultured with patient-derived CAFs in vitro and improved the number of mouse responders to TRAIL in vivo. **A** IncuCyte® S3 live-cell analysis (proliferation) of GFP-MiaPaCa2 cells transfected with non-silencing (ns) or βIII-tubulin (βIII-Tub) siRNA and co-cultured with cancer-associated fibroblasts (CAFs) (*n* = 3 independent experiments with CAFs from 3 PDAC patients). **B** Apoptosis (AnnexinV/DAPI) in GFP-MiaPaCa2 cells with βIII-tubulin knockdown and co-cultured with CAFs (*n* = 3). **C–E** Immunofluorescence was used to measure the percentage of cleaved caspase 8 positive cells in cytokeratin positive PDAC cells when cultured alone or in the presence of CAFs. Cells were treated with or without TNFα-neutralising antibody (2 μg/mL). QuPath software was used to measure the percentage of cleaved caspase 8 positive cells in cytokeratin positive MiaPaCa2 **D** or TKCC10 **E** cells (*n* = 3). Representative images **C** show immunofluorescence staining in MiaPaCa2 cells. All scale bars represent 100 μm. An equal number of total cells (MiaPaCa2 + CAFs) was seeded into wells. Bars represent mean of *n* ≥ 3 independent experiments (data points shown from independent experiments) ± SEM. **F** Treatment regimen used in subcutaneous mouse model. **G** Immunohistochemistry for βIII-tubulin and quantification of staining intensity (OD mean) in subcutaneous tumour sections at endpoint. **H** Tumour volume calliper measurements in situ recorded 3 times per week. Symbols represent mean ± SEM tumour volume per treatment group. **I** Ex vivo calliper measurements at endpoint harvest. Bars represent mean ± SEM tumour volume with symbols representing individual mice. **J** Tumour growth rates of individual mice per treatment group. Each graph is overlayed with the mean growth rate of control siRNA + PBS treated mice with 95% confidence interval represented by the blue shade. **K** Table summary of number of responders (tumour growth rate that was less than the mean growth rate of control mice ± 95% confidence interval) in each treatment arm. Asterisks indicate significance as assessed by one-way ANOVA **(B–D**, **G–I)** or two-tailed paired *t*-test (**A**) (*p ≤ 0.05, **p ≤ 0.01, ***p ≤ 0.001, ****p ≤ 0.0001; n.s.: non-significant).

We co-cultured CAFs with GFP-expressing PDAC cells and tracked GFP-positive PDAC cell proliferation following βIII-tubulin knockdown in tumour cells only. βIII-tubulin knockdown reduced proliferation of MiaPaCa2 (Fig. 6A and Supplementary Fig. 8A), TKCC5 (Supplementary Fig. 8B) and TKCC10 PDAC cells (Supplementary Fig. 8C) when cultured in the presence of CAFs. Using the same co-culture model, we demonstrated that apoptosis induced by βIII-tubulin knockdown in MiaPaCa2 cells was significantly enhanced in direct co-culture (Fig. 6B) and indirect co-culture (Supplementary Fig. 8D) with CAFs. Immunofluorescence for cleaved caspase 8 in cytokeratin-positive PDAC cells in MiaPaCa2:CAF co-cultures showed that βIII-tubulin knockdown in MiaPaCa2 cells cultured in the presence of CAFs significantly increased cleaved caspase 8 in PDAC cells, relative to those in the absence of CAFs (Fig. 6C–E). Notably, the addition of TNFα neutralising antibodies blocked the CAF-induced increase in caspase 8 cleavage in PDAC cells with βIII-tubulin knockdown, suggesting this effect was in part driven by TNFα secretions from CAFs (Fig. 6C–E).

### βIII-tubulin knockdown in mouse subcutaneous PDAC tumours increased the number of responders to TRAIL therapy

Mice with subcutaneous PDAC tumours (MiaPaCa2 cells and CAF co-injection) were treated intravenously with polymeric nanoparticles complexed to control siRNA or βIII-tubulin siRNA, and co-treated with intratumoural TRAIL or vehicle (Fig. 6F). βIII-tubulin silencing was confirmed by immunohistochemistry (Fig. 6G). Both βIII-tubulin silencing alone and in the presence of TRAIL significantly reduced tumour volume compared to control siRNA + PBS treated mice (Fig. 6H–I). TRAIL treatment alone did not significantly reduce tumour volume compared to controls, and TRAIL combined with βΙΙΙ-tubulin siRNA did not further reduce tumour volume compared to mice with βΙΙΙ-tubulin knockdown alone (Fig. 6H–I). However, we observed that βIII-tubulin silencing combined with TRAIL increased the number of responders (based on growth rates less than the mean growth rate of control siRNA + PBS treated mice) relative to either single treatment group (Fig. 6J, K). Results implied that the main therapeutic benefit of this combination treatment in vivo is to enhance anti-tumour effects against a subset of tumours that were unresponsive to either treatment alone (22% of tumours).

### Combination of βIII-tubulin silencing and TRAIL treatment exerted anti-cancer effects in patient derived PDAC tumour explants

**Fig. 7:**
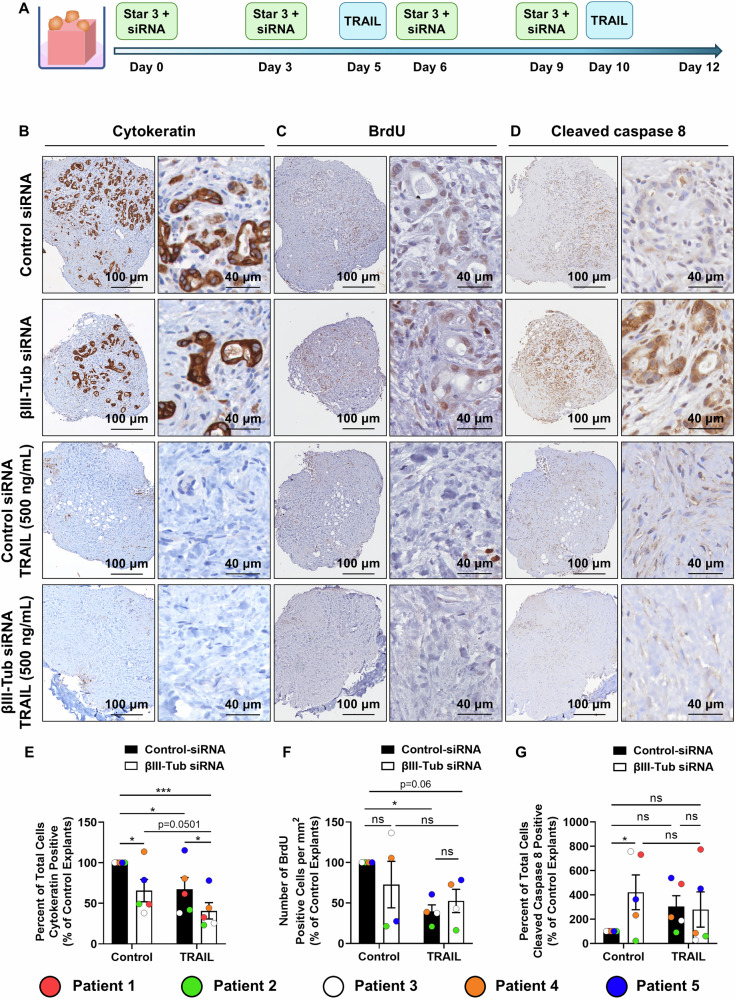
βIII-tubulin combined with TRAIL decreased tumour cell numbers in tumour explants from PDAC patients, with patient specific effects on extrinsic apoptosis and cell proliferation. **A** Treatment schedule was used to assess effects of βIII-tubulin (βIII-Tub) silencing combined with TRAIL in PDAC tumour explants. Patient 3 representative immunohistochemistry staining of cytokeratin (tumour cell marker) (**B**), bromodeoxyuridine (BrdU) (proliferation marker) (**C**), and cleaved caspase 8 (extrinsic apoptosis marker) **(D)** at low and high magnification. Quantification of staining from whole tumour explants was performed on QuPath for cytokeratin (**E**), BrdU (**F**), and cleaved caspase 8 (**G**) and data combined from *n* = 5 patients, taking the average quantification of 2–4 explants from each patient. Cell proliferation was not assessed in Patient 1. Each symbol represents the mean of 2-4 explants from each patient. Bars represent mean ± SEM. Asterisks indicate significance as assessed by one-way ANOVA (*p ≤ 0.05, **p ≤ 0.01, ***p ≤ 0.001; ns: non-significant).

Next, we examined the effects of silencing βIII-tubulin in combination with TRAIL in a human patient-derived PDAC tissue explant model which maintains 3D spatial multicellular organisation of PDAC tissue [24]. Tissue explants from five PDAC patients were treated with polymeric nanoparticles + control-siRNA or βIII-tubulin siRNA and TRAIL over 12 days (Fig. 7A). βIII-tubulin knockdown was confirmed by immunohistochemistry (Supplementary Fig. 9A–C). Immunohistochemistry for cytokeratin (tumour cells), BrdU (proliferation), and cleaved caspase 8 (extrinsic apoptosis) was performed on endpoint tissue explant sections to determine therapeutic response (Supplementary Figs. 10–14–representative images of immunohistochemistry from Patient 3 shown in Fig. 7B, D). Tumour cell frequency was markedly reduced in 4/5 explants with βIII-tubulin silencing alone and TRAIL treatment alone compared to untreated control-siRNA explants (Fig. 7E). Importantly, combination of βIII-tubulin silencing with TRAIL reduced tumour cell frequency in all the explants compared to untreated control-siRNA explants and led to the greatest reduction in tumour cell numbers compared to single treatments (Fig. 7E). Cell proliferation was reduced in tissue explants from 2/4 patients treated with βIII-tubulin siRNA, 4/4 patients treated with TRAIL, and 4/4 patients treated with combination, compared to controls (Fig. 7F). Cleaved caspase 8 positive cells increased in tissue explants with βIII-tubulin knockdown in 4/5 patients, with TRAIL treatment in 4/5 patients, and with the combination in 2/5 patients, compared to controls (Fig. 7G). In contrast to tumour cell frequency, significance for cell proliferation and cleaved caspase 8 was only observed for TRAIL alone and βIII-tubulin knockdown alone, respectively, due to the heterogeneity of patient responses. The effects of βIII-tubulin siRNA and TRAIL treatment on αSMA-positive CAFs were patient-specific and on average, not significant (Supplementary Fig. 15). In addition, H&Es (Supplementary Fig. 16) showed that tissue architecture was not destroyed, implying this was not a broadly toxic combination.

### High βIII-tubulin expression in PDAC tumour cells correlated with poor overall survival, and its high expression in the stroma was independently prognostic of poor overall survival

**Fig. 8:**
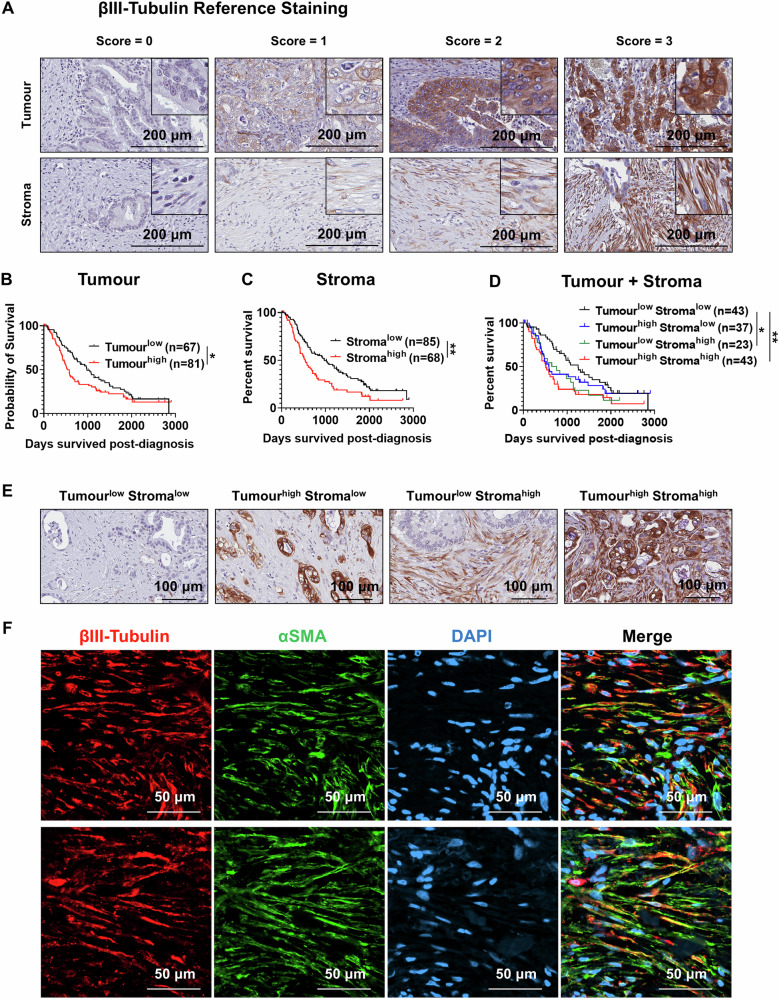
High βIII-tubulin expression correlates with PDAC patient poor overall survival. Human PDAC sections were stained for βIII-tubulin and tumour and stromal staining scored by three independent scorers. **A** Representative immunohistochemistry images showing four different scores of βIII-tubulin staining intensity in tumour and stromal regions. Kaplan–Meier survival curves show correlation between βIII-tubulin expression in tumour cells (**B**), stroma (**C**), or both tumour and stroma (**D**) with overall patient survival (days post diagnosis). Total patient numbers per group are indicated in legends. Asterisks indicate significance based on univariate analysis, log-rank test (*p ≤ 0.05; **p ≤ 0.01). **E** Representative images showing examples of combined tumour and stroma score groups. **F** Co-immunofluorescence in 2 human PDAC samples to assess colocalisation of βIII-tubulin in α-smooth muscle actin (αSMA) positive cancer associated fibroblasts (CAFs).

We assessed the prognostic value of βIII-tubulin in tumour and stromal compartments within the APGI International Cancer Genome Consortium (ICGC) PDAC cohort. Patient characteristics are in Supplementary Table 1. We showed that high expression of βIII-tubulin in tumour cells (55% of patients) correlated with poor overall survival (Fig. 8A-B, Supplementary Table 1). Furthermore, we showed for the first time that high expression of βIII-tubulin in the stromal compartment (44% of patients) of PDAC was independently prognostic (HR = 2.163, *p* = 0.009) of poor overall survival (Fig. 8A, C and Supplementary Table 2). We also found that high expression of βIII-tubulin in both tumour and stroma was associated with the worst patient overall survival (Fig. 8D, E). Co-immunofluorescence confirmed that βIII-tubulin is expressed in α-smooth muscle actin (αSMA)-positive CAFs (Fig. 8F).

## Discussion

Our findings identify βIII-tubulin as a death switch in PDAC, whose inhibition enhances PDAC cell sensitivity to microenvironment-derived (TNFα) and immune cell-derived (FasL) anti-tumour factors, and a tumour-specific therapeutic (TRAIL), which opens novel therapeutic avenues to improve treatments for PDAC patients. We observed that βIII-tubulin knockdown in PDAC cells induced both intrinsic and extrinsic apoptosis. However, only caspase 8 inhibition blocked βIII-tubulin knockdown-induced apoptosis. In the context of extrinsic apoptosis, cancer cells are classified as either type I or type II cells [28]. Type I cells are capable of inducing executioner caspases 3 and 7 cleavage with caspase 8 activation alone, whereas type II cells require crosstalk into the intrinsic mitochondrial pathway, through cleavage of Bid, to activate apoptosis [28]. While PDAC cells are reported as type II cells [28], our approach was still able to induce apoptosis when caspase 9 was inhibited, thus overcoming a key apoptosis resistance pathway (defective mitochondrial signalling) in type II cancer cells [28]. Previously, we showed that stable βIII-tubulin knockdown decreased orthotopic PDAC tumour growth in mice [12]. In this study, we demonstrated that therapeutic delivery of βIII-tubulin siRNA using polymer nanoparticles can delay orthotopic PDAC tumour growth [23] and increase intratumoural caspase 8 cleavage, illustrating the potential clinical utility of βIII-tubulin inhibition for PDAC.

Extrinsic apoptosis (caspase 8-dependent) can be activated by binding of TRAIL, TNFα, or FasL to their receptors [29]. βIII-tubulin knockdown in PDAC cells enhanced cell death and anti-proliferative effects in the presence of these ligands. In contrast, βIII-tubulin did not regulate apoptosis or TRAIL sensitivity in pancreatic CAFs, suggesting this role is specific to neoplastic cells. TRAIL is an anti-cancer drug which binds DR4/5 receptors on cancer cell membranes to activate extrinsic apoptosis [28]. DR5 requires clustering at the cell membrane to initiate extrinsic apoptosis [25]. To investigate the mechanism driving TRAIL sensitisation, we first confirmed that the human recombinant TRAIL we utilised was as effective as trimeric TRAIL (forced trimerisation through isoleucine zipper) at inducing apoptosis, indicating that human recombinant TRAIL is largely trimeric in culture. We observed that βIII-tubulin knockdown increased DR5 (but not DR4) clustering, which was markedly enhanced in the presence of TRAIL. βIII-tubulin knockdown also induced higher levels of lateral DR5 movement across the plasma membrane than control cells. The connection between βIII-tubulin and DR5 in these cells was further illustrated by three findings: (i) Knockdown of βII-tubulin (close structural homology to βIII-tubulin; upregulated in PDAC) did not induce DR5 clustering or extrinsic apoptosis; (ii) βIII-tubulin knockdown in PDAC cells induced greater sensitisation to apoptosis induced by a DR5 agonist over a DR4 agonist; and (iii) DR5 knockout in PDAC cells abolished βIII-tubulin knockdown-mediated sensitisation to TRAIL. Interestingly, we observed that apoptosis induced by βIII-tubulin knockdown alone was unaffected by DR5 knockout, suggesting that βIII-tubulin knockdown can trigger apoptosis independently of DR5. Future studies will investigate whether this is due to increased reliance on DR4 in this setting or whether βIII-tubulin regulates downstream factors that can drive extrinsic apoptosis independent of extrinsic cues. We observed similar responses in NSCLC cell lines (another solid tumour that overexpresses βIII-tubulin), indicating that this potential therapeutic avenue may be conserved across a range of tumours. Our results identify a novel βIII-tubulin/DR5 axis whereby βIII-tubulin hinders DR5 clustering and activation at the PDAC cell membrane. Future investigations will assess whether this involves: (i) direct regulation of DR5 trafficking by interactions with cytoskeletal βIII-tubulin; (ii) indirect regulation of DR5 activity through cellular reprogramming. Indirect regulation may include alteration of decoy receptor levels or trafficking, which can inhibit DR5-mediated caspase 8 activation or which can titrate TRAIL away from the receptor [30].

Clinical trials of TRAIL/TRAIL receptor agonists in cancer showed that they lack therapeutic efficacy due to poor bioavailability or development of resistance [28, 31]. We demonstrated that therapeutic delivery of βIII-tubulin siRNA to PDAC xenografts, using polymer nanoparticles [23, 32], significantly reduced tumour growth and increased the frequency of TRAIL-responsive tumours. However, on average TRAIL sensitisation was not statistically significant in this setting. Future studies may require a more aggressive version of this regimen to observe sensitisation across all tumours in vivo. This approach could also benefit from nanoparticle encapsulation of TRAIL to enhance tumour bioavailability or to deliver TRAIL mRNA for sustained in vivo expression. Alternatively, our findings may indicate that in vivo, TRAIL sensitisation may only be observed in tumour subsets that show minimal response to either treatment alone. We also acknowledge the limitations of our in vivo model due to the sub-cutaneous setting (used to maximise TRAIL intratumoural bioavailability via direct delivery) and immune compromised hosts. The latter is particularly relevant as our in vitro data suggests βIII-tubulin silencing may sensitise PDAC cells to FasL from cytotoxic immune infiltrate. Future work will assess our therapeutic approach in orthotopic and syngeneic models of the disease. We also demonstrated in vitro that sensitisation to FasL with our approach requires at least some level of FasL sensitivity to work, and should be considered in design of future in vivo models.

We similarly showed that both the single treatment arms and combination treatment arms in our patient-derived PDAC tumour explant model [24] significantly reduced tumour cell frequency, and importantly, that the combination treatment arm resulted in a greater reduction in tumour cell numbers on average, than either treatment alone. The extent of this effect was patient-dependent. There were no consistent differences in caspase 8 cleavage or cell proliferation with combination treatment compared to single treatments, likely a consequence of heterogeneous sensitivity to treatments between patients. For example, in samples highly sensitive to βIII-tubulin siRNA, we may be observing the maximal induction of caspase 8 cleavage, explaining why further induction with TRAIL may not be observed. Like our in vivo findings, our results indicate the need to identify a signature of biomarkers to predict subsets of patients most likely to benefit from this combination therapy. Reductions in αSMA^+^ CAFs in our explants were patient dependent and, on average, not significant, suggesting this approach was unlikely to lead to stromal depletion in a clinical setting.

Finally, we assessed prognostic and functional significance of βIII-tubulin in PDAC stroma. βIII-tubulin was previously shown to be overexpressed in surgically resectable PDAC patients [13], and in patients with metastatic PDAC, high βIII-tubulin was associated with decreased progression-free survival [14, 33]. Our study in a surgically-resectable PDAC patient cohort showed that high βIII-tubulin in either tumour or stromal compartments correlated with decreased overall-survival (independently prognostic in stroma). Additionally, we found high expression of βIII-tubulin in both tumour and stroma predicted the worst overall survival. We subsequently showed that βIII-tubulin knockdown in CAFs in vitro reduced their proliferation by inducing senescence (but not apoptosis). Studies have uncovered the complexity of CAF cell heterogeneity within PDAC stroma [3, 4, 34, 35]. Future work should investigate how inhibition of βIII-tubulin in PDAC cells and CAFs are influenced by different stromal sub-states and whether particular stromal signatures can predict response to βIII-tubulin inhibition. In the PDAC microenvironment, CAFs secrete TNFα as a pro-tumour paracrine signal [36]. TNFα binds to its receptor (TNF-R1) to induce apoptosis but is also capable of activating the NF-κB pathway to promote cell proliferation and tumour progression [37]. In PDAC cell-CAF co-cultures, we showed that anti-proliferative and pro-apoptotic effects induced by βIII-tubulin knockdown in PDAC cells were markedly enhanced in the presence of CAFs, and that this was mediated via TNFα secretion from CAFs, thus potentially identifying an innovative strategy to reprogram a microenvironmental pro-tumour signal into a death signal.

Collectively, this work represents a breakthrough in our understanding of βIII-tubulin biology and developing therapeutic strategies against βIII-tubulin. It has provided new mechanistic insights into the pro-survival role of βIII-tubulin in PDAC and more broadly, as a therapeutic target in poor outcome cancers. For decades, βIII-tubulin has been established as a cancer therapeutic target [15–17, 20, 21, 38–46]. We discovered an unexpected role of βIII-tubulin as a brake on membrane clustering of DR5 and activation of extrinsic apoptosis. These findings provide momentum to develop βIII-tubulin targeting therapies for PDAC, especially clinic-ready nanoparticles to deliver βIII-tubulin siRNA, together with drugs that induce extrinsic apoptosis.

## Materials and methods

### Cell culture

All tissue culture reagents were purchased from Life Technologies. Human PDAC (MiaPaCa2, PANC1, HPAF-II) and lung cancer (H460, A549) cell lines were obtained from American Type Culture Collection (ATCC) and cultured as described [12, 32, 47]. PDAC patient-derived TKCC cells isolated from patient-derived xenografts were cultured as described [48]. Patient derived CAFs were isolated from PDAC tumours by explant outgrowth culture and used within 12 passages as described [49, 50]. The purity of CAFs was assessed as described [51]. All experiments using patient derived CAFs were approved by UNSW Sydney human ethics committee (approvals: HC14039, HC180973) and all experiments were performed in accordance with the relevant guidelines and regulations. All patients provided written informed consent. All cells tested negative for mycoplasma monthly.

### siRNA transfection

PDAC and lung cancer cells were transfected with siRNA 24 h post seeding as described [32, 47]. All siRNAs used are listed in Supplementary Table 3. βIII-tubulin siRNA specificity has previously been demonstrated in both PDAC and lung cancer cells [15, 18].

### Quantitative real-time PCR

Total cellular RNA was extracted from transfected PDAC cells and βIII-tubulin gene expression was measured using quantitative PCR as described [12, 32, 47].

### Western blot analysis

Whole cell lysates were prepared, quantitated, and loaded onto SDS-PAGE gels and transferred to nitrocellulose as described [12, 32, 47]. Nitrocellulose membranes were probed with primary and secondary antibodies listed in Supplementary Table 3. For analysis of TRAIL-receptor clustering, PDAC cells were treated with or without TRAIL (250 ng/mL) for 2 h, 72 h post-transfection. Following TRAIL treatment, proteins in live cells were crosslinked by incubation in 2 mM BS3 crosslinker (45 min, room temperature). BS3 was quenched in 40 mM Tris-HCl (pH 7.5) for 10 min, then protein was extracted as described above. Western blot was performed under non-reducing conditions, and transferred to a polyvinylidene fluoride (PVDF) membrane and probed with antibodies listed in Supplementary Table 3. Protein bands were visualised and quantified as described [32, 47]. All raw blots can be found in **Supplementary full western blots**.

### Quantification of apoptosis

Apoptosis was measured using flow cytometry for Annexin V-PE (BD Biosciences) and DAPI (Sigma-Aldrich, cat. D9542), as described [32, 47] and quantified using FlowJo v10 software. The gating strategy used for quantification of apoptosis is shown in Supplementary Fig. 17.

### Quantification of cell viability and cell proliferation

Cell viability was quantified by trypan blue exclusion assay as described [12, 32, 47]. Cell proliferation was measured on an IncuCyte® S3 Live-Cell Analysis System (Essen BioScience) as percent confluence or total cell count using the cell-by-cell analysis platform on IncuCyte® software. An example phase contrast image of MiaPaCa2 cells on the IncuCyte® S3 is shown in Supplementary Fig. 18A and the corresponding confluence mask (yellow) in Supplementary Fig. 18B.

### Drug and inhibitor treatments

Inhibitors of caspase 9 (Z-LEHD-FMK; Cat. 1149-1, BioVision) and caspase 8 (Q-IETD-OPh; Cat. 1176-1, BioVision) were used to treat MiaPaCa2 (20 μM inhibitors), PANC1 (50 μM inhibitors) and H460 (50 μM inhibitors) cells, 48 h post-siRNA transfection. Apoptosis was measured 24 h later. Inhibitors were used at doses validated in PDAC cells [52]. Both inhibitors were dissolved in dimethyl sulfoxide (DMSO) and DMSO vehicle was used as the 0 μM control. Inhibitor activity was validated in Supplementary Fig. 19A, B. For co-treatment with TRAIL, caspase 8/9 inhibitor was added to cells 1 h before TRAIL addition (10 ng/mL in MiaPaCa2), 48 hours post-transfection. Recombinant human TRAIL (Abcam, cat. Ab9960) and TNFα (Abcam, cat. ab9642) were reconstituted in sterile H_2_O. FasL (Sigma-Aldrich, cat. SRP3036) was dissolved in sterile 1x PBS with 0.1% bovine serum albumin. Trimeric TRAIL (LSBio, cat. LS-G3910-10) was reconstituted in 100 μL sterile water with 0.1% bovine serum albumin (BSA). DR4 agonist (Creative Biolabs, cat. TAB-H48) and DR5 agonist (Creative Biolabs, cat. TAB-203) were diluted in culture medium. Cells were treated at concentrations indicated in figures, 48 h post-transfection with siRNA. Doses selected based on a 50% increase in apoptosis. Apoptosis or cell viability was measured 24 h post-treatment, with the exception of FasL in MiaPaCa2 cells (48 h post-treatment). See Supplementary methods for more details.

### Measurement of caspase activity

MiaPaCa2 cells were seeded into 96-well plates 24 h post-transfection at 5000 cells/well, as described [12]. 72 h post-transfection, CaspaseGlo assays [Promega, caspase 9 (cat. G8210) and or caspase 8 (cat. G8200)] were performed as per manufacturer instructions.

### Nanoparticle synthesis and preparation

Polymer-based miktoarm star polymer nanoparticles (Star 3) were synthesised and characterised before in vitro and in vivo use as described [23, 32].

### Orthotopic pancreatic cancer mouse model

8-week-old female BALB/c nude mice were used. All animal experiments were approved by the Animal Ethics Committee, UNSW (ACEC 12/7B; 13/130B; 18/54B). 1×106 MiaPaCa2 cells (with stable expression of luciferase) were implanted into the tail of the pancreas of mice as described [12]. PDAC tumours were allowed to develop for 4 weeks. Mice without tumours at 4 weeks post-implant were excluded from treatment and analysis. Mice were then randomised based on tumour luminescence as described [23, 32]. Star 3 + control siRNA (antisense: 5′-GAACUUCAGGGUCAGCUUGCCG) or βIII-tubulin siRNA (antisense: 5′-GCAGUUUUCACACUCCUUCUU) were administered intravenously at 4 mg/kg twice weekly for 4 weeks. Blinding was not possible during treatments due to staff availability. Staff performing tumour measurements were blinded to treatments. At endpoint, tumour volume was measured, and PDAC tissue was collected for assessment of βIII-tubulin knockdown by western blot and immunohistochemistry, as described [12, 23, 32].

### Immunohistochemistry analysis of mouse orthotopic and sub-cutaneous pancreatic tumour sections

Paraffin-embedded tumour sections were stained with: (i) βIII-tubulin antibody (1:50; Biolegend, cat. 801202) as described [12, 23, 32]; (ii) cleaved caspase 8 primary antibody (1:100; Cell Signalling Technology, cat. #9496) and goat anti-rabbit biotinylated secondary (1:200; Vector Laboratories, cat. BA-1000). Quantification of cleaved caspase 8 and βIII-tubulin from representative regions was performed in QuPath v0.3.2 [53]. Isotype control antibodies were used at the same concentration as primary antibodies (Mouse IgG2A for βIII-tubulin; Rabbit IgG for cleaved caspase 8; Supplementary Fig. 20). See Supplementary methods for more details.

### Clonogenic assays

Twenty-four hours post-transfection, MiaPaCa2 cells were seeded at 300 cells/well, and HPAF-II cells at 500 cells/well in 6-well tissue culture plates, as described [12]. 24 hours post-seeding, cells were incubated with TRAIL (MiaPaCa2: 5 and 10 ng/mL; HPAF-II: 50 and 200 ng/mL) for 72 h. Colonies were stained and counted as described [12, 23, 32].

### Anoikis assays

Anoikis assays were performed as described [12]. Cells were re-seeded into the Poly-HEMA coated plates 24 h post-transfection. After a further 24 h, TRAIL (10 ng/mL) was added for a further 9 hours and apoptosis measured as described above. See Supplementary methods for more details.

### Senescence assay

Senescence was measured in CAFs using a β-Galactosidase assay, 72 h post-transfection with ns-siRNA or βIII-tubulin siRNA, according to manufacturer’s instructions (Cell Signalling Technologies, Cat. 9860). Representative images were taken per well using the Leica DM2500 light microscope, and percentage positive cells quantified using QuPath.

### DR4 / DR5 TRAIL receptor immunofluorescence

MiaPaCa2 and PANC1 cells were re-seeded into 8-well chamber slides (Ibidi, cat. 80826) at 10,000 cells/well, 24 h post transfection, cultured for a further 48 h then treated with TRAIL (250 ng/mL) for 2 h. Cells were fixed in 4% paraformaldehyde and immunofluorescence staining was performed as described [12], using antibodies described in Supplementary Table 3. Stained cells were mounted with Prolong Gold anti-fade mounting media with DAPI. Images were taken on a Zeiss LSM800 confocal microscope. DR4 and DR5 cluster size per cell was measured using the Analyse Particle function on ImageJ v1.52a (National Institutes of Health, Bethesda, Maryland, USA). See Supplementary Methods for more details.

### Live imaging of GFP-tagged death receptor 5 (DR5) in pancreatic cancer cells

MiaPaCa2 cells were lentivirally transduced with GFP-tagged DR5 (Origene, Cat. RC201588L4V) and GFP-positive cells sorted on a BD FACS Aria II. Sorted cells were transfected with ns-siRNA or βIII-tubulin siRNA and then re-seeded into 8-well Ibidi chamber slides (#1.5, 0.170 mm thickness, Ibidi, cat. 80826). At 48 h post-transfection, cells were imaged on a Zeiss Elyra 7 Lattice SIM^2^ microscope using a 63x/1.46 objective with total internal reflection (TIRF) imaging to visualise the cell membrane (TIRF mirror angle set to 66.25°). DR5-GFP diffusion was quantified using k-space image correlation spectroscopy (kICS), as previously described [54–56]. See Supplementary methods for more details.

### CRISPR knockout of DR5 in pancreatic cancer cells

CRISPR knockout of DR5 was performed in MiaPaCa2 cells using DR5 (TNFRSF10B) Human Gene Knockout Kit (OriGene, cat. KN201588LP), according to manufacturer’s instructions. Cells were co-transfected with two different gRNA sequences to increase efficiency, selected in 10 μg/mL puromycin for two weeks then sub-cloned (single cell colonies). Knockout confirmed by Western blot as described above.

### Cancer-associated fibroblast and pancreatic cancer cell co-culture

PDAC cells (MiaPaCa2, TKCC5, TKCC10) with stable expression of GFP were transfected with control or βIII-tubulin siRNA. After 24 h, transfected cells were directly co-seeded into 12 well plates with CAFs at a 3:1 ratio of CAF:PDAC cells as described [32], or indirectly co-cultured by seeding CAFs onto a transwell membrane, and transfected PDAC cells into a 6-well plate, at a 3:1 ratio. Cells were allowed to adhere for 24 h, then placed in an IncuCyte® S3 Live-Cell Analysis System (Essen BioScience) with phase contrast and green fluorescence images taken every 30 min for a further 48 h. The IncuCyte® cell-by-cell analysis software was used to quantify the number of GFP-positive cancer cells. To measure apoptosis, GFP-labelled PDAC cells were transfected with control siRNA or βIII-tubulin siRNA and co-seeded into 6 well plates with CAF cells at a 3:1 ratio of CAF:PDAC cells. At 72 h post transfection, cells were harvested for detection of apoptosis with Annexin V/DAPI staining on a FortessaSORP flow cytometer. Cells were gated for GFP-positive cells to quantify apoptosis in tumour cells only (gating strategy shown in Supplementary Fig. 17B). To observe caspase 8 cleavage in a coculture setting, PDAC cells were transfected with control siRNA or βIII-tubulin siRNA and co-seeded into 8-well chamber slides with CAF cells (1:3 ratio). At 48 h post-transfection, cells were treated with or without 2 μg/mL TNFα neutralising antibody (R&D, cat. MAB210). Cells were fixed in 4% paraformaldehyde 72 h post transfection. Immunofluorescence staining was performed as described above with antibodies for cleaved caspase 8 (rabbit, 1:100; Cell Signaling Technology, cat. #9496), AlexaFluor-488 conjugated cytokeratin (1:100, BioLegend, cat. 628608), and secondary antibody goat anti-rabbit AF647 (1:500, abcam, cat. Ab150079). Stained cells were mounted with Prolong Gold anti-fade mounting media with DAPI and images taken on a Zeiss LSM800 confocal microscope using a 40x/1.3 NA objective. For quantification of caspase 8 cleavage, QuPath v0.3.2 [53] was used to count the percentage of cytokeratin positive PDAC cells that were positive for cleaved caspase 8.

### Subcutaneous mouse PDAC tumour model

Subcutaneous mouse model was approved by the UNSW Animal Ethics committee (ACEC 22/3 A). 2 × 10^6^ MiaPaCa2 + 2 × 10^6^ CAF cells were implanted in 100 μL PBS subcutaneously into the right or left flank of 8-week-old female balb/c nude mice. When tumours reached an average volume of 75 mm^3^, mice were randomised into 4 treatment groups based on tumour volume. Mice were treated with Star 3 nanoparticles (120 μg per mouse) complexed with control siRNA (antisense: 5′-GAACUUCAGGGUCAGCUUGCCG) or βIII-tubulin siRNA and administered intravenously at 2 mg/kg twice weekly for 3.5 weeks following 3 consecutive treatments on days 17, 19, and 20 post-tumour establishment. Blinding was not possible during treatments due to staff availability. Staff performing tumour measurements were blinded to treatments. Mice were treated once weekly with intratumoural PBS (50 μL) or TRAIL (0.5 μg in 50 μL per mouse, Merck-Millipore, cat. GF092). Tumour volume was measured as described [32, 47].

### Patient derived PDAC explant culture

Patient surgical PDAC tumour samples were obtained from Prince of Wales Public and Private Hospitals (Randwick, New South Wales, Australia). All patients provided written informed consent through the Health Precincts Biobank, all work was approved by UNSW Human Ethics (HC180973), and all experiments were performed in accordance with the relevant regulations. PDAC explants were established and cultured for 12 days as described [24]. Control siRNA or βIII-tubulin siRNA (20 µg) complexed Star 3 nanoparticles were added to the medium reservoir as described [24, 32] on days 0, 3, 6, 9. On days 5 and 10, tumour explants were treated with or without 500 ng/mL human recombinant TRAIL. TRAIL concentration was 40-fold lower than the maximum serum concentration of dulanermin (clinical TRAIL drug) measured in clinical pharmacokinetic studies [57]. Explants were treated with 10 μM BrdU substrate (BD Biosciences, catalogue no. 550891) for 24 h prior to fixation (day 12), as described previously [24]. H&E-stained sections of tumour explants are shown in Supplementary Fig. 16.

### Immunohistochemistry of human pancreatic ductal adenocarcinoma tissue sections

Immunohistochemistry on paraformaldehyde-fixed and paraffin-embedded PDAC tumour explant sections was performed as described [24]. Tissue sections were stained with antibodies detailed in Supplementary Table 3. Isotype control antibodies (mouse IgG2A, mouse IgG1A, and rabbit IgG) were used as negative controls (representative images shown in Supplementary Fig. 20). All stained slides were scanned on Vectra Polaris (PerkinElmer) or VS200 (Olympus) (40x objective) slide scanners. Quantification of staining was performed using the positive cell detection function on QuPath.

### Correlation of βIII-tubulin expression in human pancreatic ductal adenocarcinoma samples with overall survival

Staining for βIII-tubulin in human PDAC tissue microarrays (TMA) was performed as described above. PDAC TMAs (International Cancer Genome Consortium Cohort) were obtained through the Australian Pancreatic Cancer Genome Initiative. All work was approved by UNSW Human Ethics (HC180973). All patients provided written informed consent, and patient demographics are included in Supplementary Table 1. Stained TMA slides were scanned on an AperioXT (Leica Biosystems) slide scanner. Staining intensity and percentage of stained cells in tumour and stromal compartments were scored by 3 independent scorers on a 4-point scale (0,1,2,3). The following index was then used to calculate the overall score for tumour and stromal compartments, per scorer, based on the percentage of cells stained: an overall score of 0 if 100% of cells were negative; an overall score of 1 if greater than 50% of cells were scored 0–1; an overall score of 2 if there was a 50:50 ratio of cells with a score of 0–1 to a score of 2–3; or an overall score of 3 if more than 50% of cells were scored as a 2-3. This scoring has been described previously [14]. A consensus score was then determined for each core. For each set of 3 cores per patient, the highest tumour and stroma scores were used. Overall scores of 0-1 were classified as low, and overall scores of 2–3 were classified as high. Scores were then correlated with overall survival using a Kaplan–Meier Survival Curve (see statistical analyses). Patients deceased due to other causes/still alive were censored. Non-PDAC tumours and patients that lacked 3 full cores were excluded (patient cohort details summarised in Supplementary Table 1).

### Validation of βIII-tubulin antibody

The βIII-tubulin antibody used in this study had been previously shown to be specific to βIII-tubulin [12]. Our lab previously demonstrated using western blot that knockdown of βII-tubulin did not affect βIII-tubulin protein levels, indicating that the βIII-tubulin antibody does not bind to βII-tubulin, which is one of the major isotypes expressed in cells. We also showed positive staining of βIII-tubulin in human brain tissue, and negative staining in normal pancreas tissue, consistent with expression in the human protein atlas (Supplementary Fig. 21). Specificity was also shown by demonstrating protein knockdown via western blot using both siRNA and shRNA (Supplementary Fig. 1).

### Statistical analysis

Statistical analyses were performed using GraphPad Prism 9 (GraphPad Software). All data were presented as mean ± standard error of mean (SEM). Sample size was selected based on similar prior studies that demonstrated minimum number required to obtain statistical significance for in vitro and in vivo studies. Patient numbers for the cohort analysis were based on the maximum number of patients provided in the cohort and their power to detect a 32% difference with 80% confidence. All specific replicate numbers and individual replicate data points are provided in figures and figure legends. All in vitro experiments utilizing PDAC cells were technical replicates. All explant experiments, in vivo experiments and in vitro experiments involving CAFs are biological replicates (Independent patient-derived CAFs). For experiments with *n* ≥ 3 independent replicates, two-tailed Student's *t*-test or one-way ANOVA (non-parametric for non-normally distributed data, and parametric for normally distributed data) followed by Bonferroni of Dunn post-hoc test were performed to calculate statistical significance, which was assigned to *p* < 0.05. Comparisons of univariate time to event (survival) were performed using the log-rank test and hazard ratios calculated from the Cox proportional hazards (PH) model. Multivariate associations between variables and time to event were contained from PH regression and survival curves calculated using the method of Kaplan-Meier (KM). Where tumour and stroma scores correlated with outcome, baseline variables associated with predicting scores were examined by multivariate logistic regression. Tumour grade was excluded from multivariate analyses as it did not correlate with overall survival in our cohort due to the low percentage of grade 3–4 tumours (13% of cohort). Survival analyses were performed using Analysis of Censored and Correlated Data (ACCoRD; RRID:SCR_009015) V6.4 Boffin. A *p*-value < 0.05 was considered statistically significant.

### Ethics statement

All mouse work was approved by the UNSW Sydney animal care and ethics committee (approval: ACEC 12/7B; 13/130B; 18/54B). All studies involving the use of human specimens were approved by the UNSW Sydney human ethics committee (approvals: HC14039, HC180973). All patients provided written informed consent.

## Supplementary information

Supplementary methods and full consortium list

Supplementary figures

Supplementary full western blots

Supplementary Movie 1

Supplementary Movie 2

## Data Availability

The data generated in this study are available upon request from the corresponding author and are available within the article and its supplementary data files.
