# Supplementary material for: βIII-tubulin can act as a brake on extrinsic apoptosis in pancreatic cancer
Source: Cell Death Dis. 2026 Apr 24;17(1):547. doi: 10.1038/s41419-026-08657-6 (PMC13243481; doi:10.1038/s41419-026-08657-6)
Supplement: Supplementary file 1 — Supplementary methods and full consortium list [file 41419_2026_8657_MOESM1_ESM.pdf]

Supplementary methods

Drug and inhibitor treatments

Inhibitors of caspase 9 (Z-LEHD-FMK; Cat. 1149-1, BioVision) and caspase 8 (Q-IETD-OPh; Cat. 1176-1, BioVision) were used to treat MiaPaCa2 (20 μM inhibitors) and PANC1 (50 μM inhibitors) cells, 48 hours post-siRNA transfection. Apoptosis was measured 24 hours later. Inhibitors were used at doses previously validated in PDAC cells [52]. Both inhibitors were dissolved in dimethyl sulfoxide (DMSO) and DMSO vehicle was used as the 0 μM control. Inhibitor activity was validated in **Supplementary Figure 19A-B**. For co-treatment with TRAIL, caspase 8/9 inhibitor was added to cells in a 1 hour pre incubation prior to TRAIL addition (10 ng/mL in MiaPaCa2), 48 hours post-transfection.

Recombinant human TRAIL (abcam, cat. Ab9960) and TNFα (abcam, cat. ab9642) was reconstituted in sterile H_2O. FasL (Sigma-Aldrich, cat. SRP3036) was dissolved in sterile 1x PBS containing 0.1% bovine serum albumin. Trimeric TRAIL (LSBio, cat. LS-G3910-10) was reconstituted in 100μL sterile water with 0.1% bovine serum albumin (BSA). DR4 agonist (Creative Biolabs, cat. TAB-H48) and DR5 agonist (Creative Biolabs, cat. TAB-203) were directly diluted in culture medium. To determine the effect of βIII-tubulin silencing on sensitivity to TRAIL/trimeric TRAIL (MiaPaCa2: 5-20 ng/mL; PANC1: 5-20 ng/mL; TKCC5: 2.5 ng/mL; TKCC10: 20 ng/mL; H460: 2.5 ng/mL), DR4/5 agonists (MiaPaCa2: 0.1μg/mL & 1μg/mL), TNFα (MiaPaCa2: 10 ng/mL; PANC1: 10 ng/mL; TKCC5: 20 ng/mL; TKCC10: 20 ng/mL), or FasL (MiaPaCa2: 50 ng/mL; TKCC10: 100 ng/mL), cells were treated 48 hours post-transfection with siRNA. Doses were chosen based on a 50% increase in apoptosis. Cells were harvested for measurement of apoptosis or cell viability 24 hours post-treatment, with the exception of FasL in MiaPaCa2 cells (48h post-treatment).

Immunohistochemistry analysis of mouse orthotopic and sub-cutaneous pancreatic tumour sections

Paraffin-embedded tumour sections were stained with βIII-tubulin antibody (1:50; Biolegend, cat. 801202) as described previously [12,23,32]. Cleaved caspase 8 immunohistochemistry staining was performed using cleaved caspase 8 primary antibody (1:100; Cell Signalling Technology, cat. #9496) and goat anti-rabbit biotinylated secondary (1:200; Vector Laboratories, cat. BA-1000). Briefly, tissue sections were deparaffinised at 60 °C for 30 minutes then rehydrated through consecutive washes in xylene, ethanol, and water. Antigen retrieval was performed by microwaving slides for 4 minutes in 10 mM citrate buffer + 0.05% Tween-20 at pH6.0, followed by a 15-minute incubation at 104 °C. Non-specific peroxidase activity was blocked with 1% hydrogen peroxide + 1% methanol for 10 minutes at room temperature. After blocking in 10% goat serum, tissue samples were stained with cleaved caspase 8 primary antibody diluted 1:100 and incubated overnight at 4 °C. Biotinylated anti-rabbit secondary antibody (1:200) was followed by incubation with Vectastain® ABC kit (Vector laboratories). 3,3' diaminobenxidine (DAB) was used as the substrate, and tissues were counterstained with hematoxylin. All stained tissue sections were scanned on a Vectra Polaris (PerkinElmer) slide scanner using a 40x/0.75 NA objective. Quantification of cleaved caspase 8 staining was performed on QuPath v0.3.2 to count the percent of total cells cleaved caspase 8 positive in whole scanned tumour sections. Isotype control antibodies were used at the same concentration as primary antibodies (Mouse IgG2A for βIII-tubulin stain; Rabbit IgG for cleaved caspase 8 stain; **Supplementary Figure 20**).

Anoikis assays

Anoikis assays were performed as previously described [12]. Briefly, two coats of poly 2-hyroxyethyl methacrylate (Poly-HEMA; 12 mg/mL, dissolved in 95% ethanol) were added to 6-well tissue culture plates and left to dry overnight at room temperature. MiaPaCa2 cells were

transfected with control or βIII-tubulin siRNA as described in main manuscript. Twenty four hours post-transfection cells were re-seeded into the Poly-HEMA coated plates. Forty eight hours post-transfection, cells were cultured with or without TRAIL (10 ng/mL) for a further 9 hours and apoptosis measured by Annexin V staining and flow cytometry.

DR4 / DR5 TRAIL receptor immunofluorescence

MiaPaCa2 and PANC1 cells were re-seeded into 8-well chamber slides (Ibidi, cat. 80826) at 10,000 cells/well, 24 hours post transfection. 72 hours post-transfection, cells were treated with TRAIL (250ng/mL) for 2 hours, then fixed in 4% paraformaldehyde for 10 minutes. Immunofluorescence staining was performed as described [12], using primary antibodies for DR5 (1:100; Cell Signalling Technologies, cat. 8074) and α-tubulin (1:500; Sigma-Aldrich, cat. T9026). Secondary antibodies used were goat anti-rabbit AF488 (1:500; Molecular Probes, cat. A-11008) and goat anti-mouse AF647 (1:500; Abcam, cat. ab150115). Stained cells were mounted with Prolong Gold anti-fade mounting media with DAPI. Images were taken on a Zeiss LSM800 confocal microscope. For quantification of DR5 cluster size, z-stack images (7 slices at 3 μm) were obtained using a 40x/1.3 NA objective to capture an average of 20 cells per field of view, and 3-5 representative images were captured for each sample. Maximum intensity projections of the z-stack images were processed using Zeiss Zen Black 2.3 software, and DR5 cluster size was measured using the Analyse Particle function on ImageJ v1.52a (National Institutes of Health, Bethesda, Maryland, USA). DR5 cluster size was normalised to cell number by counting the number of DAPI positive cells. MiaPaCa2 cells were also stained for DR4 (1:100; Cell Signalling Technology, cat. 42533) as described above. DR4 mean fluorescence intensity per cell was quantified using ImageJ v1.52a (National Institutes of Health, Bethesda, Maryland, USA).

Live imaging of GFP-tagged death receptor 5 (DR5) in pancreatic cancer cells

MiaPaCa2 cells were lentivirally transduced with GFP-tagged DR5 (Origene, Cat. RC201588L4V) and selected in 1 μg/mL puromycin for 2 weeks, then GFP-positive cells sorted on a BD FACS Aria II. Sorted cells were transfected with ns-siRNA or βIII-tubulin siRNA and then re-seeded into 8-well Ibidi chamber slides (#1.5, 0.170 mm thickness, Ibidi, cat. 80826). At 48 hours post-transfection, cells were imaged on a Zeiss Elyra 7 Lattice SIM^2 microscope using a 63x/1.46 objective with total internal reflection (TIRF) imaging to visualise the thin layer (<150 nm) containing the cell membrane (TIRF mirror angle set to 66.25°). 488 nm laser line was used to excite GFP and BP 495-550 emission filter for collection. To observe whether DR5 clustering precedes cell death, cells were imaged for 50 minutes every 25 seconds, while being maintained in a humidified chamber at 37 °C and 5% CO_2. To analyse the dynamics and diffusion of DR5-GFP, cells were imaged with 50 ms exposure on PCO edge sCMOS (pixel size 0.097 μm), using the same TIRF settings, and 1000 frames were acquired per field of view. DR5-GFP diffusion was quantified using k-space image correlation spectroscopy (kICS), as previously described [54-56]. Briefly, image time series were loaded in a custom-built script in MATLAB (MathWorks, Natick, MA). First, images were corrected for potential spatial drift using *imregtform* function in MATLAB. Next, pixels outside of the cellular areas were padded with the mean intensity of pixels within cells, as this ensures that they do not contribute to the kICS correlation function. Prior to application of kICS correlation function (CF) calculation, the padded image series were spatially Hann windowed, to remove the high spatial frequencies 'leakage' in kICS CF, coming from the image cell edges. kICS CF was calculated as previously described [54-56] and azimuthally averaged at every temporal lag. The resulting kICS Cf was fitted with two dynamic components as detailed previously [54-56] and temporally (tau lag) varying amplitudes and decays of dynamic components assessed to extract the diffusion coefficients at micro and macro spatial scales. The effective diffusion on largest

spatial scale is extracted from long tau slope of macro component and represents the diffusion of DR5-GFP over whole cell surface area.

Refer to main manuscript for reference list.

Australian Pancreatic Cancer Genome Initiative Consortium

Garvan Institute of Medical Research Amber L. Johns[9], Anthony J Gill[9,5], Lorraine A. Chantrill[9,41], Paul Timpson[9,] Angela Chou[9,5], Marina Pajic[9], Tanya Dwarte[9], David Herrmann[9], Claire Vennin[9], Thomas R Cox[9], Brooke Pereira[9], Shona Ritchie[9], Daniel A Reed[9], Cecilia R Chambers[9], Xanthe Metcalf[9], Max Nobis[9], Gloria Jeong[9], Ruth J. Lyons[9]. **QIMR Berghofer Medical Research Institute** Nicola Waddell[22], John V. Pearson[22], Ann-Marie Patch[22], Katia Nones[22], Felicity Newell[22], Pamela Mukhopadhyay[22], Venkateswar Addala[22], Stephen Kazakoff[22], Oliver Holmes[22], Conrad Leonard[22], Scott Wood[22]. **University of Melbourne, Centre for Cancer Research** Sean M. Grimmond[23], Oliver Hofmann[23]. **Royal North Shore Hospital** Jaswinder S. Samra[24], Nick Pavlakis[24,] Jennifer Arena[24], Hilda A. High[24]. **Bankstown Hospital** Ray Asghari[25], Neil D. Merrett[25], Amitabha Das[25]. **Liverpool Hospital** Peter H. Cosman[26], Kasim Ismail[26]. **St Vincent's Hospital** Alina Stoita[27], David Williams[27], Allan Spigellman[27]**. Westmead Hospital** Duncan McLeod[28], Judy Kirk[28]. **Royal Prince Alfred Hospital, Chris O'Brien Lifehouse** James G. Kench[29], Peter Grimison[29], Charbel Sandroussi[29], Annabel Goodwin[26,29]. **Prince of Wales Hospital** R. Scott Mead[9,30], Katherine Tucker[30], Lesley Andrews[30]. **Fiona Stanley Hospital** Michael Texler[31], Cindy Forrest[31], Mo Ballal[31,32], David Fletcher[31]**. St John of God Healthcare** Maria Beilin[32], Kynan Feeney[32] Krishna Epari[32] Sanjay Mukhedkar[32]. **Epworth HealthCare** Nikolajs Zeps[42]. **Royal Adelaide Hospital** Nan Q Nguyen[33], Andrew R. Ruszkiewicz[33], Chris Worthley[33]. **Flinders Medical Centre** John Chen[34], Mark E. Brooke-Smith[34], Virginia Papangelis[34]. **Envoi Pathology** Andrew D. Clouston[35]. **Princess Alexandra Hospital** Andrew P. Barbour[36], Thomas J. O'Rourke[36], Jonathan W. Fawcett[36], Kellee Slater[36], Michael Hatzifotis[36], Peter Hodgkinson[36]. **Austin Hospital** Mehrdad Nikfarjam[37]. **Johns Hopkins Medical Institutes** James R. Eshleman[38], Ralph H. Hruban[38], Christopher L. Wolfgang[38]. **ARC-Net Centre for Applied Research on Cancer** Aldo Scarpa[39], Rita T. Lawlor[39], Vincenzo Corbo[39], Claudio Bassi[39]. **University of Glasgow** Andrew V Biankin[40], Nigel B. Jamieson[40], David K. Chang[9, 40], Stephan B. Dreyer[40].

[9]The Kinghorn Cancer Centre, Garvan Institute of Medical Research, 370 Victoria Street, Darlinghurst, Sydney, New South Wales 2010, Australia.

[22]QIMR Berghofer Medical Research Institute, 300 Herston Rd, Herston, Queensland 4006, Australia.

[23]University of Melbourne, Centre for Cancer Research, Victorian Comprehensive Cancer Centre, 305 Grattan Street, Melbourne, Victoria 3000, Australia.

[24]Royal North Shore Hospital, Westbourne Street, St Leonards, New South Wales 2065, Australia.
[25]Bankstown Hospital, Eldridge Road, Bankstown, New South Wales 2200, Australia.
[26]Liverpool Hospital, Elizabeth Street, Liverpool, New South Wales 2170, Australia.
[27] St Vincent's Hospital, 390 Victoria Street, Darlinghurst, New South Wales, 2010 Australia.
[28]Westmead Hospital, Hawkesbury and Darcy Roads, Westmead, New South Wales 2145, Australia.
[29]Royal Prince Alfred Hospital, Missenden Road, Camperdown, New South Wales 2050, Australia.
[30]Prince of Wales Hospital, Barker Street, Randwick, New South Wales 2031, Australia.
[31]Fremantle Hospital, Alma Street, Fremantle, Western Australia 6959, Australia.
[32]St John of God Healthcare, 12 Salvado Road, Subiaco, Western Australia 6008, Australia.
[33]Royal Adelaide Hospital, North Terrace, Adelaide, South Australia 5000, Australia.
[34]Flinders Medical Centre, Flinders Drive, Bedford Park, South Australia 5042, Australia.
[35]Envoi Pathology, 1/49 Butterfield Street, Herston, Queensland 4006, Australia.
[36]Princess Alexandra Hospital, 199 Ipswich Rd, Woolloongabba QLD 4102
[37]Austin Hospital, 145 Studley Road, Heidelberg, Victoria 3084, Australia.
[38]Johns Hopkins Medical Institute, 600 North Wolfe Street, Baltimore, Maryland 21287, USA.
[39]ARC-NET Center for Applied Research on Cancer, University of Verona, Via dell'Artigliere, 19 37129 Verona, Province of Verona, Italy.
[40]Wolfson Wohl Cancer Research Centre, Institute of Cancer Sciences, University of Glasgow, Garscube Estate, Switchback Road, Bearsden, Glasgow, Scotland G61 1BD, United Kingdom.
[41]Wollongong Hospital, Illawarra and Shoalhaven Local Health District, Loftus Street, Wollongong NSW 2500.
[42]Epworth HealthCare, 89 Bridge Rd, Richmond VIC 3121, Australia
