# Supplementary material for: βIII-tubulin can act as a brake on extrinsic apoptosis in pancreatic cancer
Source: Cell Death Dis. 2026 Apr 24;17(1):547. doi: 10.1038/s41419-026-08657-6 (PMC13243481; doi:10.1038/s41419-026-08657-6)
Supplement: Supplementary file 2 — Supplementary figures [file 41419_2026_8657_MOESM2_ESM.pdf]

Age at diagnosis	Number of patients
≥50	146
<50	9
**Gender**	
Male	78
Female	77
**Ethnicity**	
Asian	10
Asian, White/Caucasian	1
Black/African	2
Pacific Islander	1
White/Caucasian	141
**Smoker**	
Ever	80
Never	72
Not reported	3
**Alcohol consumption**	
Ever	88
Never	63
Not reported	4
**Margin Status**	
R0	108
R1	42
R2	3
RX	2
**Macroscopic tumour location**	
Ampulla	1
Body	10
Head	116
Head (Uncinate)	8
Tail	17
Not reported	3

Overall Stage	Number of patients
IA	2
IB	4
IIA	31
IIB	109
III	1
IV	6
Not reported	2
**TNM Staging**	
T1	2
T2	7
T3	142
T4	1
TX	3
N0	37
N1	78
N1a	4
N1b	32
NX	4
M0	3
M1	6
MX	144
**Perineural invasion**	
Yes	127
No	22
Not reported	6
**Vascular invasion**	
Yes	94
No	55
Not reported	6
**Recurrence at liver**	
Yes	50
No	58
No recurrence	47

Supplementary Table 1. Australian Pancreatic Cancer Genome Initiative (APGI) International Cancer Genome Cohort (ICGC) patient characteristics for βIII-tubulin survival analyses. Human PDAC tissue microarrays were obtained through the APGI. Patient cohort characteristics are described above. TNM staging refers to tumour size (T), lymph node involvement (N), and metastasis (M).

Univariate analysis of parameters used in multivariate analysis				
**Parameter**	**HR**	**95%**	**CI**	**p-value**
βIII-Tubulin Tumour score	1.526	1.052	2.206	0.026
βIII-Tubulin Stroma score	1.766	1.227	2.541	0.002
Gender	0.651	0.454	0.934	0.020
Age at Diagnosis (years)	0.975	0.453	2.102	0.949
Smoker	1.292	0.896	1.863	0.170
Alcohol consumption	1.129	0.779	1.636	0.520
Margin Status	1.624	1.106	2.385	0.013
Lymph Nodes Involved	1.453	0.928	2.274	0.102
Perineural Invasion	1.727	0.986	3.025	0.056
Vascular Invasion	1.779	1.193	2.655	0.005
Overall Stage AJCC	1.691	1.072	2.665	0.024
Macroscopic Tumour Location	1.103	0.598	2.035	0.754
**Multivariate results (best subsets)**	**HR**	**95%**	**CI**	**p-value**
βIII-Tubulin Stroma score	2.163	1.208	3.874	0.009
Margin Status	2.043	1.345	3.103	0.001

Supplementary Table 2. βIII-tubulin multivariate survival analysis parameters. Comparisons of univariate time to event (survival) were performed using the log-rank test and hazard ratios (HR) and confidence intervals (CI) calculated from the Cox proportional hazards (PH) model. Multivariate associations between variables and time to event were contained from PH regression and survival curves calculated using the method of Kaplan-Meier (KM). Where tumour and stroma scores correlated with outcome, baseline variables associated with predicting scores were examined by multivariate logistic regression. A p-value ≤0.05 was considered statistically significant.

siRNA	Source	Catalog number	
ON-TARGET*plus* Non-Targeting Control Pool siRNA	Dharmacon	D-001810-10-20	
ON-TARGET*plus* βIII-tubulin SMARTpool siRNA	Dharmacon	L-020099-00	
ON-TARGET*plus* βII-tubulin SMARTpool siRNA	Dharmacon	L-008260-00	
Single sequence βIII-tubulin siRNA (in vitro validation)	Qiagen	1027418	
**qPCR primers**	**Source**	**Catalog number**	
βIII-tubulin	Geneworks	730175-6	
18S	Qiagen	QT00199367	
**Antibody**	**Source**	**Catalog number**	**Dilution**
βIII-tubulin	BioLegend	801202	1:1000 (Western blot); 1:50 (IHC)
GAPDH	abcam	Ab8245	1:50,000
DR5	Cell Signaling Technology	8074	1:1000 (Western blot); 1:100 (Immunofluorescence)
DR4	Cell Signaling Technology	42533	1:1000
Cleaved Caspase 8	Cell Signaling Technology	9496S	1:1000 (Western blot); 1:100 (IHC)
Cleaved Caspase 3	Cell Signaling Technology	9664S	1:1000
Cleaved PARP	Cell Signaling Technology	9541S	1:1000
α-tubulin	Sigma-Aldrich	T9026	1:500 (immunofluorescence)
BrdU	DAKO	M0744	1:50 (IHC)
Cytokeratin	DAKO	M3515	1:100 (IHC)
α-smooth muscle actin	Sigma	A5228	1:1000 (IHC)
Ki67	ThermoFisher	RM9106	1:50 (IHC)
Mouse IgG-HRP (Western blot secondary)	DAKO	P0447	1:2000 (Western blot)
Rabbit IgG-HRP (Western blot secondary)	DAKO	P0448	1:2000 (Western blot)
Rabbit IgG-HRP (IHC secondary)	Vector Laboratories	BA-1000	1:200
rabbit-AF488 (immunofluorescence secondary)	Molecular Probes	A-11008	1:500 (immunofluorescence)
Mouse-AF647 (immunofluorescence secondary)	Abcam	ab150115	1:500 (immunofluorescence)
Mouse IgG1 isotype control	DAKO	X0931	As per matched target antibody concentration
Mouse IgG2A isotype control	ThermoFisher	14472482	As per matched target antibody concentration
Rabbit IgG isotype control	Cell Signaling technologies	CST3900S	As per matched target antibody concentration

Supplementary Table 3. siRNA, primer and antibody details.

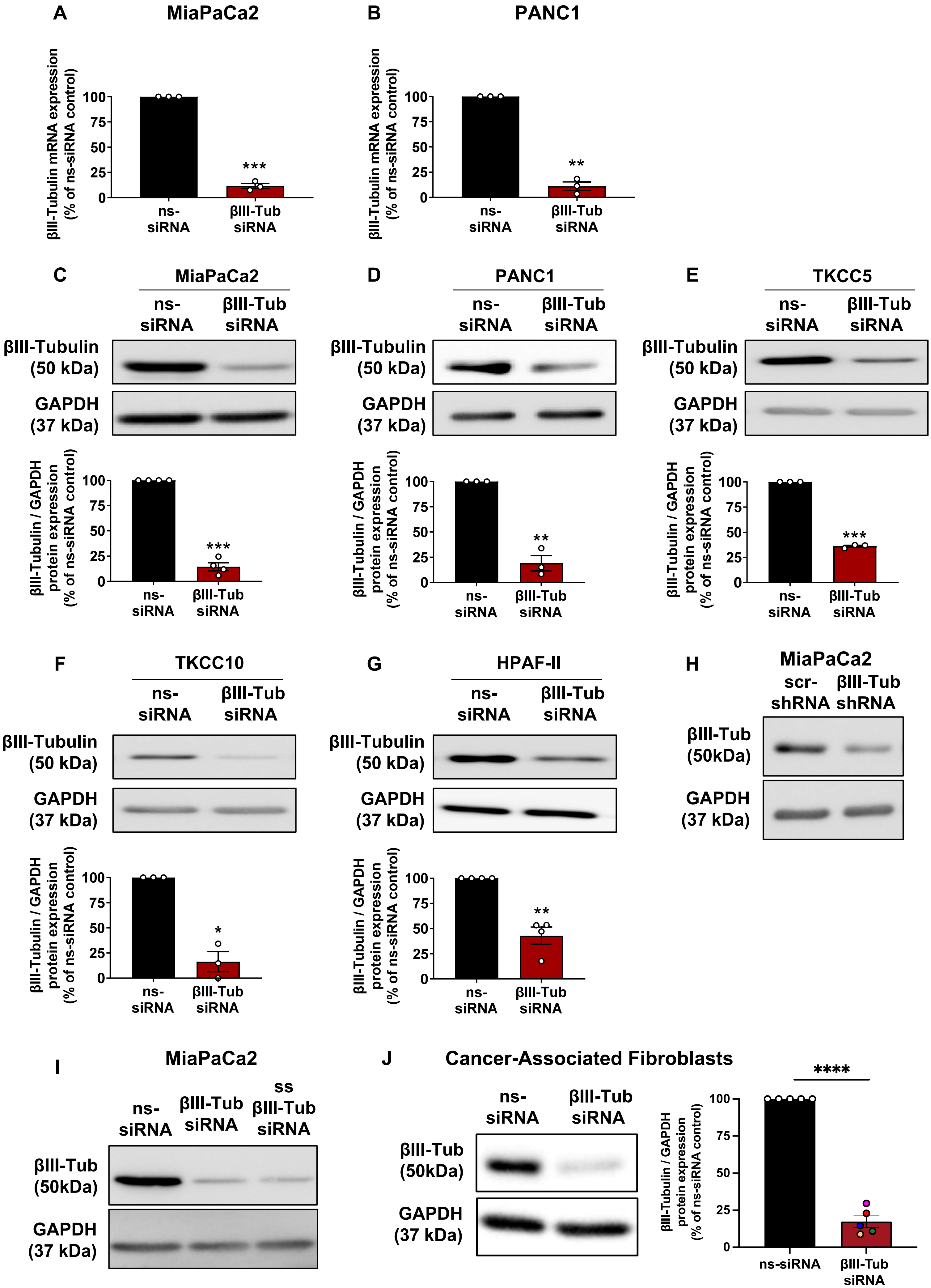
A
MiaPaCa2
B
PANC1
βIII-Tubulin mRNA expression
(% of ns-siRNA control)
100
75
50
25
0
***
**
ns-
siRNA
βIII-Tub
siRNA
C
MiaPaCa2
D
PANC1
E
TKCC5
ns-
siRNA
βIII-Tub
siRNA
βIII-Tubulin
(50 kDa)
GAPDH
(37 kDa)
βIII-Tubulin / GAPDH
protein expression
(% of ns-siRNA control)
*
F
TKCC10
G
HPAF-II
H
MiaPaCa2
scr-
shRNA
βIII-Tub
shRNA
βIII-Tub
(50kDa)
I
MiaPaCa2
ss
βIII-Tub
siRNA
J
Cancer-Associated Fibroblasts
****
ns-siRNA

Supplementary Figure 1. RNA interference drugs (siRNA, shRNA) silence βIII-tubulin expression in PDAC cells and CAFs in vitro. (A-B) βIII-tubulin (βIII-Tub) mRNA expression in **(A)** MiaPaCa2 (n=3 independent experiments) and **(B)** PANC1 cells (n=3 independent experiments) was significantly reduced 72 hours post-transfection with βIII-tubulin siRNA, compared to non-silencing controls, as assessed by quantitative real-time PCR. Samples were standardised to 18S RNA. **(C-I)** Western blot analysis (representative blots shown) with densitometry graphs below showing reduced βIII-tubulin protein levels, 72 hours following transfection with βIII-tubulin siRNA in MiaPaCa2 (n=4) **(C)**, PANC1 (n=3) **(D)**, TKCC5 (n=3) **(E)**, TKCC10 (n=3) **(F)**, and HPAF-II (n=4) **(G)** cells. Densitometry analysis (graphs) was performed by measuring βIII-tubulin protein expression normalised to GAPDH expression. Bars represent mean of n≥3 independent experiments (individual data points shown from independent experiments) ± standard error of mean. Asterisks indicate significance as assessed by two-tailed paired t-tests comparing mean of βIII-Tub siRNA to mean of ns-siRNA (*p≤0.05, **p≤0.01, ***p≤0.001). **(H)** MiaPaCa2 cells with stable expression of βIII-tubulin (βIII-tub) shRNA demonstrated potent knockdown of βIII-tubulin protein (western blot). **(I)** Knockdown of βIII-tubulin using an independent single-sequence (ss) siRNA (Qiagen) demonstrated potent protein silencing comparable to knockdown with SMARTpool siRNA (Dharmacon). **(J)** Western blot analysis (representative blots shown) with densitometry graph showing reduced βIII-tubulin protein levels, 72 hours following transfection with βIII-tubulin siRNA in cancer associated fibroblasts (CAFs). Colours indicate independent patient-derived CAFs. Asterisks indicate significance as assessed by two-tailed paired t-tests comparing mean of βIII-Tub siRNA to mean of ns-siRNA (****p≤0.0001).

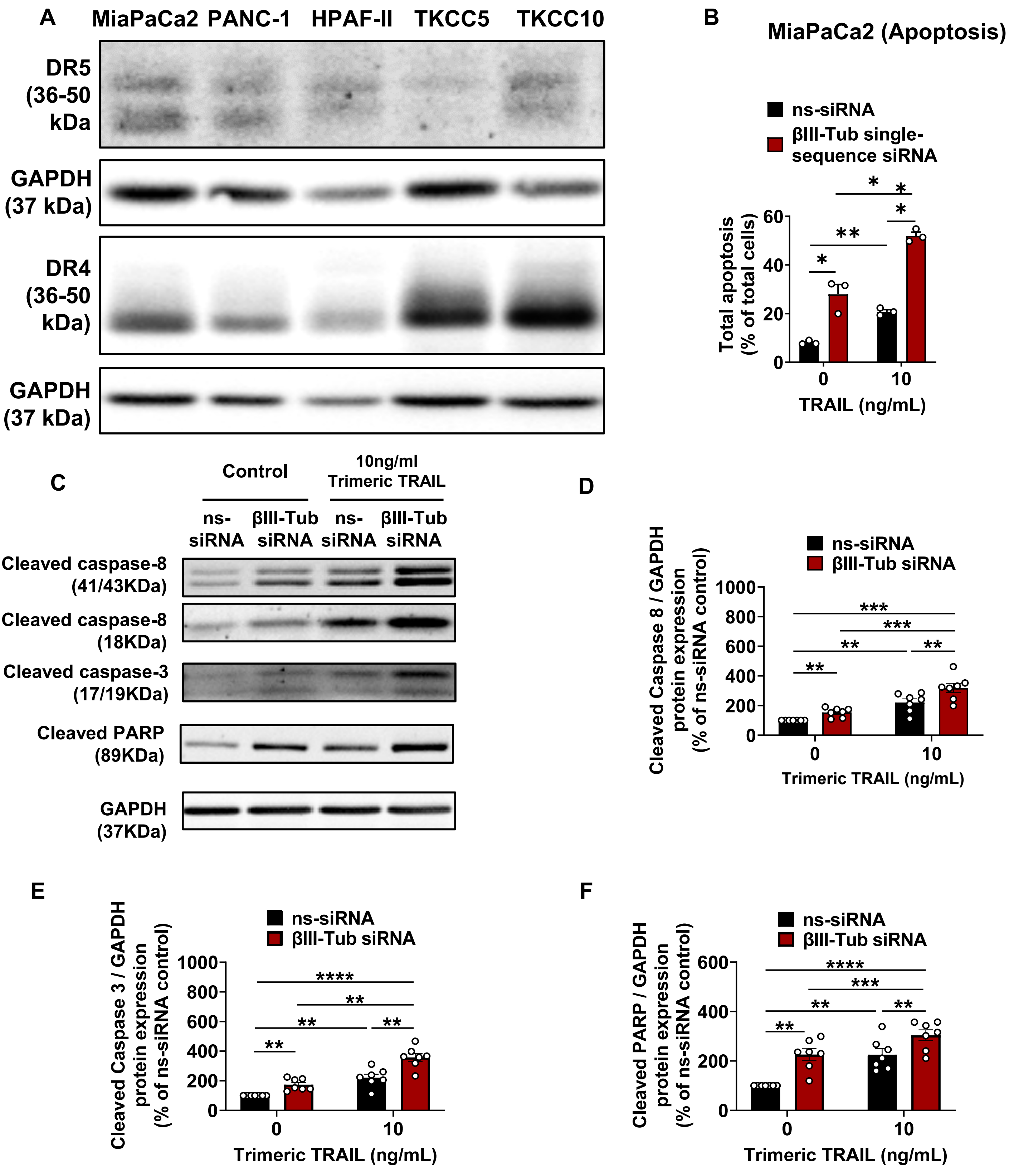

A
MiaPaCa2 PANC-1 HPAF-II TKCC5 TKCC10
DR5 (36-50 kDa
GAPDH (37 kDa)
DR4 (36-50 kDa)
GAPDH (37 kDa)
B
MiaPaCa2 (Apoptosis)
ns-siRNA
βIII-Tub single-sequence siRNA
Total apoptosis (% of total cells)
TRAIL (ng/mL)
C
Control
10ng/ml Trimeric TRAIL
ns-siRNA βIII-Tub siRNA ns-siRNA βIII-Tub siRNA
Cleaved caspase-8 (41/43KDa)
Cleaved caspase-8 (18KDa)
Cleaved caspase-3 (17/19KDa)
Cleaved PARP (89KDa)
GAPDH (37KDa)
D
Cleaved Caspase 8 / GAPDH protein expression (% of ns-siRNA control)
ns-siRNA
βIII-Tub siRNA
Trimeric TRAIL (ng/mL)
E
Cleaved Caspase 3 / GAPDH protein expression (% of ns-siRNA control)
ns-siRNA
βIII-Tub siRNA
Trimeric TRAIL (ng/mL)
F
Cleaved PARP / GAPDH protein expression (% of ns-siRNA control)
ns-siRNA
βIII-Tub siRNA
Trimeric TRAIL (ng/mL)

Supplementary Figure 2: βIII-tubulin knockdown in PDAC cells increased sensitivity to TRAIL. **(A)** A western blot was performed to compare the relative expression of TRAIL receptors DR4 and DR5 in PDAC cell lines used in this study. Protein was loaded onto two separate gels to allow imaging of both DR4 and DR5. Membranes were then re-probed for GAPDH as a loading control. **(B)** Knockdown of βIII-tubulin using single sequence siRNA in MiaPaCa2 cells (n=3) increased apoptosis in the presence of TRAIL (10 ng/mL). Apoptosis was assessed by flow cytometry for Annexin V/DAPI 72 hours post transfection. **(C-F)** Representative western blot **(C)** and densitometry analysis showing cleaved caspase-8 **(D)**, cleaved caspase-3 **(E)** and cleaved PARP **(F)** protein expression for MiaPaCa2 cells transfected with non-silencing (ns) or βIII-tubulin (βIII-Tub) siRNA and treated ± TRAIL (10ng/ml). Bars represent mean of n≥3 independent experiments (individual data points shown from independent experiments) ± standard error of mean. Asterisks indicate significance as assessed by One-Way ANOVA (*p≤0.05, **p≤0.01, ***p≤0.001, ****p≤0.0001).

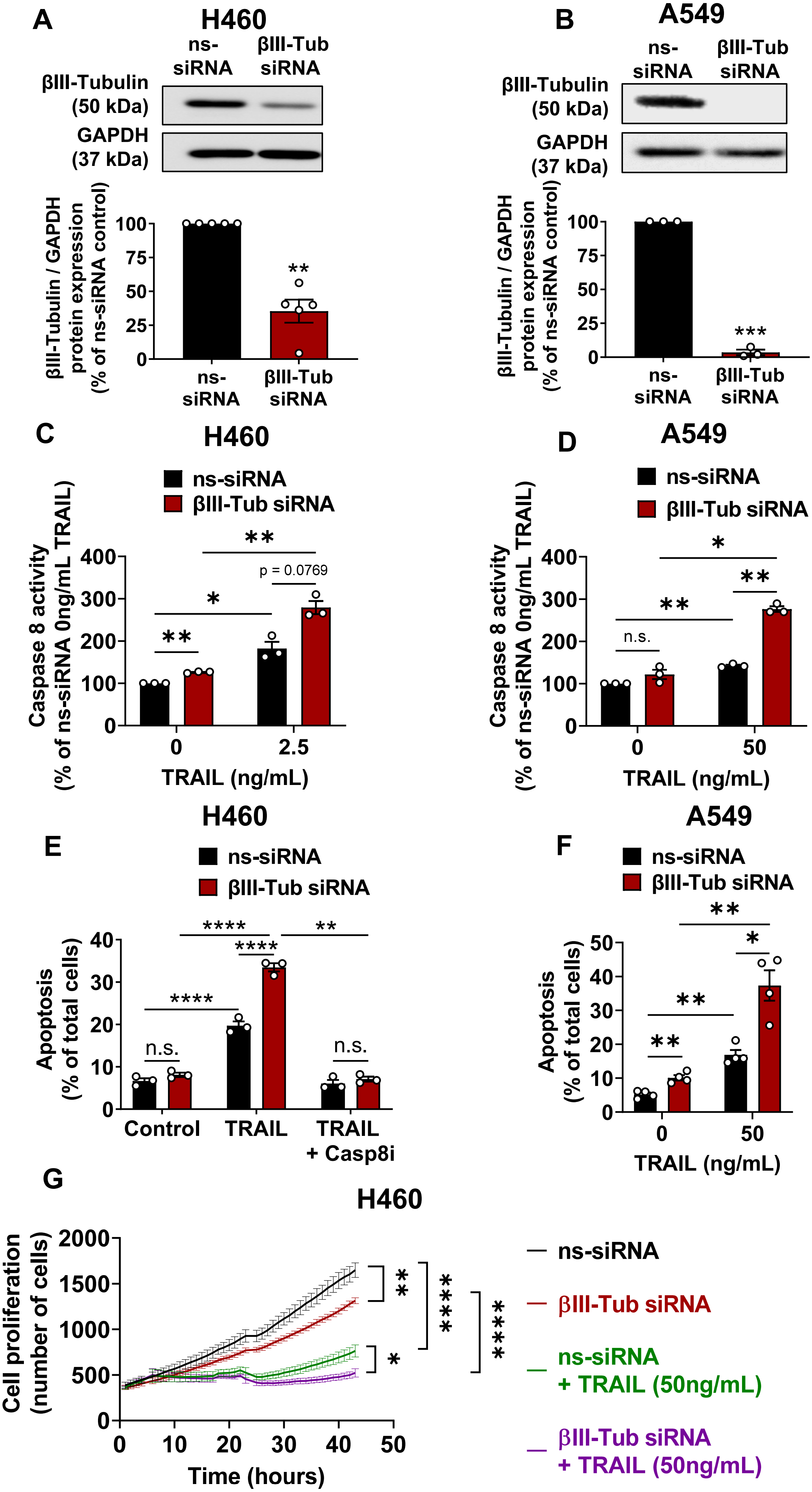

A
H460
ns-siRNA
βIII-Tub siRNA
βIII-Tubulin (50 kDa)
GAPDH (37 kDa)
βIII-Tubulin / GAPDH protein expression (% of ns-siRNA control)
**
B
A549
***
C
H460
ns-siRNA
βIII-Tub siRNA
Caspase 8 activity (% of ns-siRNA 0ng/mL TRAIL)
p = 0.0769
TRAIL (ng/mL)
D
A549
n.s.
E
H460
Apoptosis (% of total cells)
Control
TRAIL
TRAIL + Casp8i
****
F
A549
G
H460
Cell proliferation (number of cells)
Time (hours)
ns-siRNA
βIII-Tub siRNA
ns-siRNA + TRAIL (50ng/mL)
βIII-Tub siRNA + TRAIL (50ng/mL)

Supplementary Figure 3. βIII-tubulin knockdown induced extrinsic apoptosis and decreased cell proliferation in human lung cancer cells treated with TRAIL. (A-B) Western blot analysis (representative blots shown) with densitometry graphs below showing reduced βIII-tubulin protein levels, 72 hours following transfection with non-silencing (ns) or βIII-tubulin (βIII-Tub) siRNA in H460 (n=5) **(A)**, and A549 (n=3) **(B)** cells. Densitometry analysis (graphs) was performed by measuring βIII-tubulin protein expression normalised to GAPDH expression. Bars represent mean of n≥3 independent experiments (individual data points shown from independent experiments) ± standard error of mean. Asterisks indicate significance as assessed by two-tailed paired t-tests comparing mean of βIII-Tub siRNA to mean of ns-siRNA (*p≤0.05, **p≤0.01, ***p≤0.001). **(C-D)** Caspase 8 (n=3-5) activities in (C) H460 and (D) A549 cells measured using CaspaseGlo assay, 72 hours post-transfection with ns-siRNA or βIII-Tub-siRNA. Activity normalised to cell numbers. **(E-F)** Apoptosis measured using flow cytometry for Annexin V/DAPI in NSCLC cells transfected with non-silencing (ns) or βIII-tubulin (βIII-Tub) siRNA and treated ± TRAIL, in H460 (n=4) **(E)**, and A549 (n=6) **(F)** cells. **(G)** IncuCyte® S3 live-cell analysis (proliferation) of H460 cells (n=4) with βIII-tubulin knockdown ± TRAIL. Bars/lines represent mean of n≥3 independent experiments (individual data points shown from independent experiments) ± standard error of mean. Asterisks indicate significance as assessed by student t-test (A-B) or one-Way ANOVA (C-G) (*p≤0.05, ** p≤0.01, **** p≤0.0001, n.s.; non-significant).

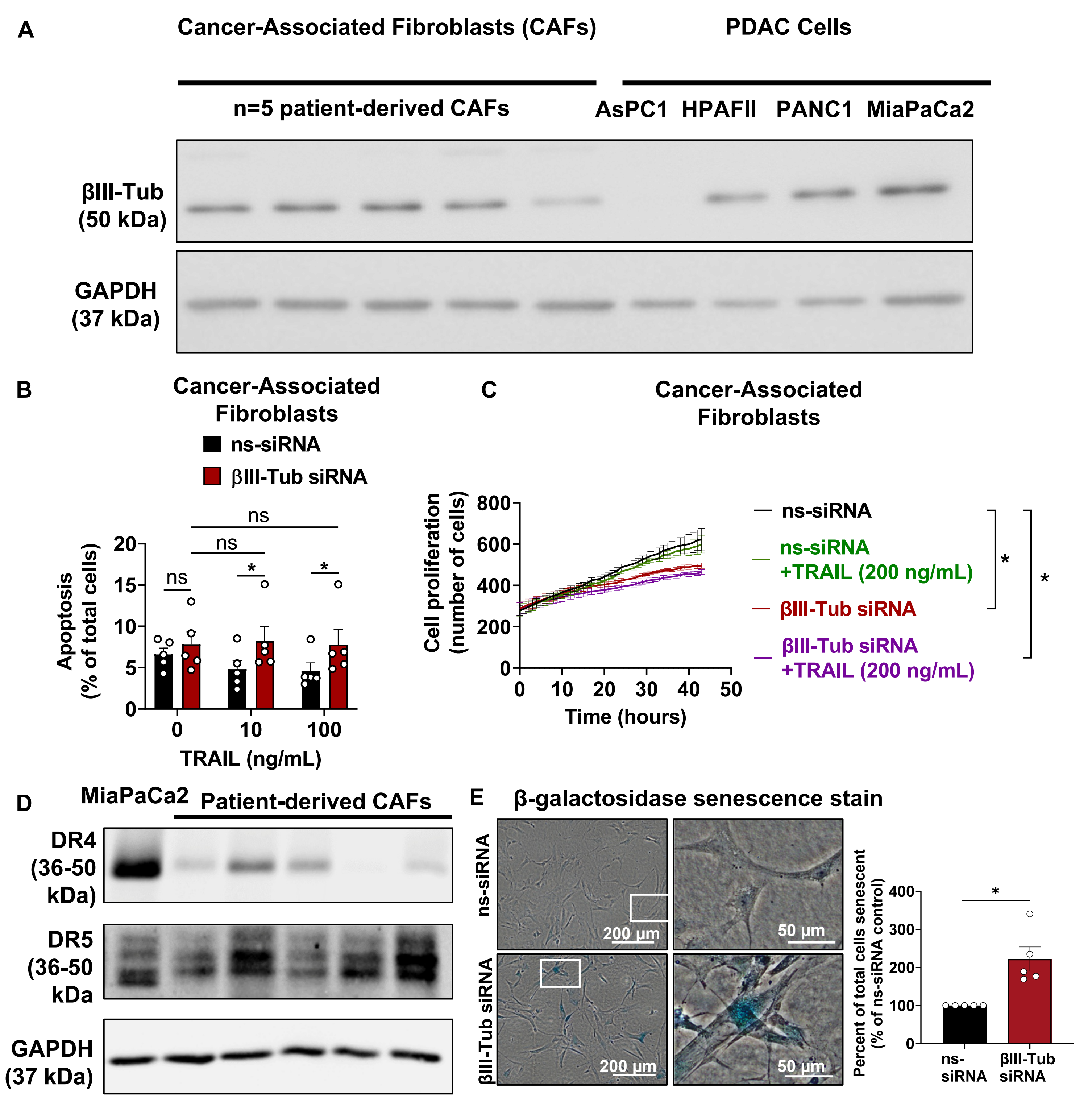

Supplementary Figure 4: βIII-Tubulin silencing in patient-derived PDAC CAF cells had no effect on TRAIL sensitivity and increased senescence. (A) A western blot was performed to compare the relative expression of βIII-tubulin in 5 patient derived CAF lines with 4 commercial PDAC cell lines. Results show comparable expression of βIII-tubulin in CAF and PDAC cells. GAPDH was used as a loading control. **(B)** PDAC CAFs from n=5 patients were treated with TRAIL 48 hours post-transfection with ns-siRNA or βIII-tubulin siRNA. Apoptosis was measured via flow cytometry for annexin V/DAPI at 72 hours post-transfection. **(C)** CAF cell proliferation was reduced with βIII-tubulin knockdown, but there was no further reduction in cell proliferation with the addition of TRAIL. Cells were treated with TRAIL (200 ng/mL) 24 hours post-transfection, and images taken every hour on the IncuCyte S3 (n=4). **(D)** Western blot in MiaPaCa2 cells and CAFs from n=4 PDAC patients revealed varying expression levels of DR4 and DR5. Protein was loaded onto two separate gels to allow imaging of both DR4 and DR5. Membranes were then re-probed for GAPDH as a loading control. **(E)** βIII-tubulin knockdown in PDAC CAFs from 5 patients increased cellular senescence. 72 hours post-transfection with ns-siRNA or βIII-tubulin siRNA, cells were stained overnight using a β-galactosidase kit staining senescent cells in blue. Senescent CAFs were quantified by manual counting of positive cells. Bars represent mean of n≥3 independent experiments (individual data points shown from independent experiments) ± standard error of mean. Asterisks indicate significance as assessed by One-Way ANOVA (*p≤0.05, n.s.; non-significant).

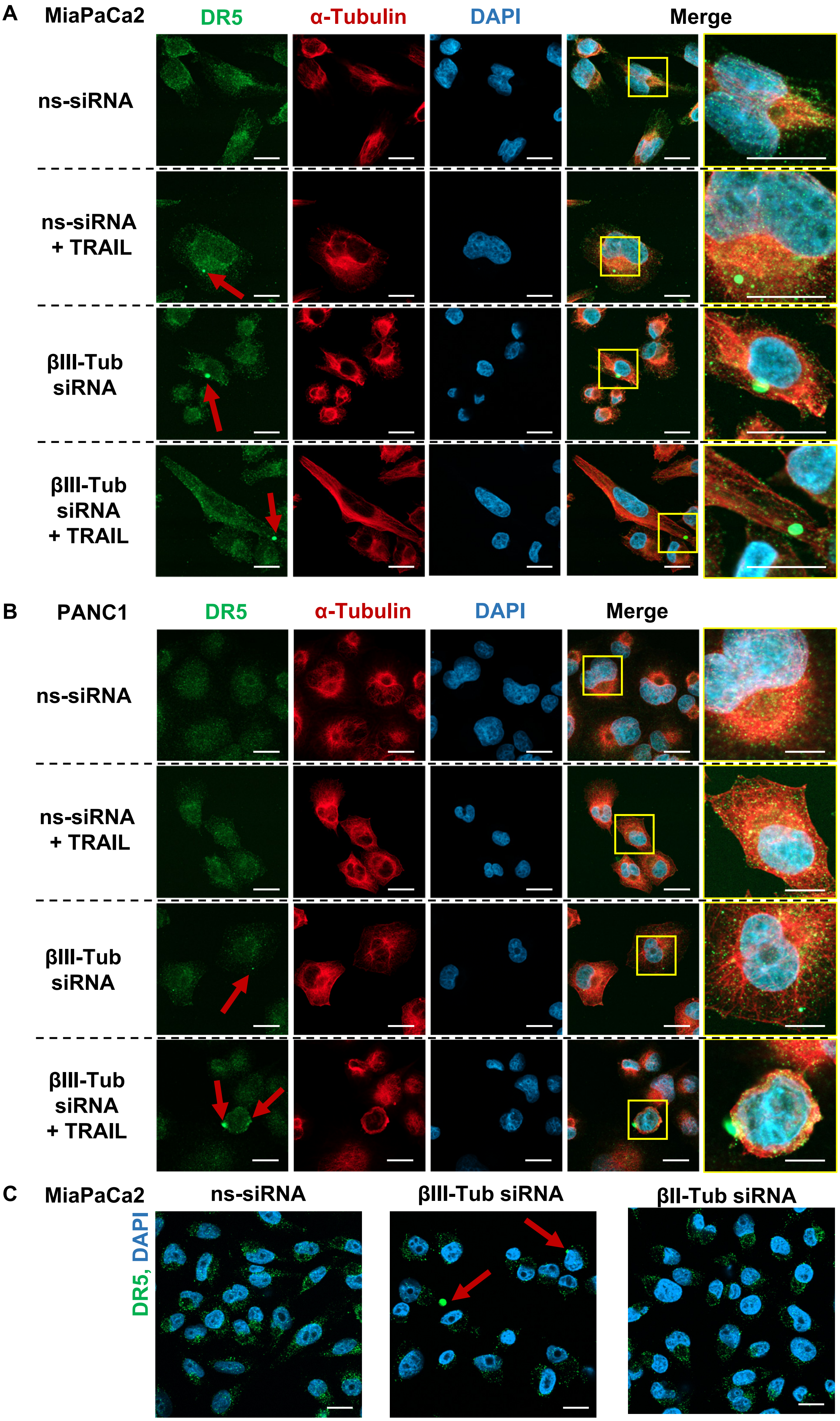

A
MiaPaCa2
DR5
α-Tubulin
DAPI
Merge
ns-siRNA
ns-siRNA + TRAIL
βIII-Tub siRNA
βIII-Tub siRNA + TRAIL
B
PANC1
DR5
α-Tubulin
DAPI
Merge
ns-siRNA
ns-siRNA + TRAIL
βIII-Tub siRNA
βIII-Tub siRNA + TRAIL
C
MiaPaCa2
ns-siRNA
βIII-Tub siRNA
βII-Tub siRNA
DR5, DAPI

Supplementary Figure 5: βIII-Tubulin silencing in PDAC cells triggered DR5 clustering. (A-B) Immunofluorescence staining for DR5 and α-tubulin revealed that βIII-tubulin (βIII-Tub) knockdown triggered the formation of large membrane clusters of DR5 (red arrows) in MiaPaCa2 (representative of n=4 independent experiments) **(A)** and PANC1 (representative of n=4 independent experiments) **(B)** cells. Cells were fixed and stained 72 hours post transfection. MiaPaCa2 merged immunofluorescence images are also shown in **Figure 4A** but are shown here as well with each individual fluorescence channel as well. **(C)** βII-tubulin knockdown in MiaPaCa2 cells did not trigger DR5 clustering compared to βIII-tubulin knockdown. Cells were fixed and stained 72 hours post transfection. All scale bars represent 20 μm.

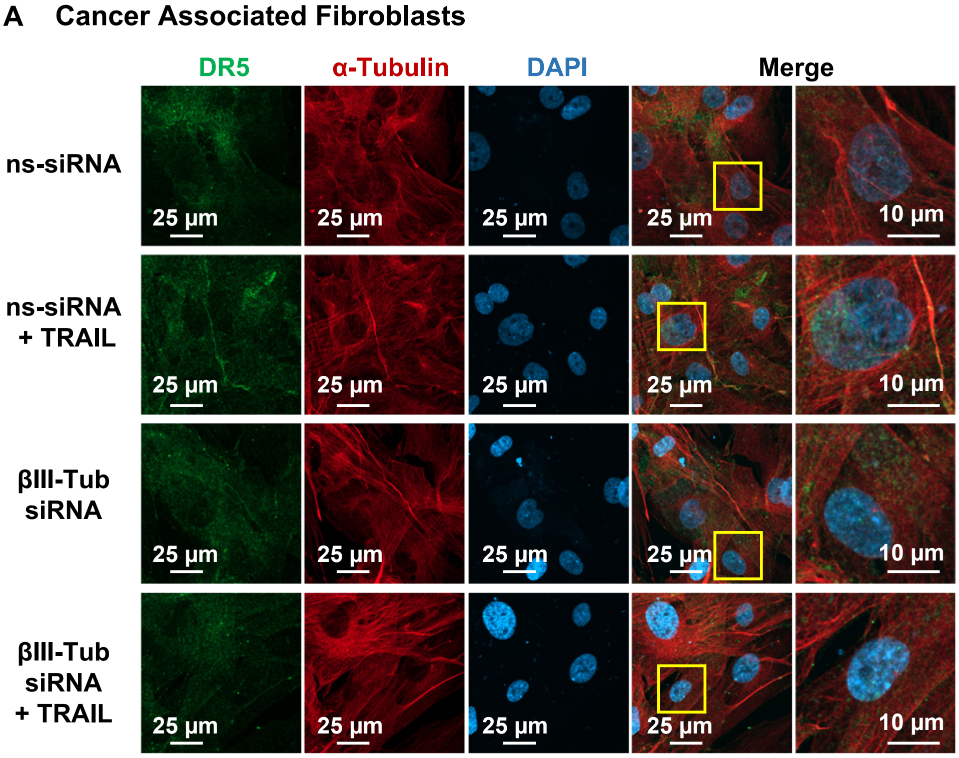

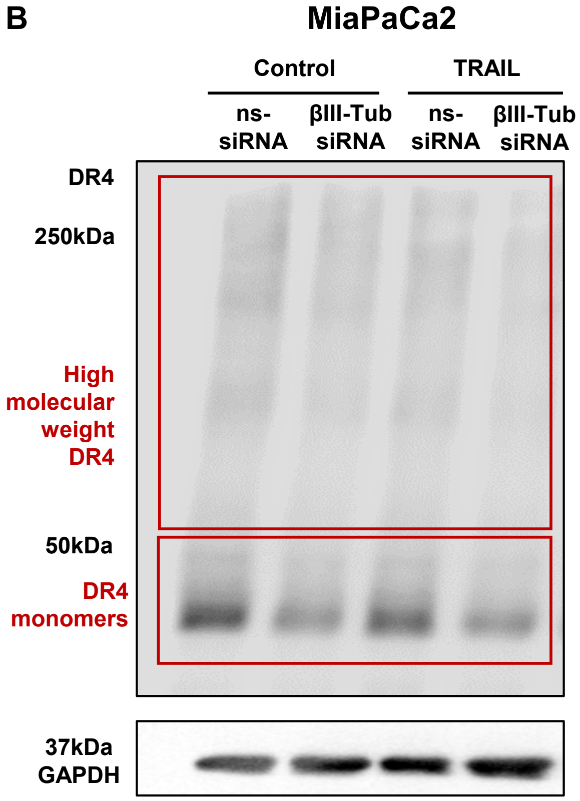

Supplementary Figure 6. βIII-Tubulin regulates DR5 dynamics in PDAC cells. (A) Immunofluorescence staining for DR5 and α-tubulin in cancer-associated fibroblasts (CAFs) with βIII-tubulin (βIII-Tub) knockdown (representative of n=5 independent experiments from 5 patient derived CAF lines). **(B)** Western blot for DR4 was performed under non-reducing conditions using protein extracted from MiaPaCa2 cells at 72 hours post transfection. βIII-tubulin silencing did not induce high-molecular weight multimeric clusters of DR4. Membranes were re-probed for GAPDH.

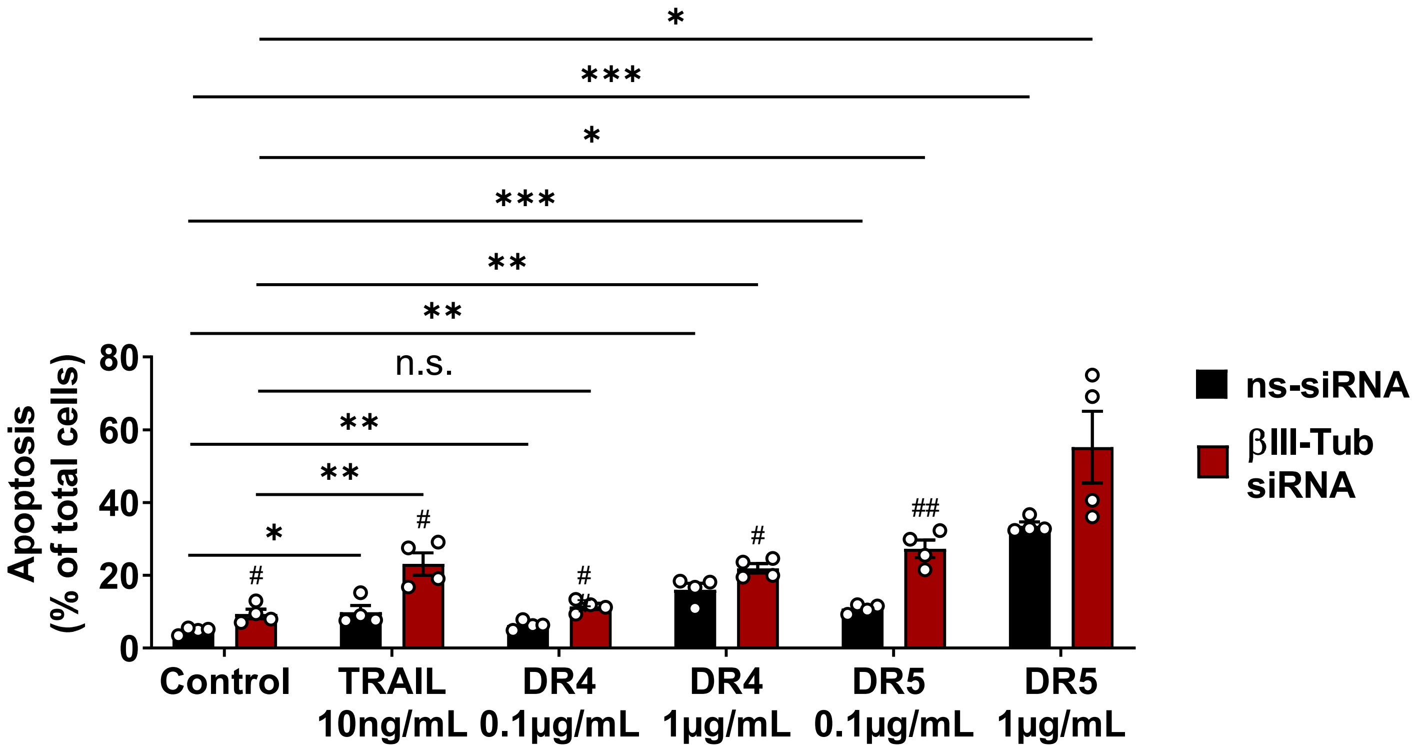

Supplementary Figure 7: βIII-tubulin knockdown in PDAC cells sensitises them to DR5 agonist to a greater extent than DR4 agonist. Apoptosis measured using flow cytometry for Annexin V/DAPI in MiaPaCa2 cells transfected with non-silencing (ns) or βIII-tubulin (βIII-Tub) siRNA and treated ± DR4 or DR5 agonists (n=4). Bars represent mean of n=4 independent experiments (individual data points shown from independent experiments) ± standard error of mean. Asterisks indicate significant as assessed by one-way ANOVA (*p≤0.05, **p≤0.01, ***p≤0.001, n.s.; non-significant). # symbol indicates significance relative to ns-siRNA at same TRAIL, DR4 or DR5 agonist concentration as assessed by one-way ANOVA (##p≤0.01, ###p≤0.001).

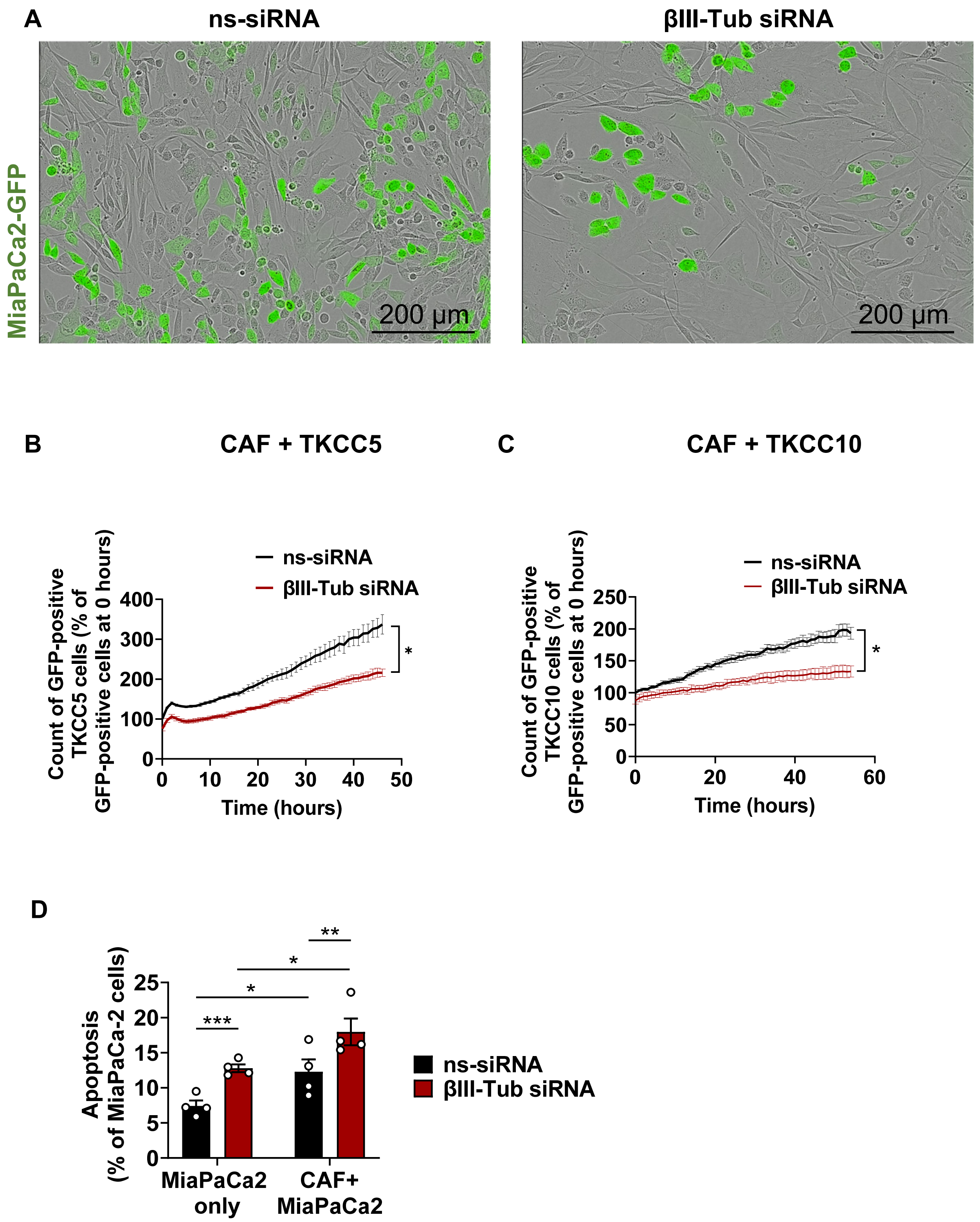

Supplementary Figure 8: Anti-proliferative effects of βIII-tubulin silencing in PDAC cells co-cultured with CAFs. Cell proliferation was reduced in PDAC cells with βIII-tubulin (βIII-Tub) knockdown when co-cultured with CAFs, as measured on an IncuCyte® S3 Live-Cell Analysis System. **(A)** Representative images show GFP-labelled PDAC cells surrounded by CAFs visible via phase contrast. **(B-C)** GFP-labelled TKCC5 (n=3 independent experiments) **(B)** and TKCC10 (n=3 independent experiments) **(C)** cells were transfected with non-silencing siRNA (ns-siRNA) or βIII-tubulin siRNA and co-cultured in the presence of primary patient-derived CAFs. The integrated IncuCyte software was used to count the number of GFP-positive PDAC cells. Line represents mean ± standard error of mean. n=3 indicates 3 independent experiments using CAFs isolated from 3 different PDAC patients. Asterisks indicate significance as assessed by two-tailed paired t-test (*p≤0.05). **(D)** Apoptosis (AnnexinV/DAPI) in MiaPaCa-2 cells with βIII-tubulin knockdown and indirectly co-cultured with CAFs (n=4 independent patients). Asterisks indicate significance as assessed by one-way ANOVA (*p≤0.05, ** p≤0.01, *** p≤0.001).

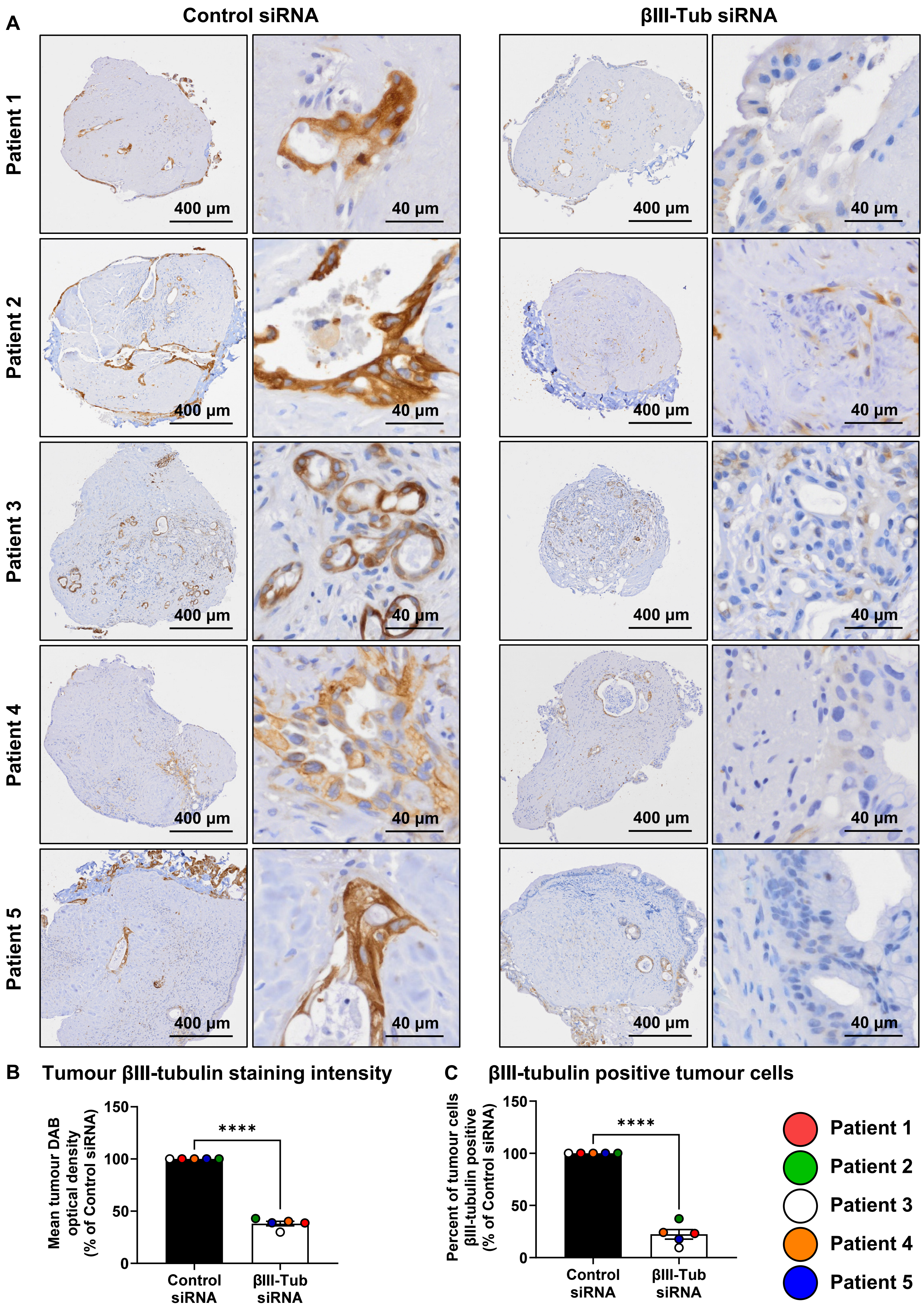
A
Control siRNA
βIII-Tub siRNA
Patient 1
Patient 2
Patient 3
Patient 4
Patient 5
400 μm
40 μm
B
Tumour βIII-tubulin staining intensity
Mean tumour DAB optical density (% of Control siRNA)
150
100
50
0
****
Control siRNA
βIII-Tub siRNA
C
βIII-tubulin positive tumour cells
Percent of tumour cells βIII-tubulin positive (% of Control siRNA)
150
100
50
0
****
Control siRNA
βIII-Tub siRNA
Patient 1
Patient 2
Patient 3
Patient 4
Patient 5

Supplementary Figure 9: Star 3 nanoparticle delivery of βIII-tubulin siRNA led to a potent reduction in βIII-tubulin expression in patient-derived PDAC explants. **(A)** Human PDAC explants from 5 patients were harvested at day 12 after treatment with Star 3+control siRNA or Star 3+βIII-tubulin siRNA on day 0, 3, 6 and 9. Representative images show immunohistochemistry for βIII-tubulin. **(B-C)** Quantification of βIII-tubulin immunohistochemistry using QuPath demonstrated a reduction in the overall intensity of βIII-tubulin expression **(B)** and a reduction in the number of cells positive for βIII-tubulin **(C)** in explants treated with βIII-tubulin siRNA. Data combined from n=5 patients taking the average quantification of 2-4 explants from each patient. Bars represent mean from n=5 patients (individual data points shown from each patient) ± standard error of mean. Asterisks indicate significance as assessed by One-Way ANOVA (n.s.; non-significant).

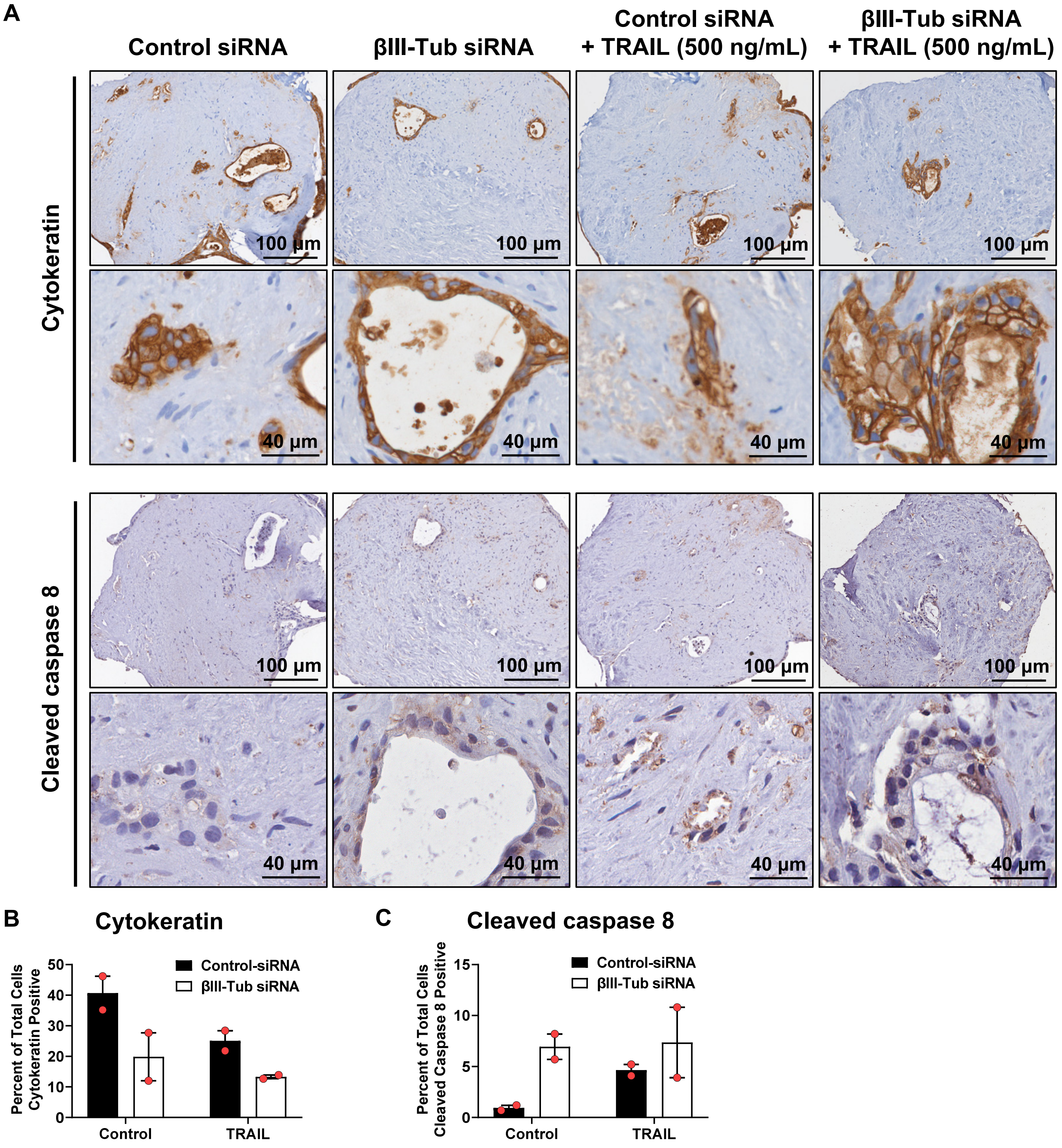

Supplementary Figure 10: βIII-tubulin silencing combined with TRAIL in Patient 1 decreased tumour cell number and increased extrinsic apoptosis in PDAC patient-derived explants. Pancreatic ductal adenocarcinoma (PDAC) tumour explants derived from Patient 1 were treated with Star 3 + βIII-tubulin (βIII-Tub) siRNA on days 0, 3, 6, and 9, and TRAIL (500 ng/mL) on days 5 and 10, with 2 explants per treatment. **(A)** Representative immunohistochemistry staining of serial sections for cytokeratin (tumour cell marker) and cleaved caspase 8 (extrinsic apoptosis marker) at low and high magnification. **(B-C)** Quantification of whole tumour explants was performed on QuPath for immunohistochemistry staining of cytokeratin **(B)** and cleaved caspase 8 **(C)**. Results show a reduction in tumour cell number with βIII-tubulin silencing combined with TRAIL compared to control explants. Caspase 8 cleavage was also increased in all treatment groups compared to control-siRNA untreated explants. Symbols represent quantification of individual whole tumour explant sections. Bars represent mean ± standard error of mean.

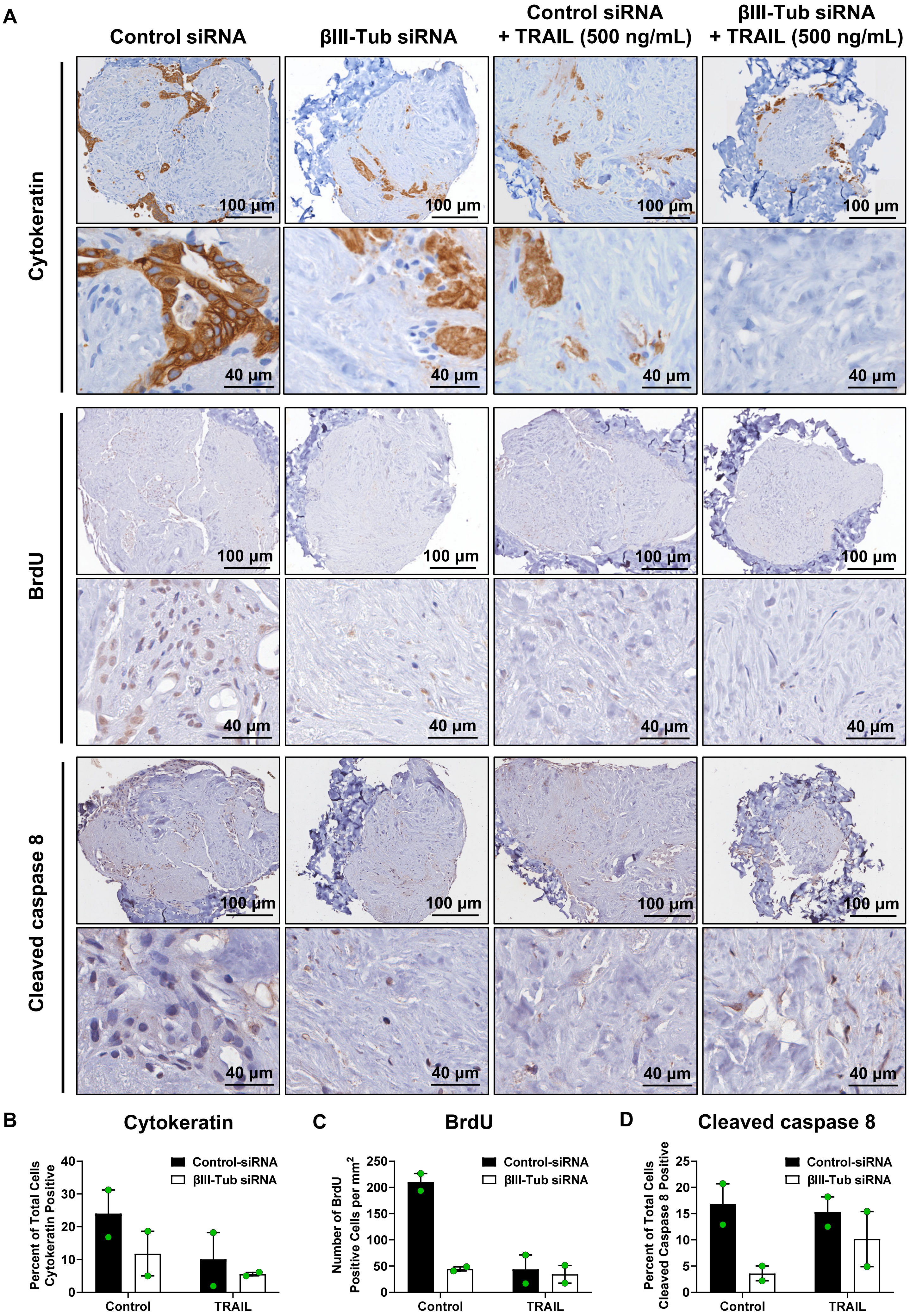
A
Control siRNA
βIII-Tub siRNA
Control siRNA + TRAIL (500 ng/mL)
βIII-Tub siRNA + TRAIL (500 ng/mL)
Cytokeratin
BrdU
Cleaved caspase 8
100 μm
40 μm
B
Cytokeratin
Percent of Total Cells Cytokeratin Positive
Control-siRNA
βIII-Tub siRNA
Control
TRAIL
C
BrdU
Number of BrdU Positive Cells per mm²
D
Cleaved caspase 8
Percent of Total Cells Cleaved Caspase 8 Positive

Supplementary Figure 11: βIII-tubulin silencing combined with TRAIL in Patient 2 decreased tumour cell number and decreased cell proliferation in PDAC patient-derived explants. Pancreatic ductal adenocarcinoma (PDAC) tumour explants derived from Patient 2 were treated with Star 3 + βIII-tubulin (βIII-Tub) siRNA on days 0, 3, 6, and 9, and TRAIL (500 ng/mL) on days 5 and 10, with 2 explants per treatment. **(A)** Representative immunohistochemistry staining of serial sections for cytokeratin (tumour cell marker), bromodeoxyuridine (BrdU) (proliferation marker), and cleaved caspase 8 (extrinsic apoptosis marker) at low and high magnification. **(B-D)** Quantification of whole tumour explants was performed on QuPath for immunohistochemistry staining of cytokeratin **(B)**, BrdU **(C)**, and cleaved caspase 8 **(D)**. Results show a reduction in tumour cell number in tumour explants with βIII-tubulin silencing combined with TRAIL compared to control explants. Cell proliferation was also reduced in all treatment groups compared to control-siRNA untreated explants. Symbols represent quantification of individual whole tumour explant sections. Bars represent mean ± standard error of mean.

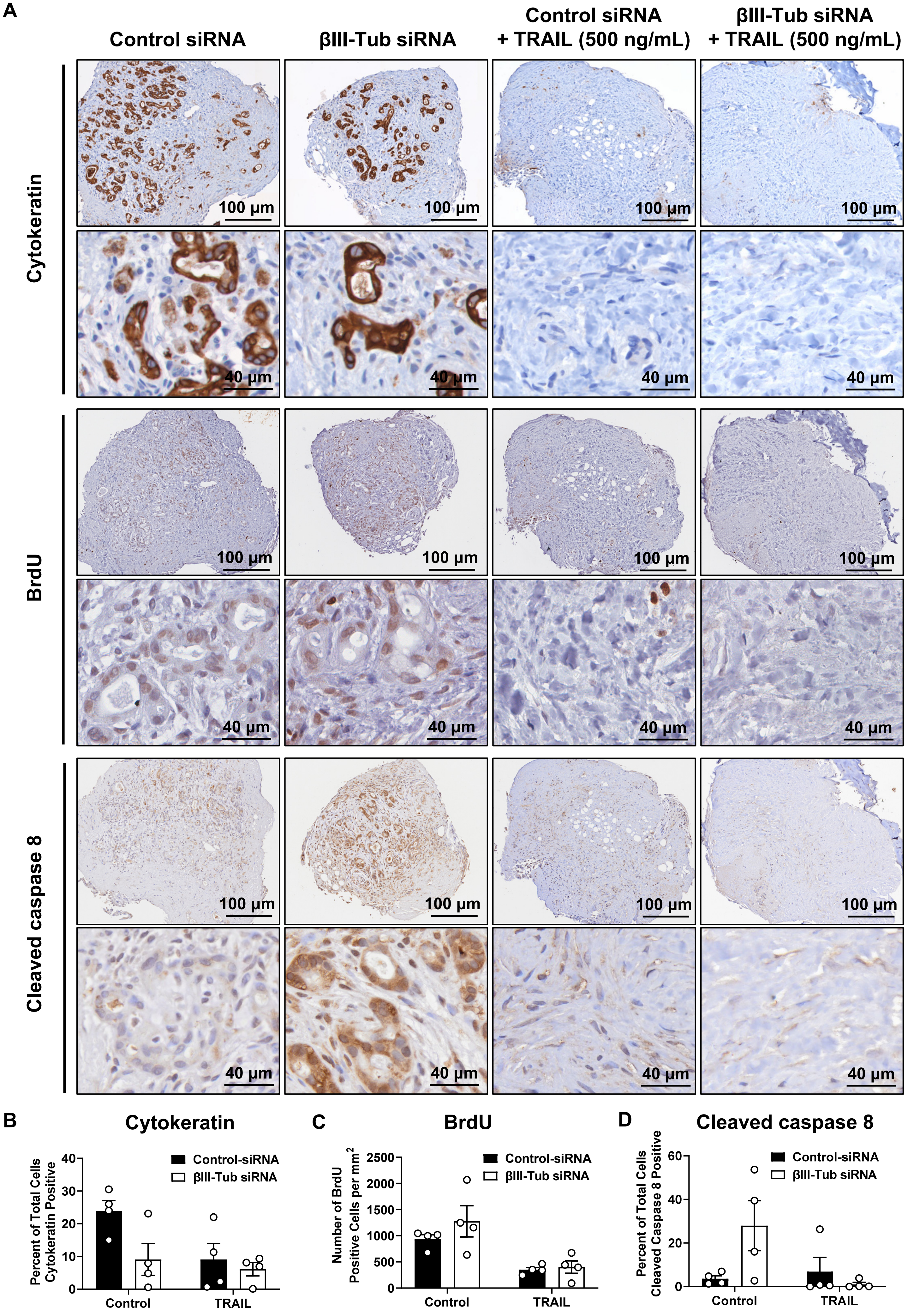
A
Control siRNA
βIII-Tub siRNA
Control siRNA + TRAIL (500 ng/mL)
βIII-Tub siRNA + TRAIL (500 ng/mL)
Cytokeratin
BrdU
Cleaved caspase 8
100 μm
40 μm
B
Cytokeratin
Percent of Total Cells Cytokeratin Positive
Control-siRNA
βIII-Tub siRNA
Control
TRAIL
C
BrdU
Number of BrdU Positive Cells per mm^2
D
Cleaved caspase 8
Percent of Total Cells Cleaved Caspase 8 Positive

Supplementary Figure 12: βIII-tubulin silencing combined with TRAIL in Patient 3 decreased tumour cell number and decreased cell proliferation in PDAC patient-derived explants. Pancreatic ductal adenocarcinoma (PDAC) tumour explants derived from Patient 3 were treated with Star 3 + βIII-tubulin (βIII-Tub) siRNA on days 0, 3, 6, and 9, and TRAIL (500 ng/mL) on days 5 and 10, with 4 explants per treatment. **(A)** Representative immunohistochemistry staining of serial sections for cytokeratin (tumour cell marker), bromodeoxyuridine (BrdU) (proliferation marker), and cleaved caspase 8 (extrinsic apoptosis marker) at low and high magnification. Representative images are also shown in **Figure 7** and are reproduced here showing quantification of each stain from Patient 3. **(B-D)** Quantification of whole tumour explants was performed on QuPath for immunohistochemistry staining of cytokeratin **(B)**, BrdU **(C)**, and cleaved caspase 8 **(D)**. Results show a reduction in tumour cell number in tumour explants with βIII-tubulin silencing alone and TRAIL treatment alone compared to control-siRNA untreated explants with no further decrease in combination treated explants. BrdU staining was reduced in all TRAIL treated explants, and caspase 8 cleavage was increased in tumour explants with βIII-tubulin silencing alone compared to control-siRNA untreated explants. Symbols represent quantification of individual whole tumour explant sections. Bars represent mean ± standard error of mean.

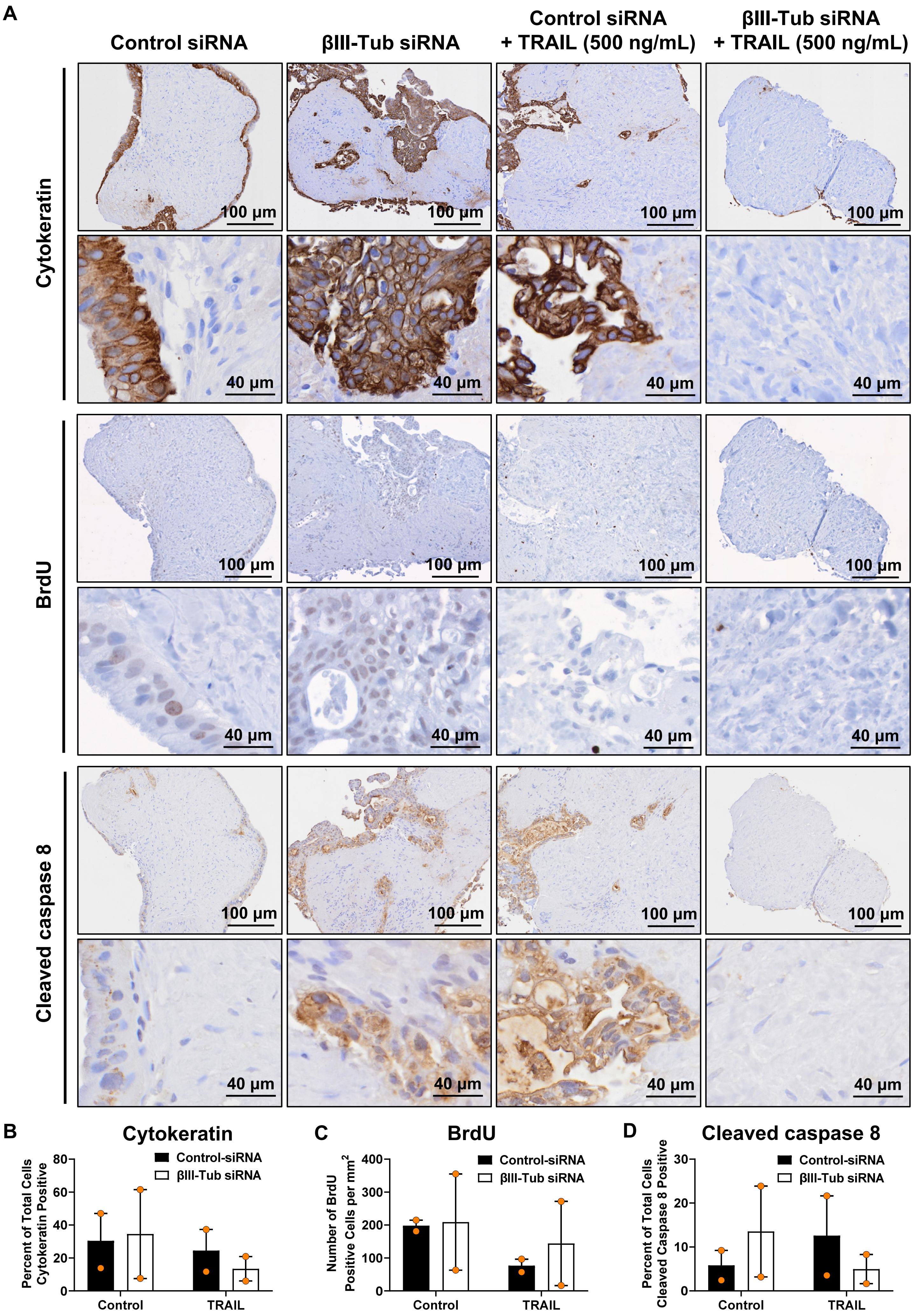
A
Control siRNA
βIII-Tub siRNA
Control siRNA + TRAIL (500 ng/mL)
βIII-Tub siRNA + TRAIL (500 ng/mL)
Cytokeratin
BrdU
Cleaved caspase 8
100 μm
40 μm
B
Cytokeratin
Percent of Total Cells Cytokeratin Positive
Control-siRNA
βIII-Tub siRNA
Control
TRAIL
C
BrdU
Number of BrdU Positive Cells per mm²
D
Cleaved caspase 8
Percent of Total Cells Cleaved Caspase 8 Positive

Supplementary Figure 13: βIII-tubulin silencing combined with TRAIL in Patient 4 decreased tumour cell number in PDAC patient-derived explants. Pancreatic ductal adenocarcinoma (PDAC) tumour explants derived from Patient 4 were treated with Star 3 + βIII-tubulin (βIII-Tub) siRNA on days 0, 3, 6, and 9, and TRAIL (500 ng/mL) on days 5 and 10, with 2 explants per treatment. **(A)** Representative immunohistochemistry staining of serial sections for cytokeratin (tumour cell marker), bromodeoxyuridine (BrdU) (proliferation marker), and cleaved caspase 8 (extrinsic apoptosis marker) at low and high magnification. **(B-D)** Quantification of whole tumour explants was performed on QuPath for immunohistochemistry staining of cytokeratin **(B)**, BrdU **(C)**, and cleaved caspase 8 **(D)**. Results show a reduction in tumour cell number in tumour explants with βIII-tubulin silencing combined with TRAIL compared to control-siRNA untreated explants. Symbols represent quantification of individual whole tumour explant sections. Bars represent mean ± standard error of mean.

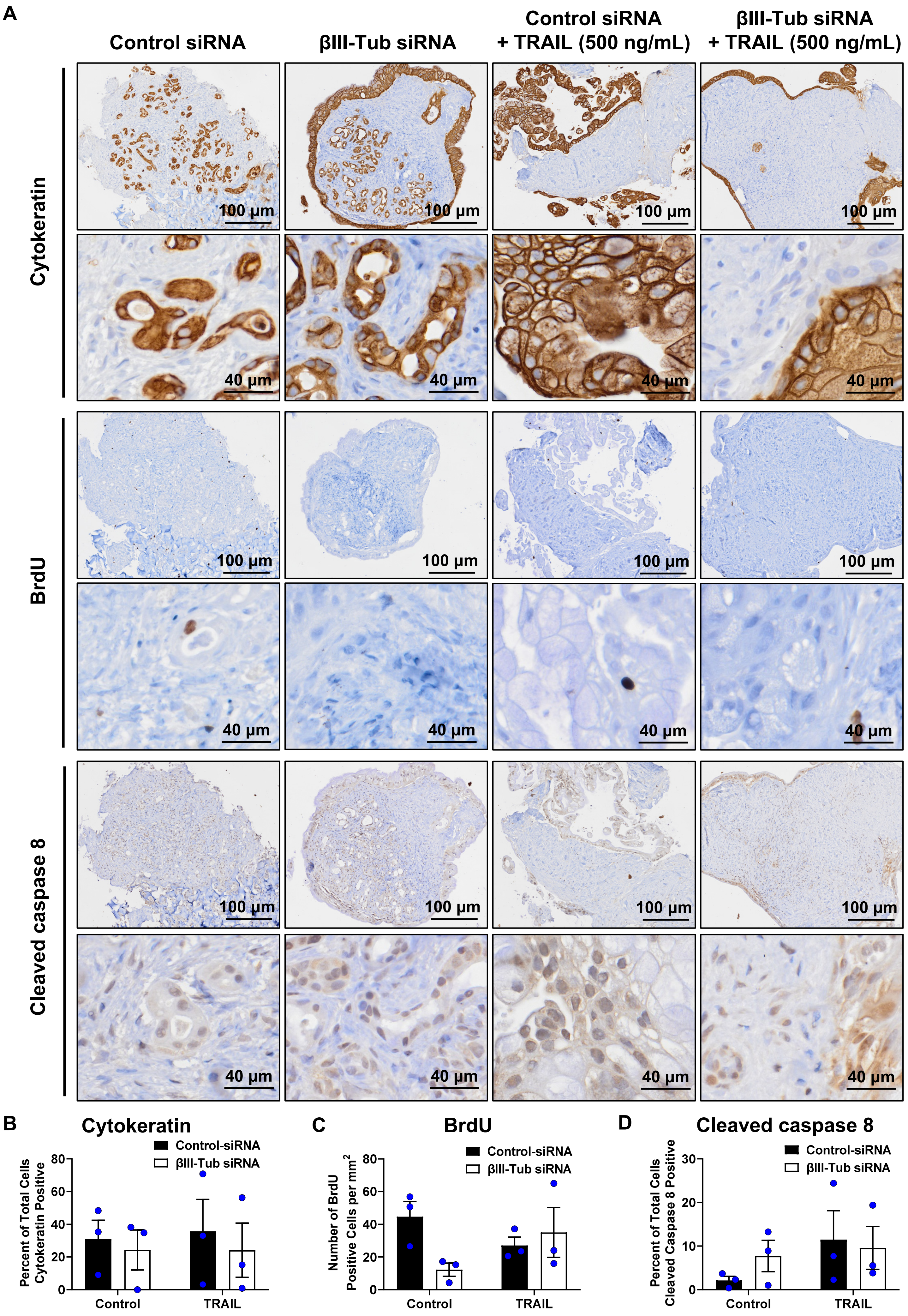
A
Control siRNA
βIII-Tub siRNA
Control siRNA + TRAIL (500 ng/mL)
βIII-Tub siRNA + TRAIL (500 ng/mL)
Cytokeratin
BrdU
Cleaved caspase 8
100 μm
40 μm
B
Cytokeratin
Percent of Total Cells Cytokeratin Positive
Control-siRNA
βIII-Tub siRNA
Control
TRAIL
C
BrdU
Number of BrdU Positive Cells per mm²
D
Cleaved caspase 8
Percent of Total Cells Cleaved Caspase 8 Positive

Supplementary Figure 14: βIII-tubulin silencing combined with TRAIL in Patient 5 increased extrinsic apoptosis in PDAC patient-derived explants. Pancreatic ductal adenocarcinoma (PDAC) tumour explants derived from Patient 5 were treated with Star 3 + βIII-tubulin (βIII-Tub) siRNA on days 0, 3, 6, and 9, and TRAIL (500 ng/mL) on days 5 and 10, with 3 explants per treatment. **(A)** Representative immunohistochemistry staining of serial sections for cytokeratin (tumour cell marker), bromodeoxyuridine (BrdU) (proliferation marker), and cleaved caspase 8 (extrinsic apoptosis marker) at low and high magnification. **(B-D)** Quantification of whole tumour explants was performed on QuPath for immunohistochemistry staining of cytokeratin **(B)**, BrdU **(C)**, and cleaved caspase 8 **(D)**. Results show an increase in caspase 8 cleavage in tumour explants with βIII-tubulin silencing alone and TRAIL treatment alone compared to control-siRNA untreated explants. There was no further increase in caspase 8 cleavage with combination treatment compared to individual treatments. Symbols represent quantification of individual whole tumour explant sections. Bars represent mean ± standard error of mean.

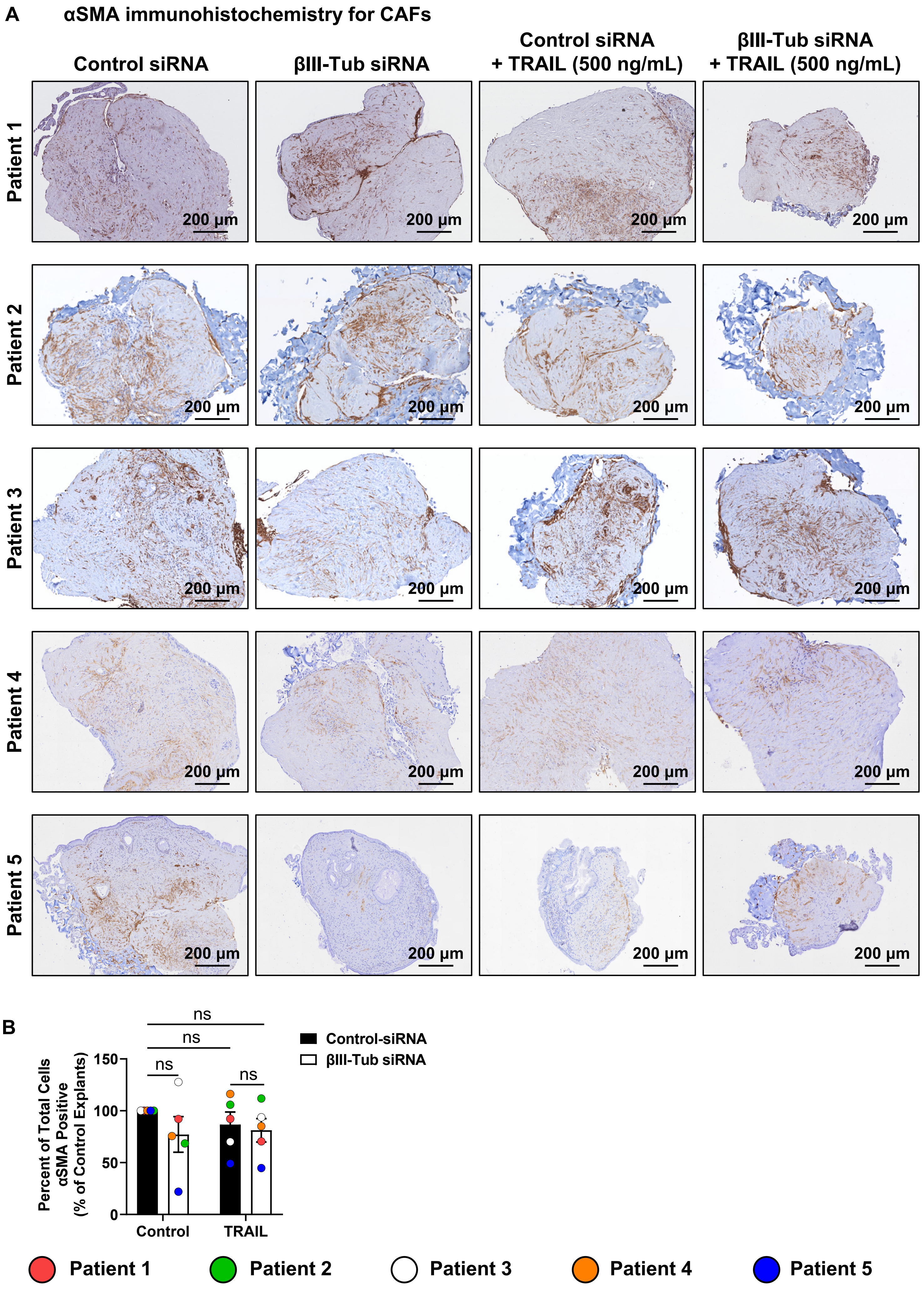
A
αSMA immunohistochemistry for CAFs
Control siRNA
βIII-Tub siRNA
Control siRNA + TRAIL (500 ng/mL)
βIII-Tub siRNA + TRAIL (500 ng/mL)
Patient 1
Patient 2
Patient 3
Patient 4
Patient 5
200 μm
B
Percent of Total Cells αSMA Positive (% of Control Explants)
150
100
50
0
Control
TRAIL
ns
Control-siRNA
βIII-Tub siRNA
Patient 1
Patient 2
Patient 3
Patient 4
Patient 5

Supplementary Figure 15: βIII-tubulin silencing and TRAIL treatment had patient-specific effects on CAF cell number in PDAC tumour explants. (A) Representative immunohistochemistry staining of cancer-associated fibroblast (CAF) marker α-smooth muscle actin (αSMA) in pancreatic ductal adenocarcinoma (PDAC) tumour explants from patients 1-5. **(B)** Quantification of whole tumour explants was performed on QuPath for immunohistochemistry staining of αSMA and data combined from n=5 patients taking the average quantification of 2-4 explants from each patient. Bars represent mean from n=5 patients (individual data points shown from each patient) ± standard error of mean. Asterisks indicate significance as assessed by One-Way ANOVA (n.s.; non-significant).

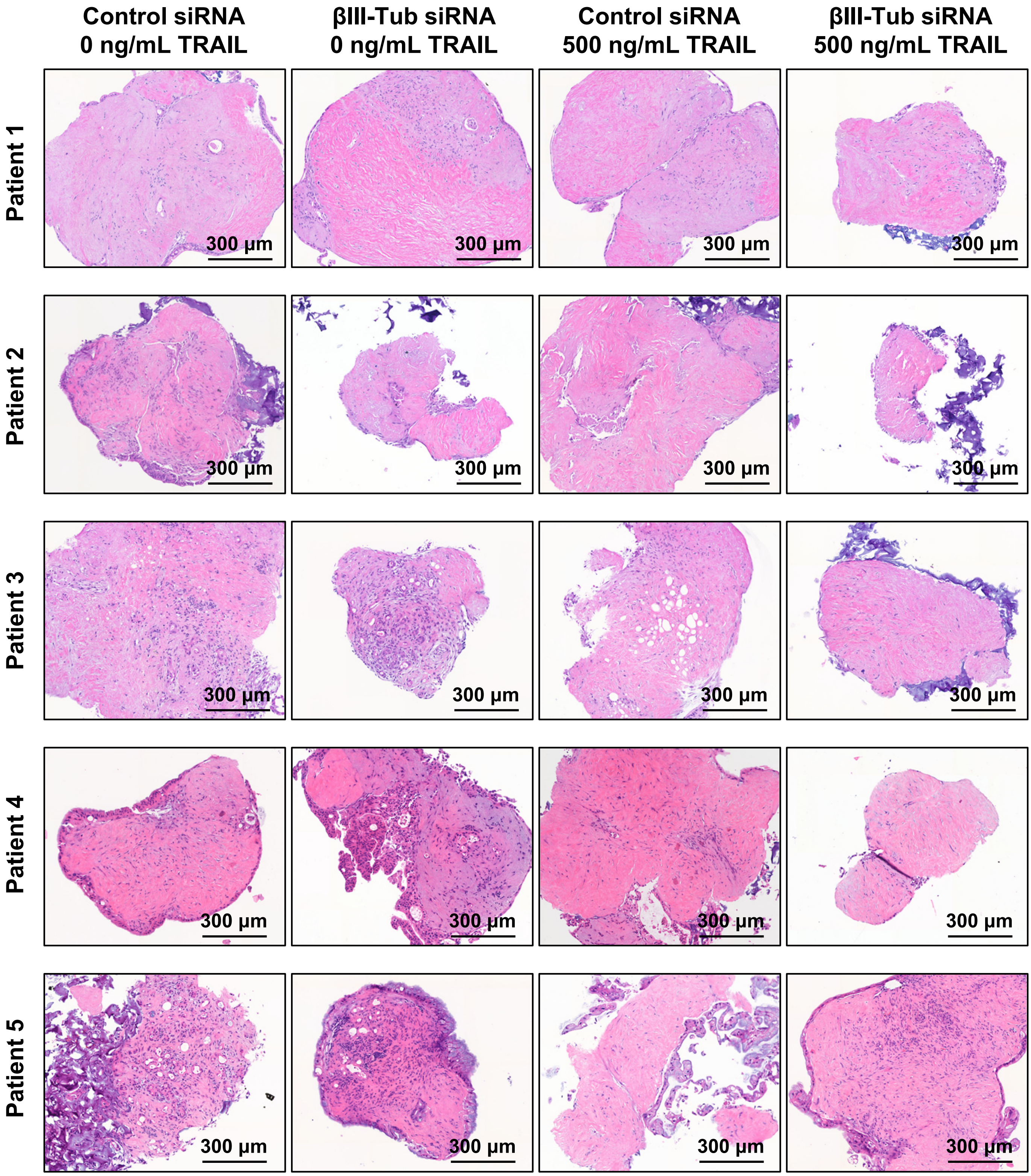

Supplementary Figure 16. H&E staining of human PDAC tumour explants from patients 1-6. Representative images show 1 explant from each treatment group per patient.

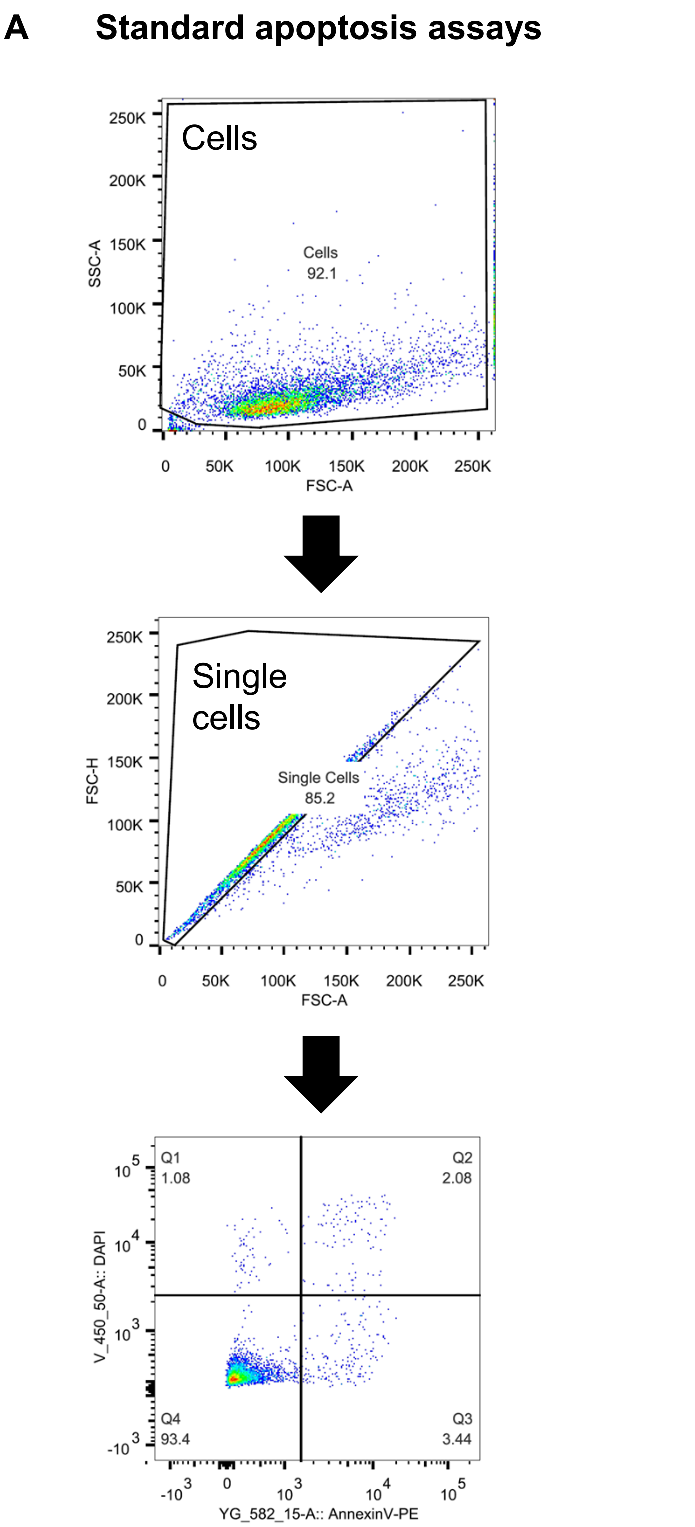

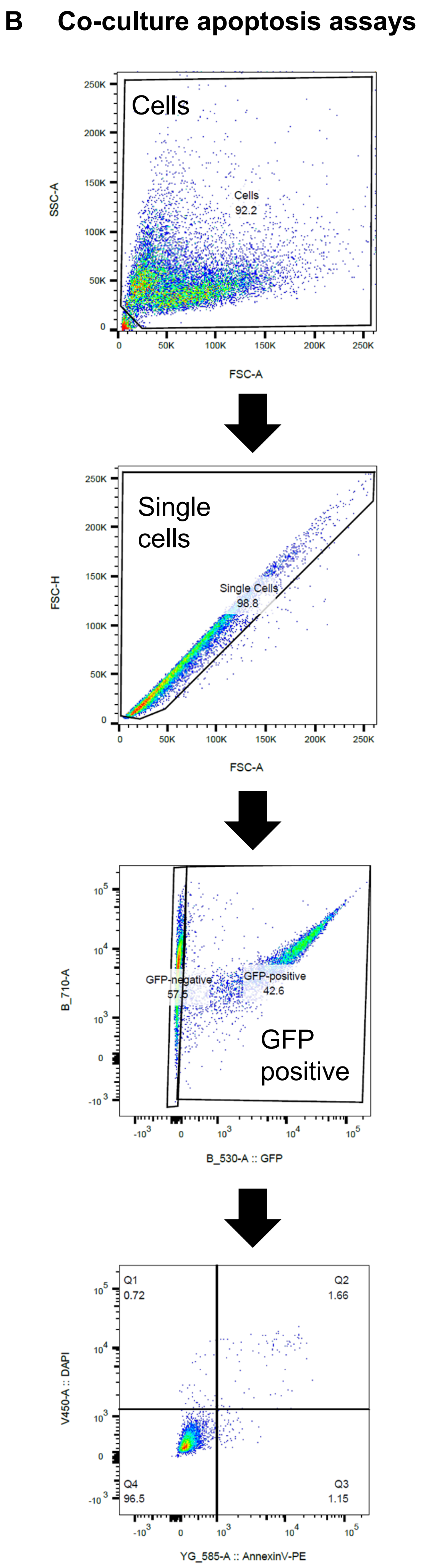

Supplementary Figure 17: Gating strategy for apoptosis assays. Apoptosis was measured via flow cytometry for Annexin V/DAPI using the FortessaSORP flow cytometer. **(A-B)** Panels show representative flow cytometry plots with gating strategies used for standard apoptosis assays **(A)** or co-culture apoptosis assays where apoptosis was measured in GFP positive PDAC cells **(B)**. Total apoptosis was assigned the sum of Q1+Q2+Q3 quadrants of Annexin V vs DAPI plots.

A **Phase contrast**

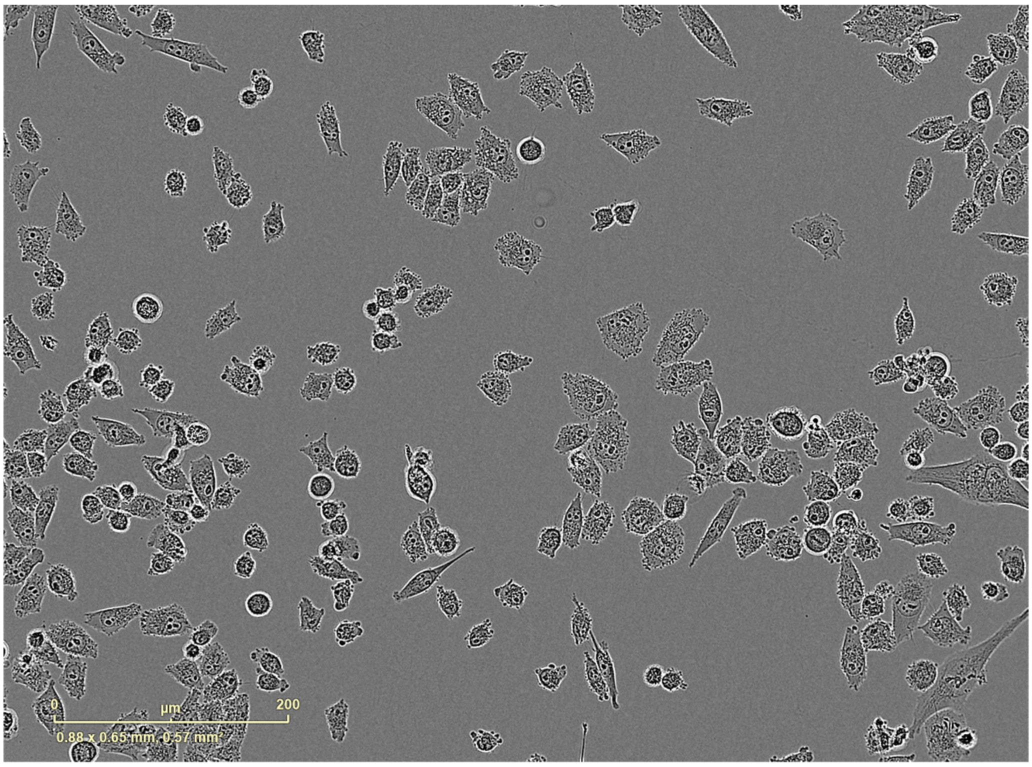

B **Confluence mask**

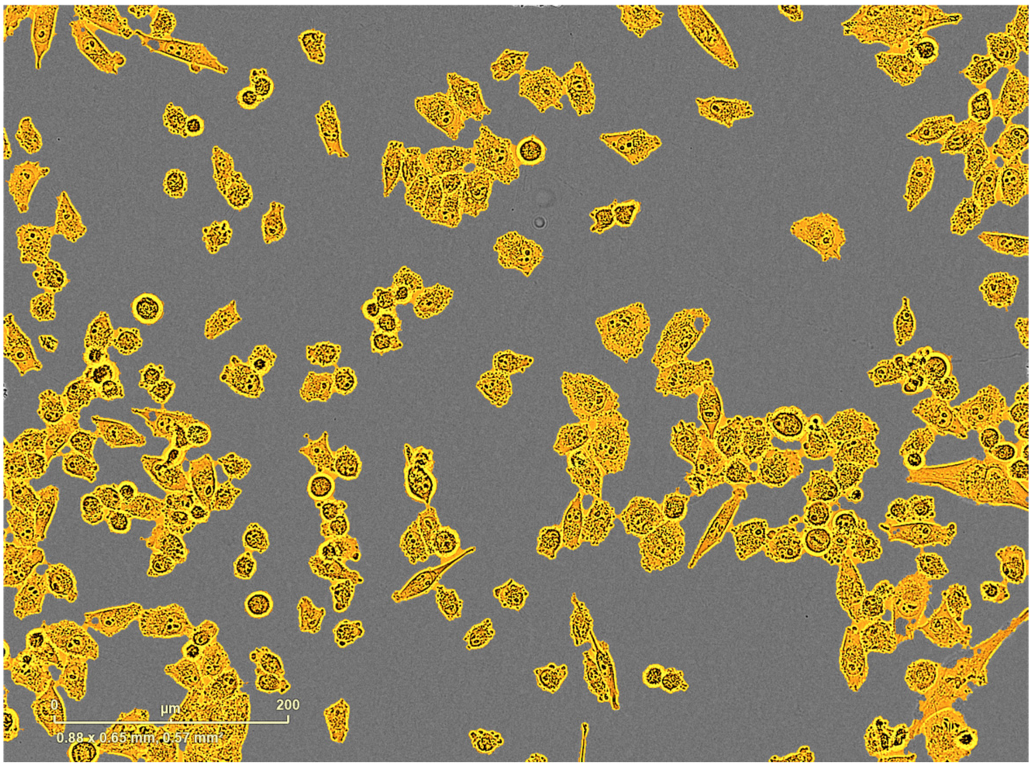

Supplementary Figure 18: Analysis of cell proliferation on IncuCyte® S3 using confluence metrics. Real-time cell proliferation was measured using IncuCyte® S3 Live Cell Analysis System. **(A)** Representative image shows phase contrast image of MiaPaCa2 cells. **(B)** Integrated confluence metrics on the IncuCyte® software was used to calculate the percent confluence of each sample at 1-hour intervals. Representative image shows confluence mask (yellow) applied by IncuCyte® software to calculate the percent confluence. At least 9 fields of view were captured from each well of a 12-well plate, and 16 fields of view from each well of a 6-well plate.

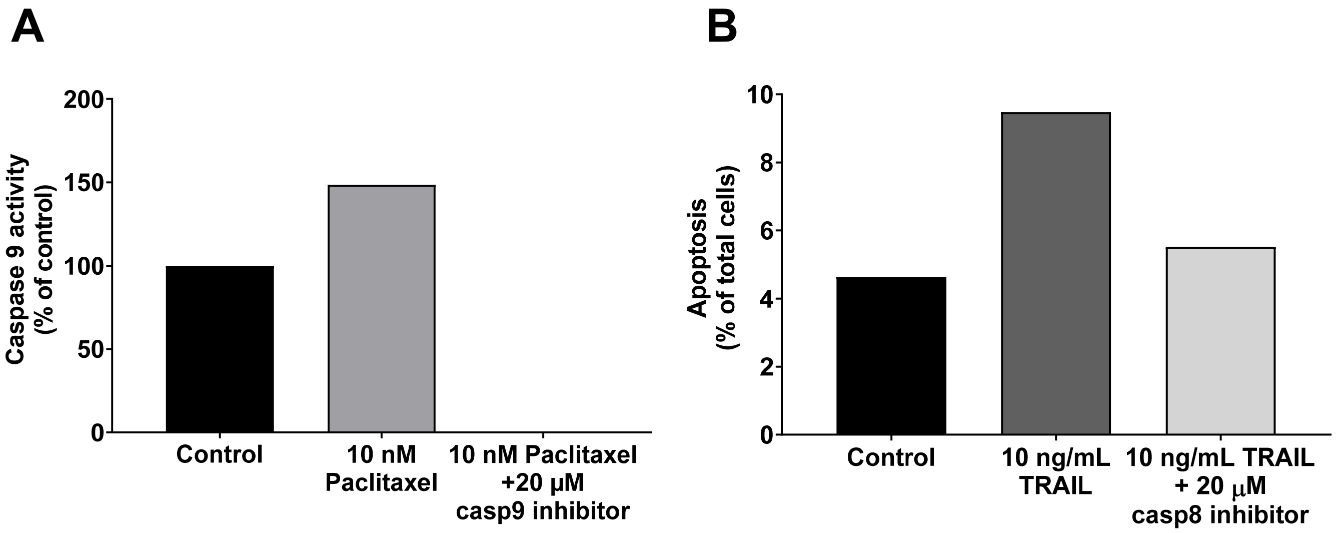

Supplementary Figure 19: Validation of caspase inhibitor activity. (A) Caspase 9 (casp9) inhibitor (Z-LEHD-FMK) potently blocks paclitaxel induced caspase 9 activity. MiaPaCa2 cells were pre-incubated with 20 µM caspase 9 inhibitor for 1 hour then treated with 10 nM paclitaxel for 24 hours (n=1 independent experiment). Caspase-9 activity was measured using CaspaseeGlo-9 luminescent assay and normalised to cell number using cell counting kit-8 (CCK-8) assay. **(B)** Caspase 8 (casp8) inhibitor (Q-IETD-OPh) blocks TRAIL induced apoptosis. MiaPaCa2 cells were pre-incubated with 20 µM caspase 8 inhibitor for 1 hour then treated with 10 ng/mL TRAIL for 24 hours (n=1). Apoptosis was measured using flow cytometry for Annexin V and DAPI.

Mouse IgG1

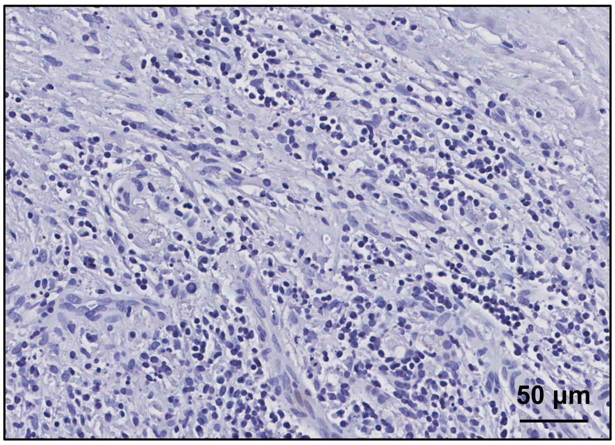

Mouse IgG2A

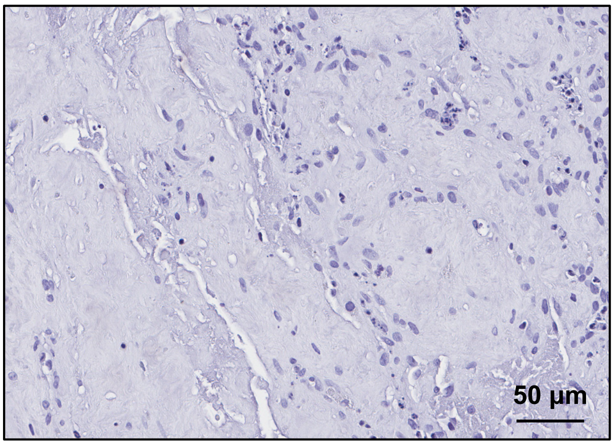

Rabbit IgG

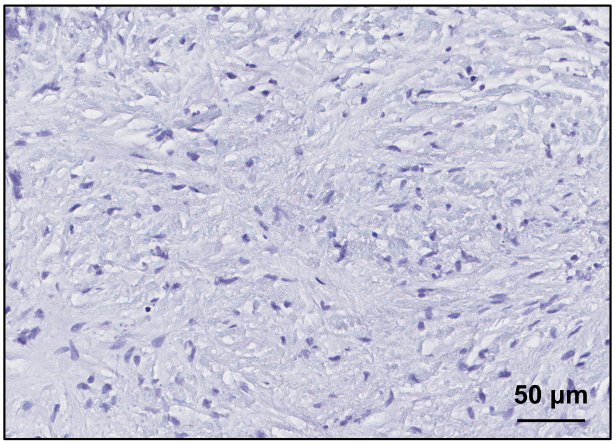

Supplementary Figure 20. Immunohistochemistry isotype controls. Representative images of each isotype control antibody used for immunohistochemistry. Mouse IgG1 was used for cytokeratin, cleaved caspase 8 (mouse), and BrdU. Mouse IgG2A was used for α-Smooth Muscle Actin and βIII-tubulin. Rabbit IgG was used for ki67 and cleaved caspase 8 (rabbit).

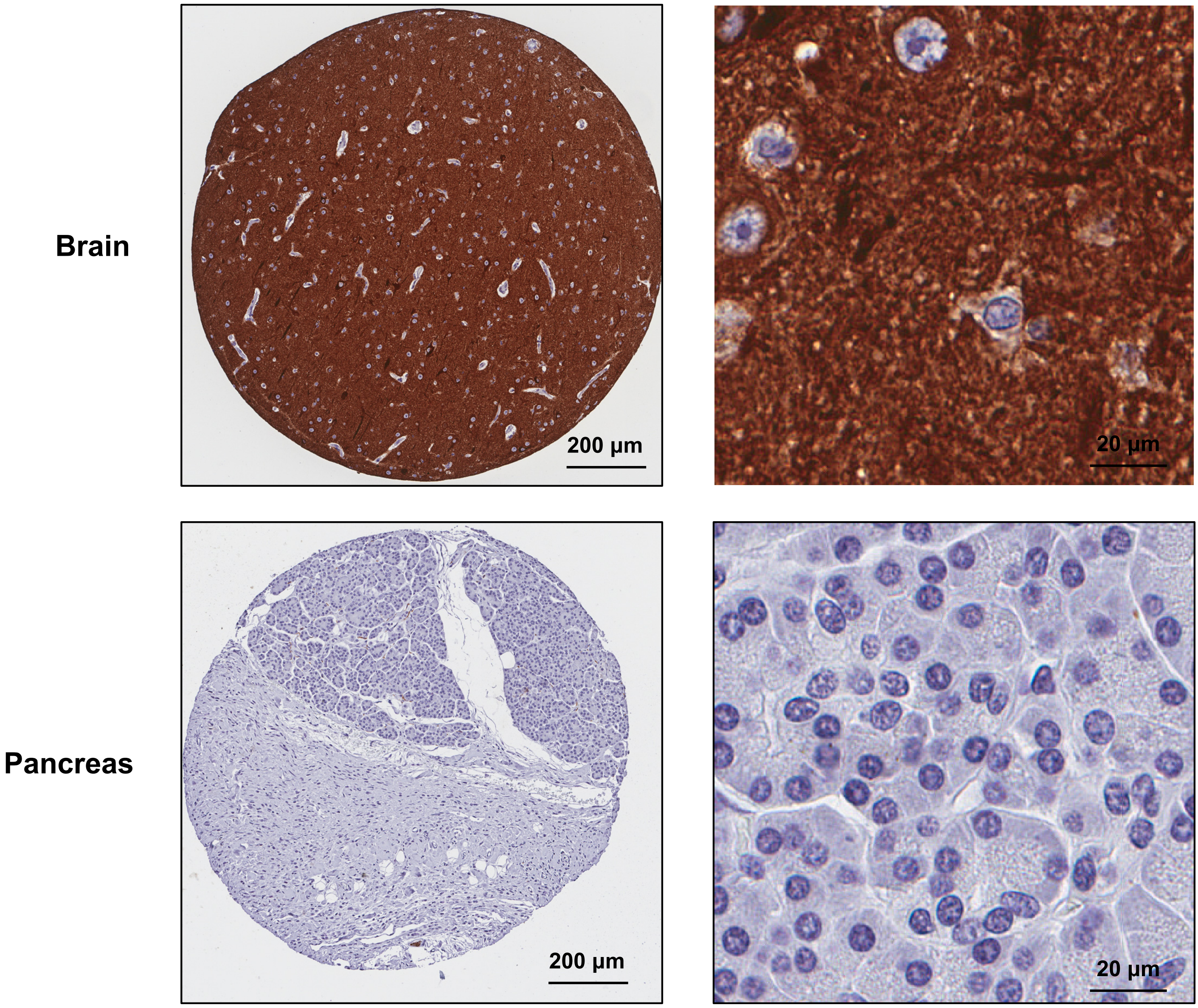

Supplementary Figure 21. Validation of βIII-tubulin antibody. Positive staining of βIII-tubulin (βIII-Tub) in human brain tissue (brown), and negative staining in normal pancreas tissue, consistent with expression in the human protein atlas.

Supplementary Movie 1. βIII-tubulin silencing triggered death receptor 5 (DR5) clustering in MiaPaCa2 cells prior to induction of cell death. Live imaging of GFP-tagged DR5 on the cell membrane of MiaPaCa2 cells using total internal reflection (TIRF) microscopy revealed that βIII-tubulin silencing induced DR5 membrane clustering. Cells were imaged 48 hours post transfection. Cells with βIII-tubulin knockdown that had visible DR5 membrane clustering showed characteristic features of apoptosis such as membrane blebbing. Cells were imaged for 50 minutes every 25 seconds, while being maintained in a humidified chamber at 37 °C and 5% CO2.

Supplementary Movie 2. βIII-tubulin silencing triggered death receptor 5 (DR5) clustering in MiaPaCa2 cells prior to induction of cell death. Live imaging of GFP-tagged DR5 in MiaPaCa2 cells using total internal reflection (TIRF) microscopy revealed that βIII-tubulin silencing induced highly dynamic clusters of DR5 at the cell membrane. Cells were imaged 48 hours post transfection with 50 ms exposure on PCO edge sCMOS (pixel size 0.097 μm), using TIRF settings, and 1000 frames were acquired per field of view.
