# Supplementary material for: βIII-tubulin can act as a brake on extrinsic apoptosis in pancreatic cancer
Source: Cell Death Dis. 2026 Apr 24;17(1):547. doi: 10.1038/s41419-026-08657-6 (PMC13243481; doi:10.1038/s41419-026-08657-6)
Supplement: Supplementary file 3 — Supplementary full western blots [file 41419_2026_8657_MOESM3_ESM.pdf]

βIII-Tubulin can act as a brake on extrinsic apoptosis in pancreatic cancer

Supplementary Data – Full Western Blots

- All lanes presented in this manuscript are demarcated with black boxes
- All unlabelled lanes indicate either replicate experiments or samples unrelated to the experiment presented in the manuscript.

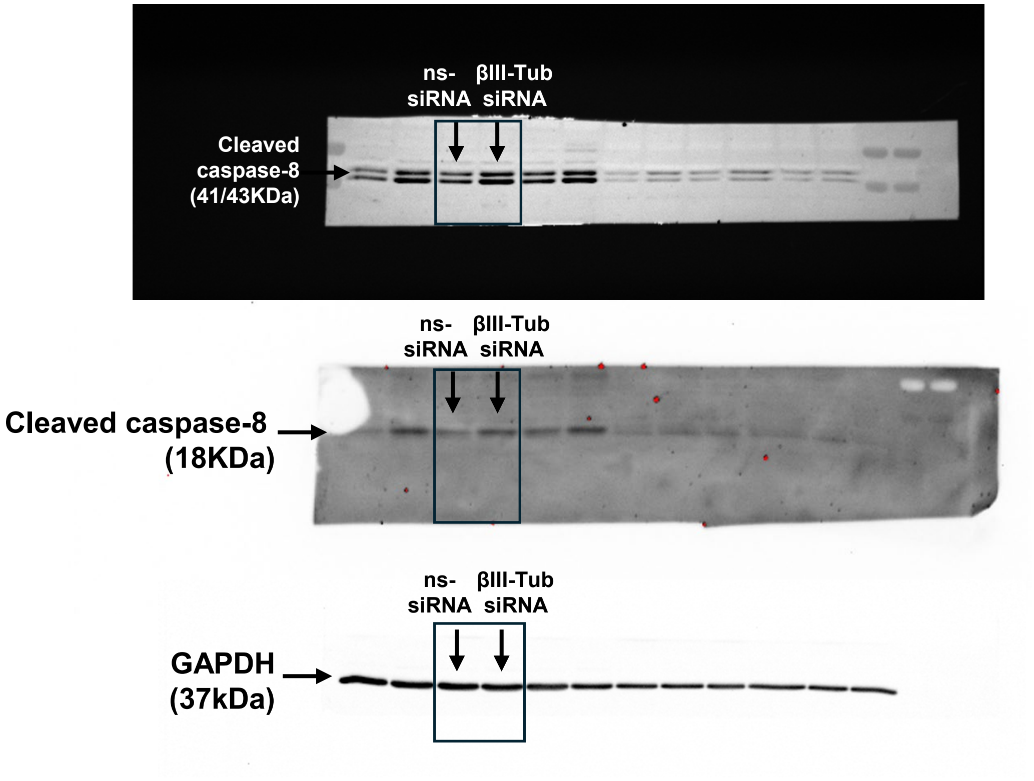

Figure 1G Raw Western Blots. Figure 1G membrane was cut into strips and imaged**.**

H

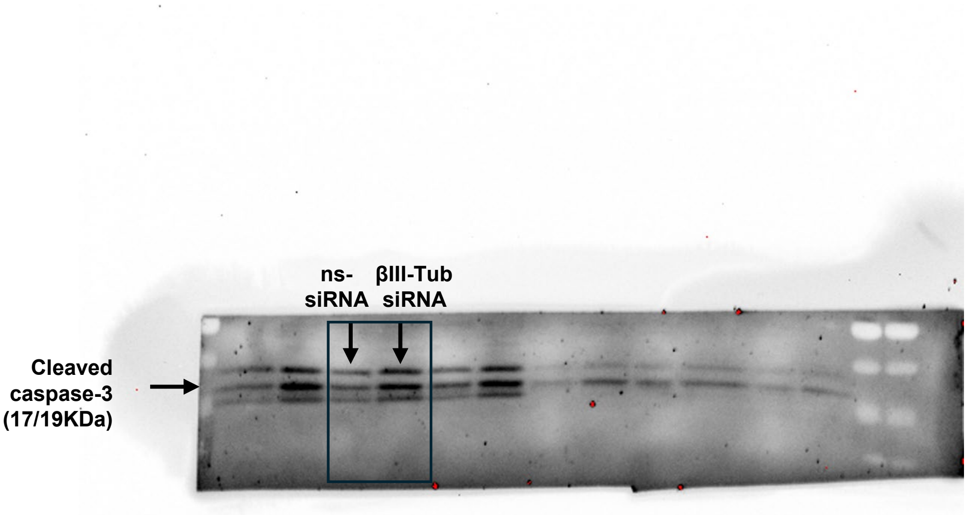

I

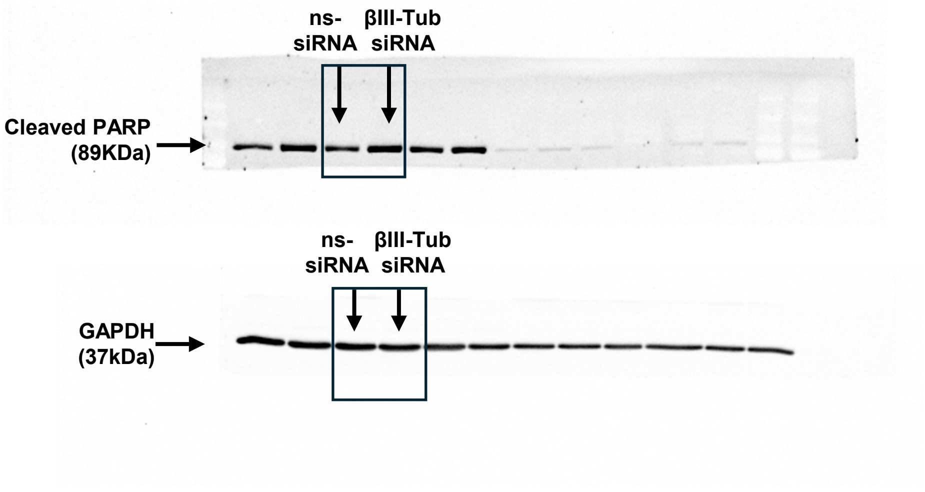

Figure 1H-I Raw Western Blots. Figure 1H & I membrane was cut into strips and imaged. 1H & 1I are the same membrane as 1G. 17-19kDa strip was re-probed for cleaved caspase-3.

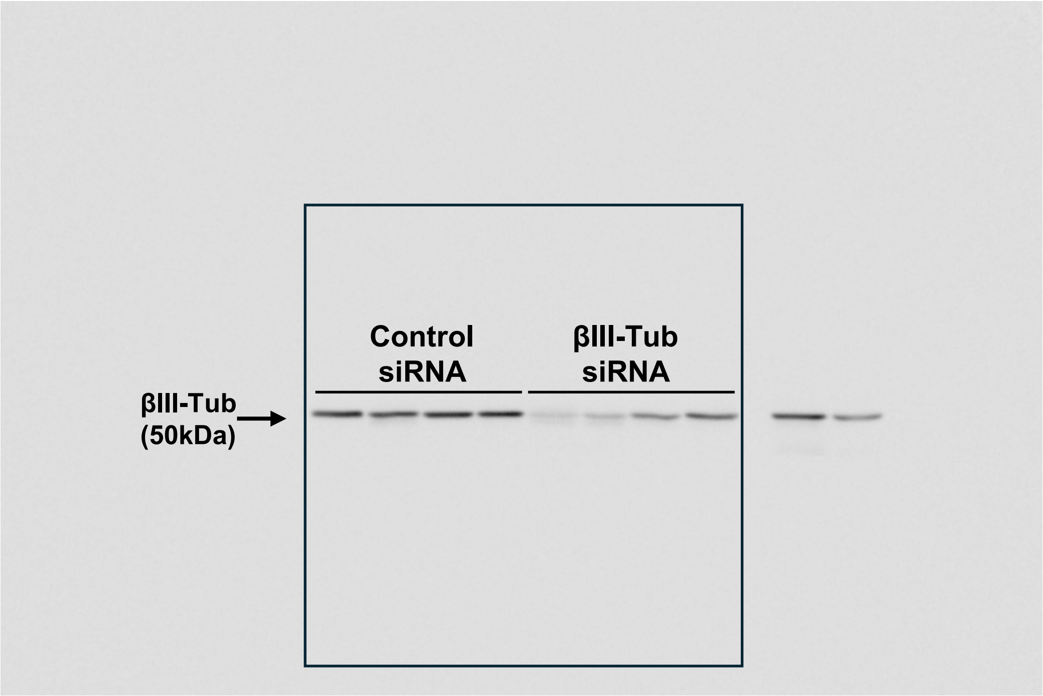

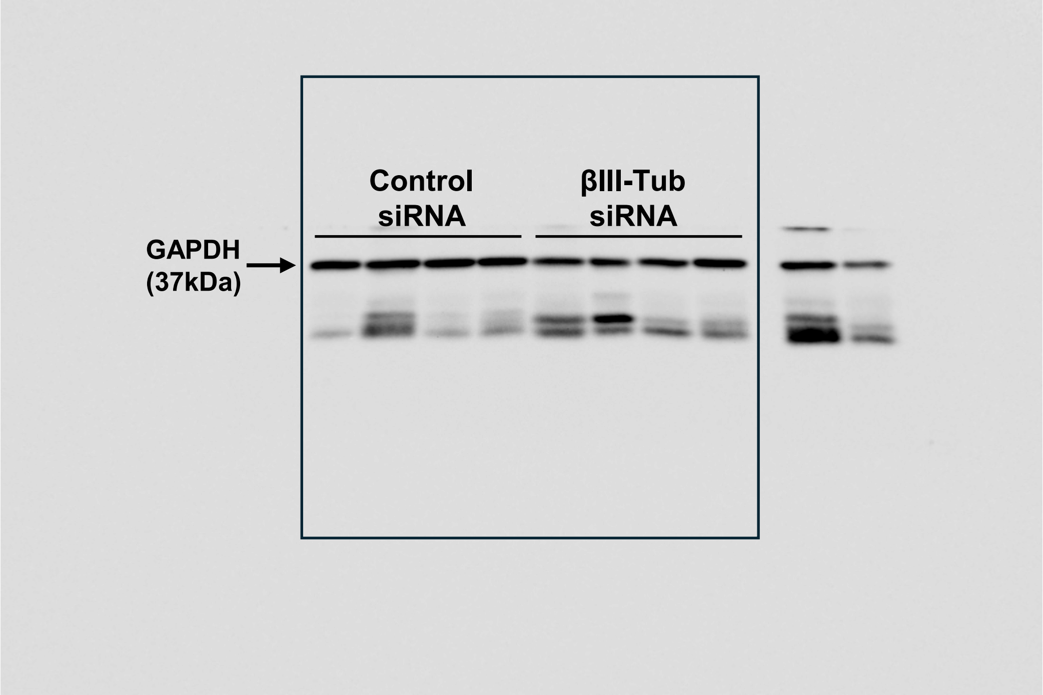

Figure 1K Raw Western Blots. Figure 1K membrane was cut into strips and imaged.

G

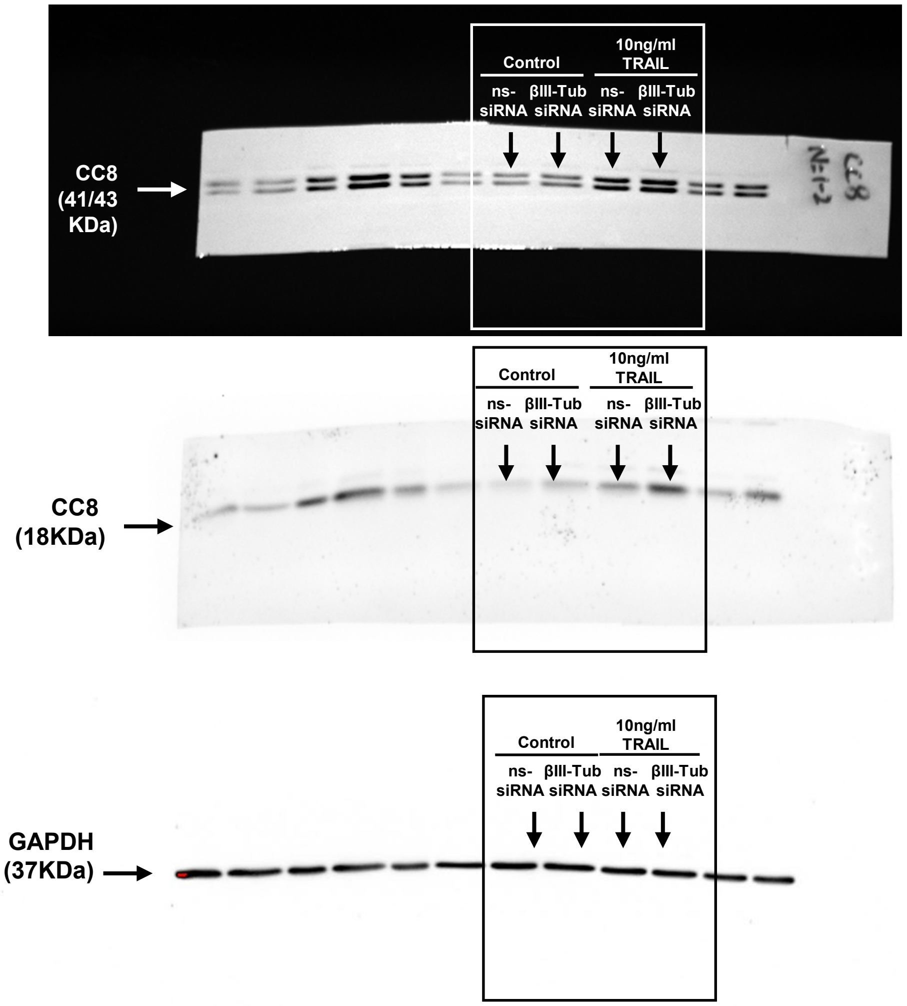

Figure 2G Raw Western Blots. Figure 2G membrane was cut into strips and imaged.

H

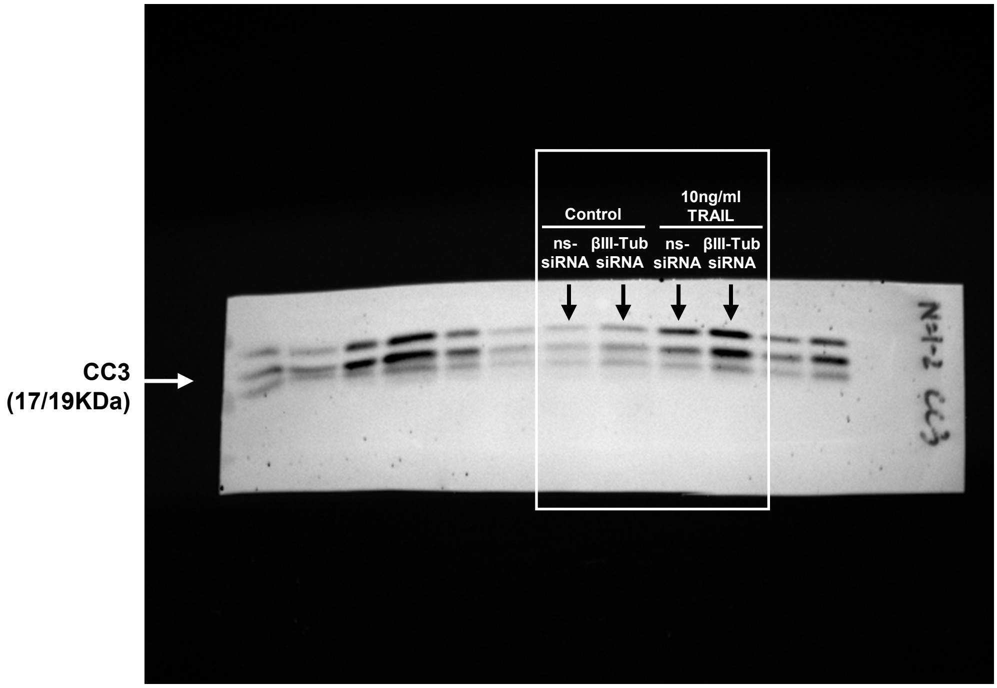

I

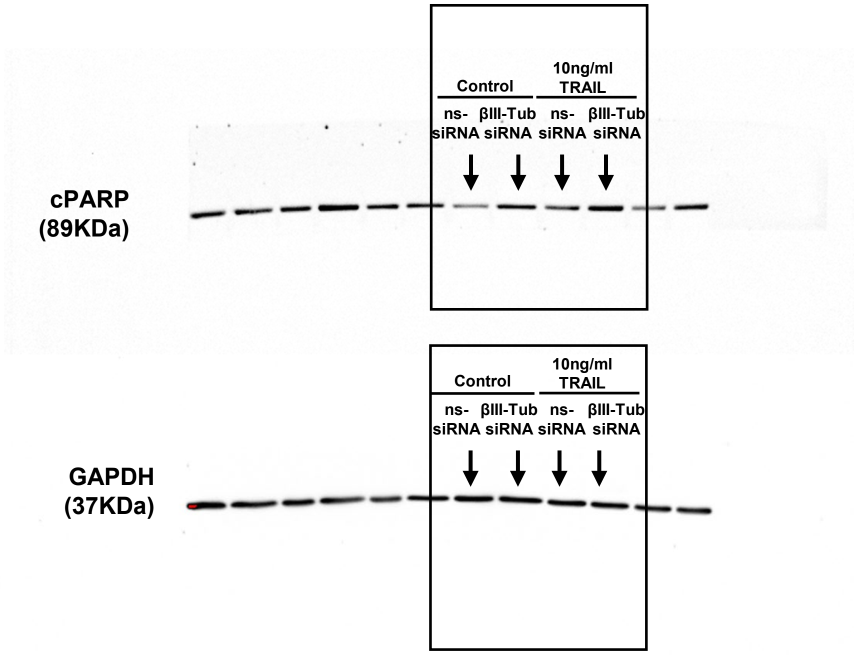

Figure 2H-I Raw Western Blots. Figure 2H & I membrane was cut into strips and imaged. 2H & 2I are the same membrane as 2G. 17-19kDa strip was re-probed for cleaved caspase-3.

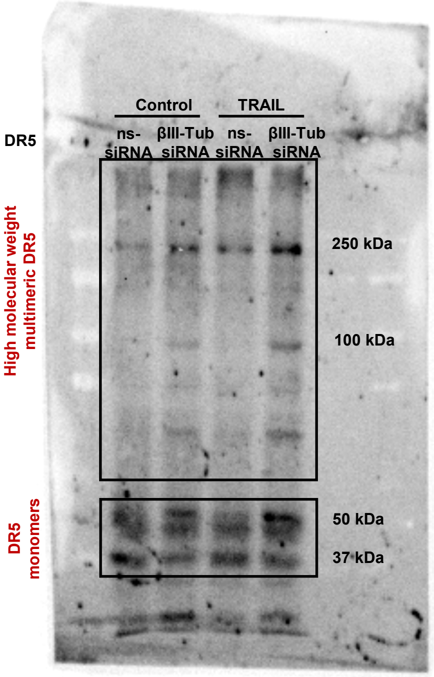

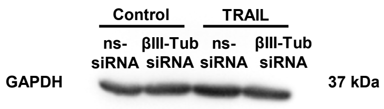

Figure 4F Raw Western Blots.

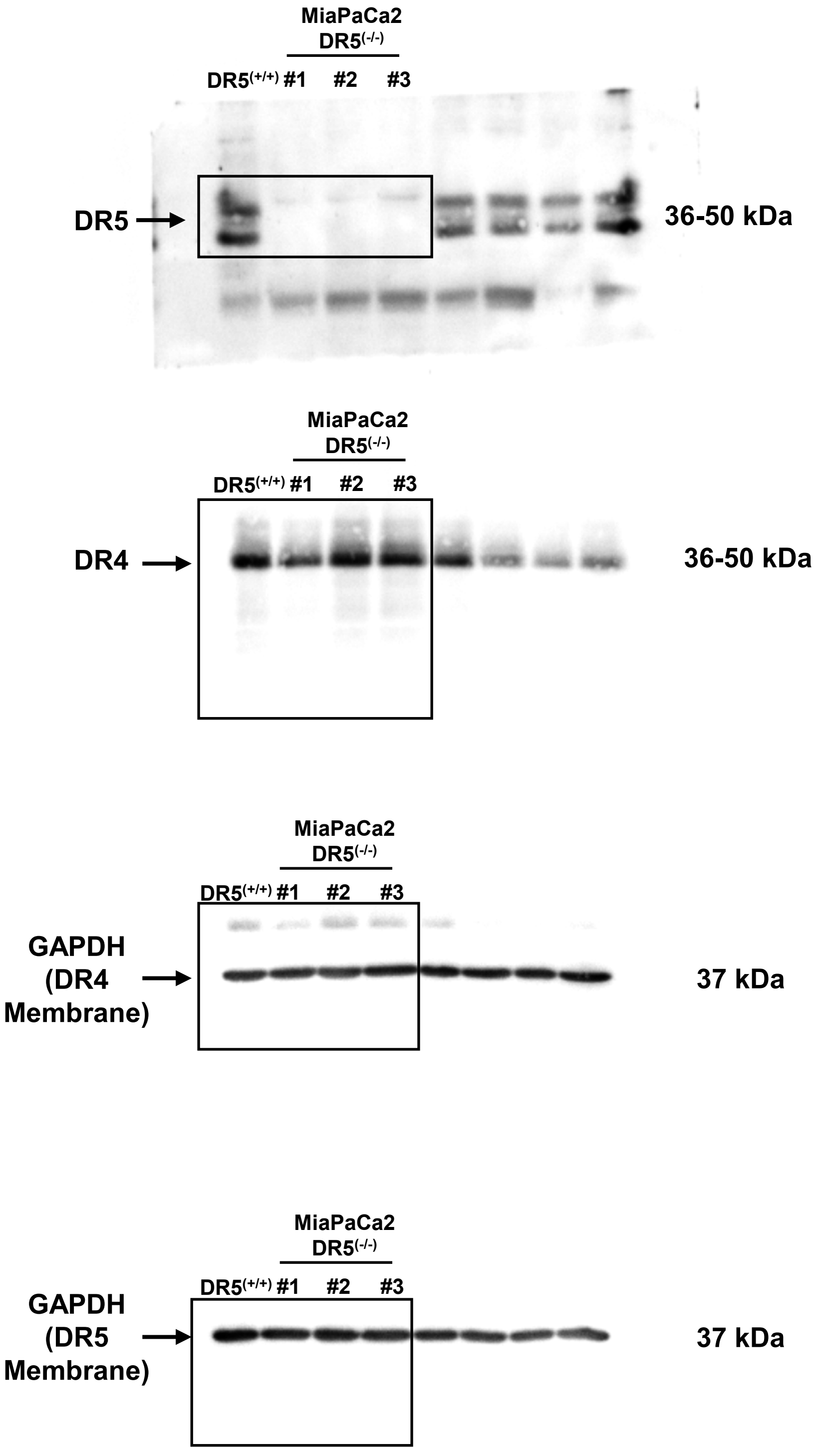

Figure 5E Raw Western Blots. Figure 5E membranes were cut into strips and imaged.

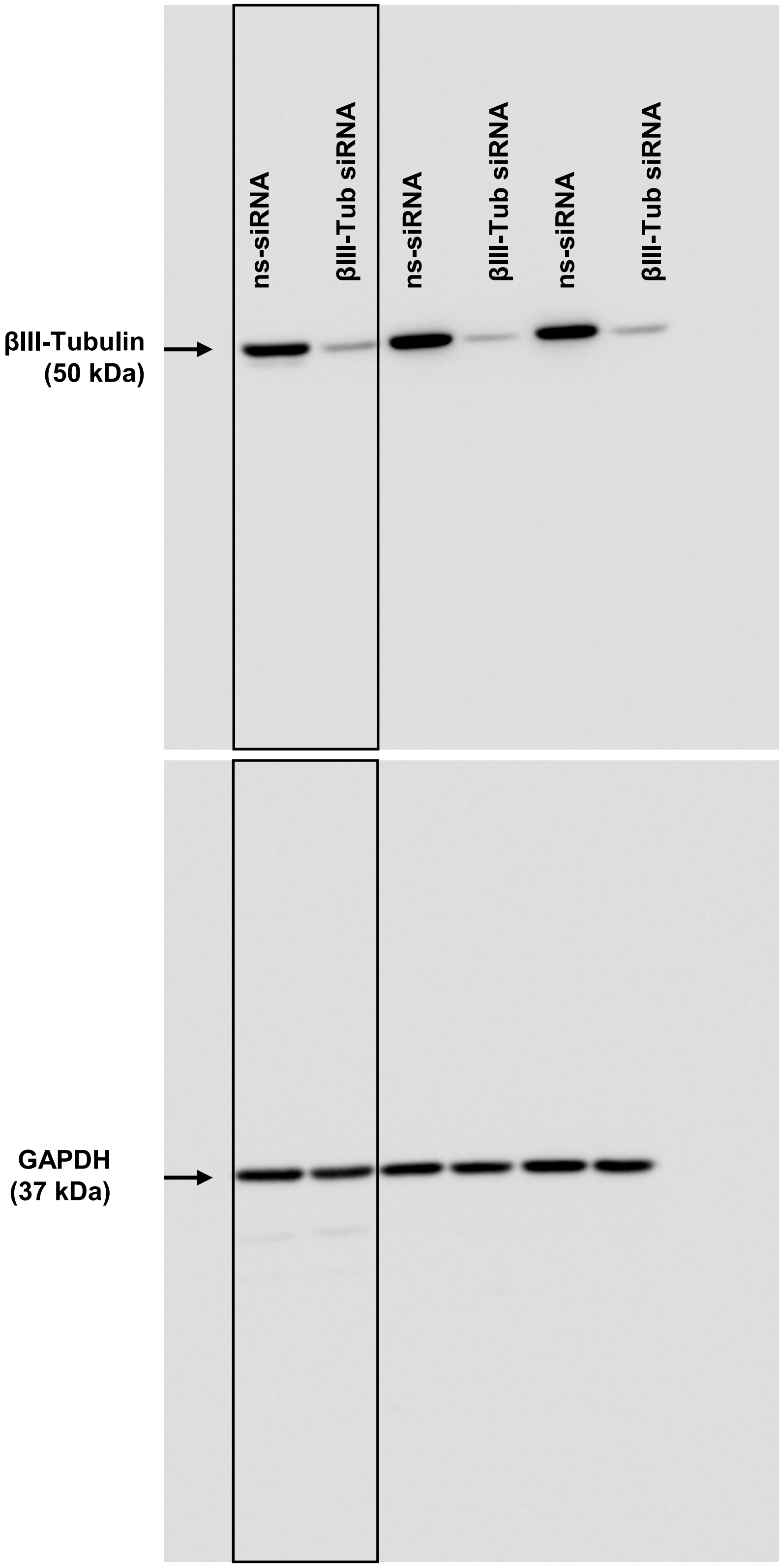

Supplementary Figure 1C Raw Western Blots.

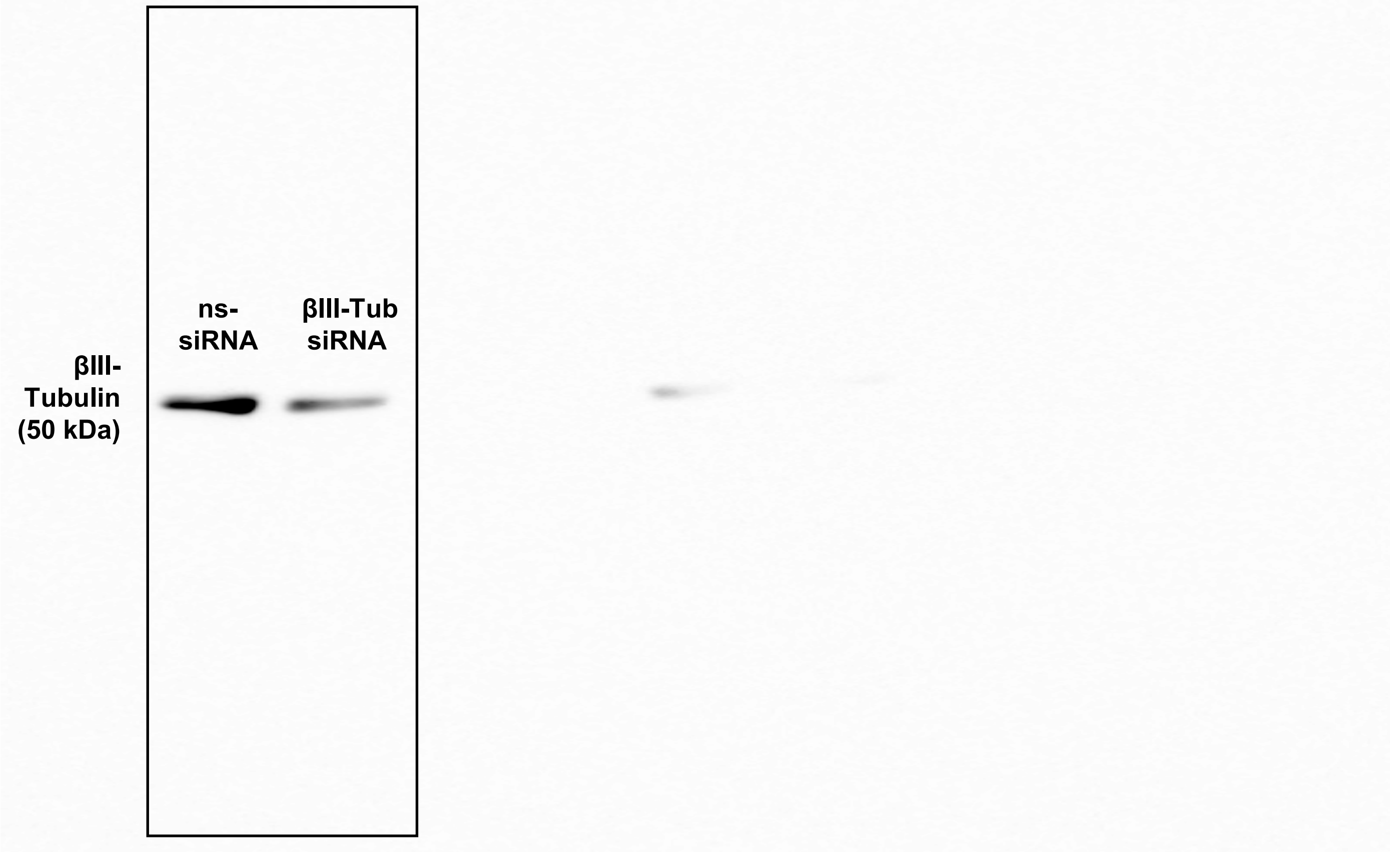

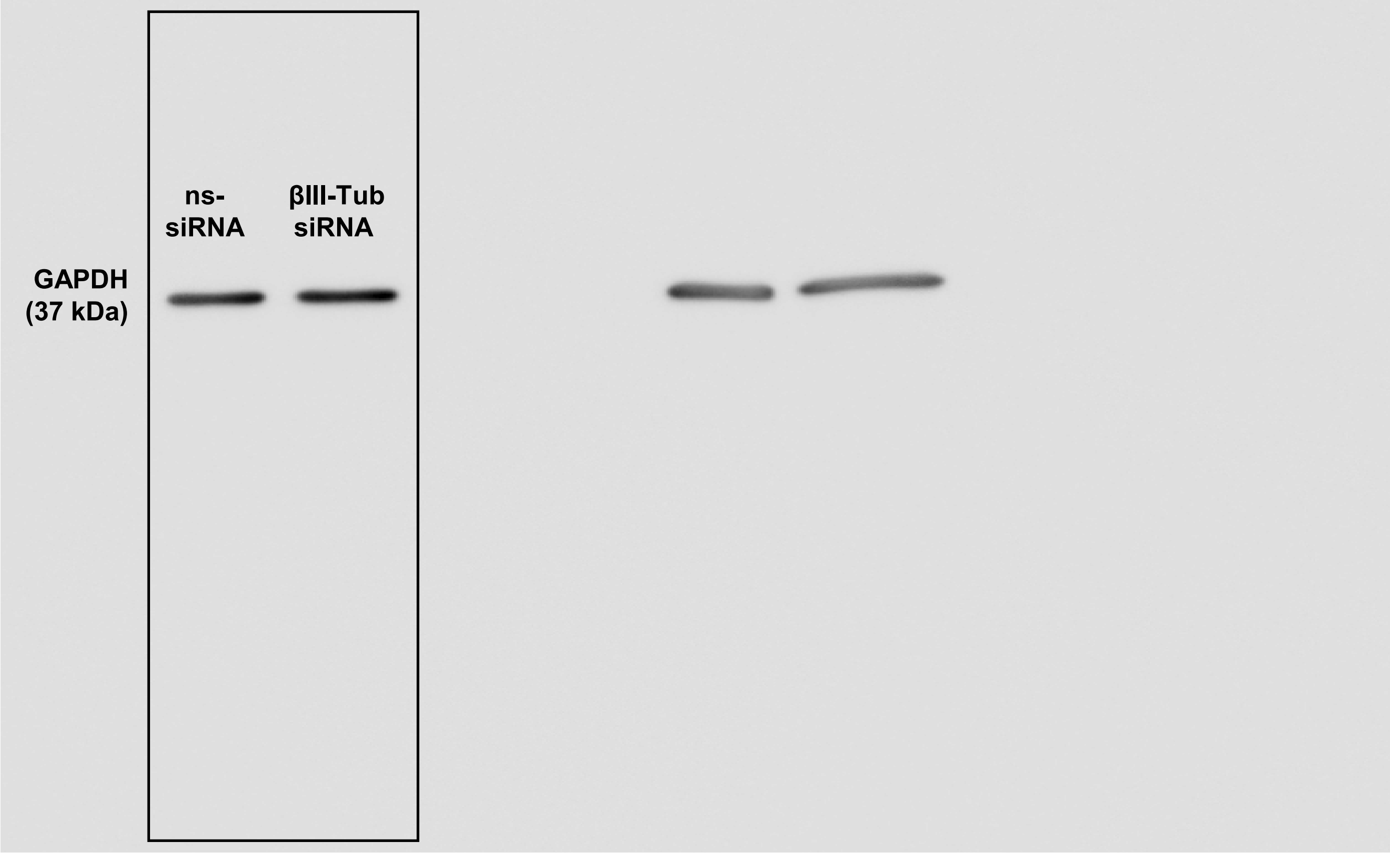

Supplementary Figure 1D Raw Western Blots.

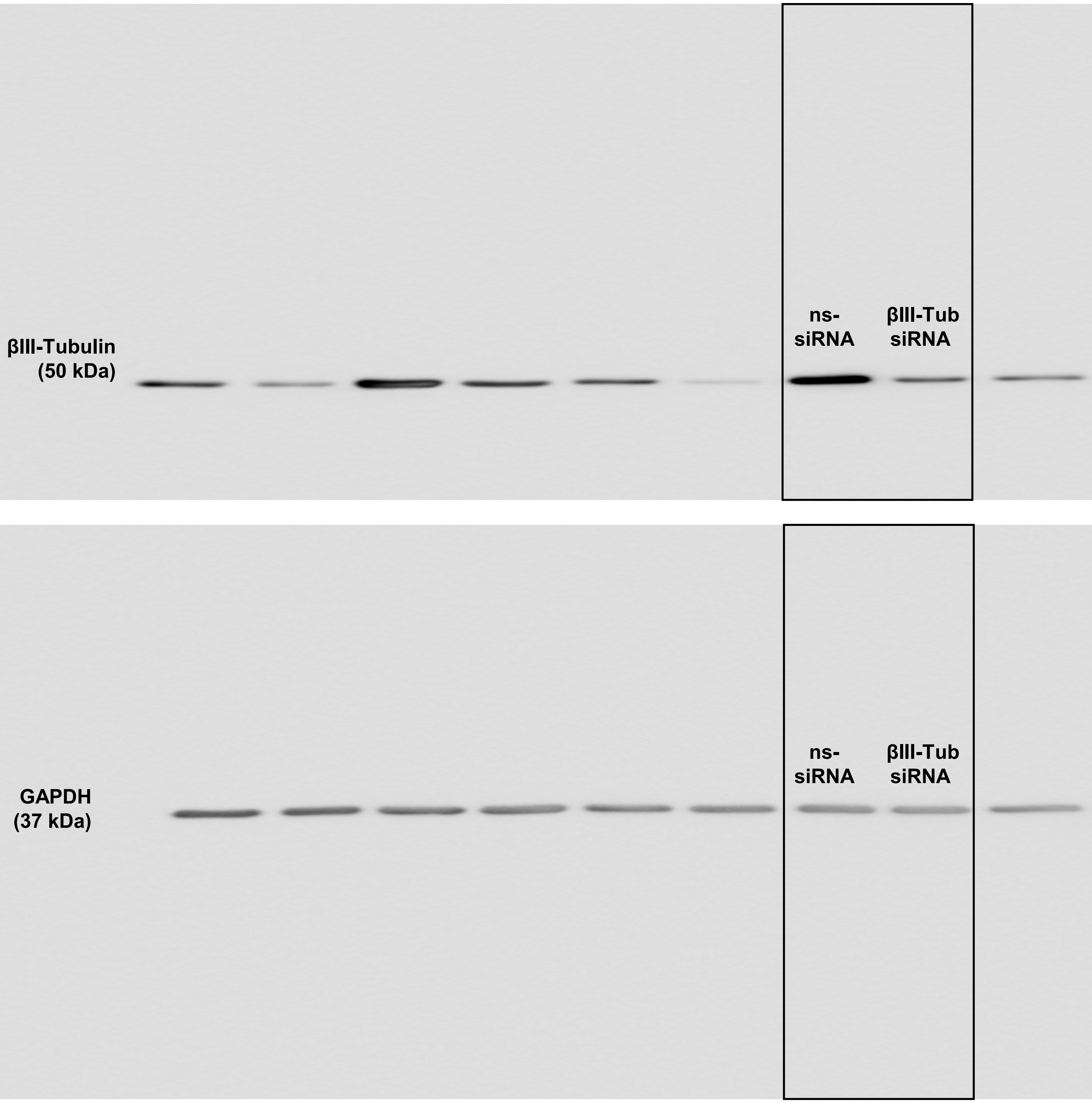

Supplementary Figure 1E Raw Western Blots.

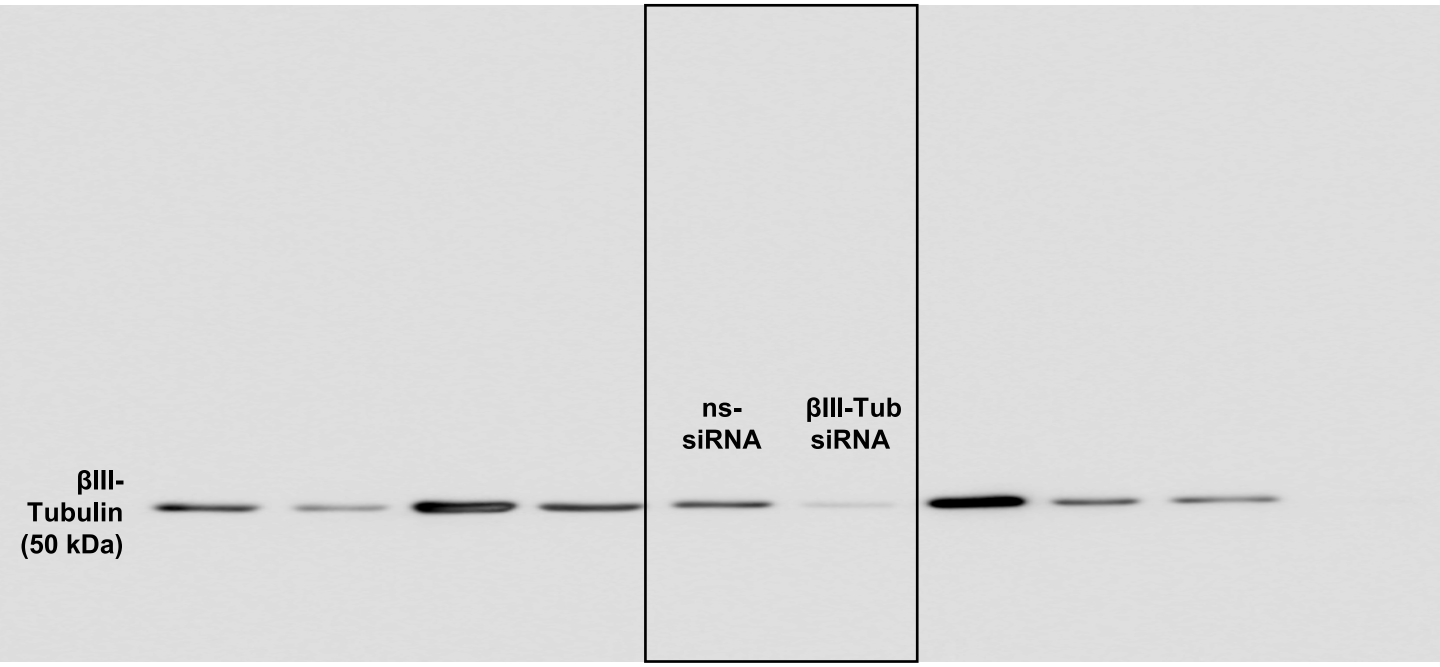

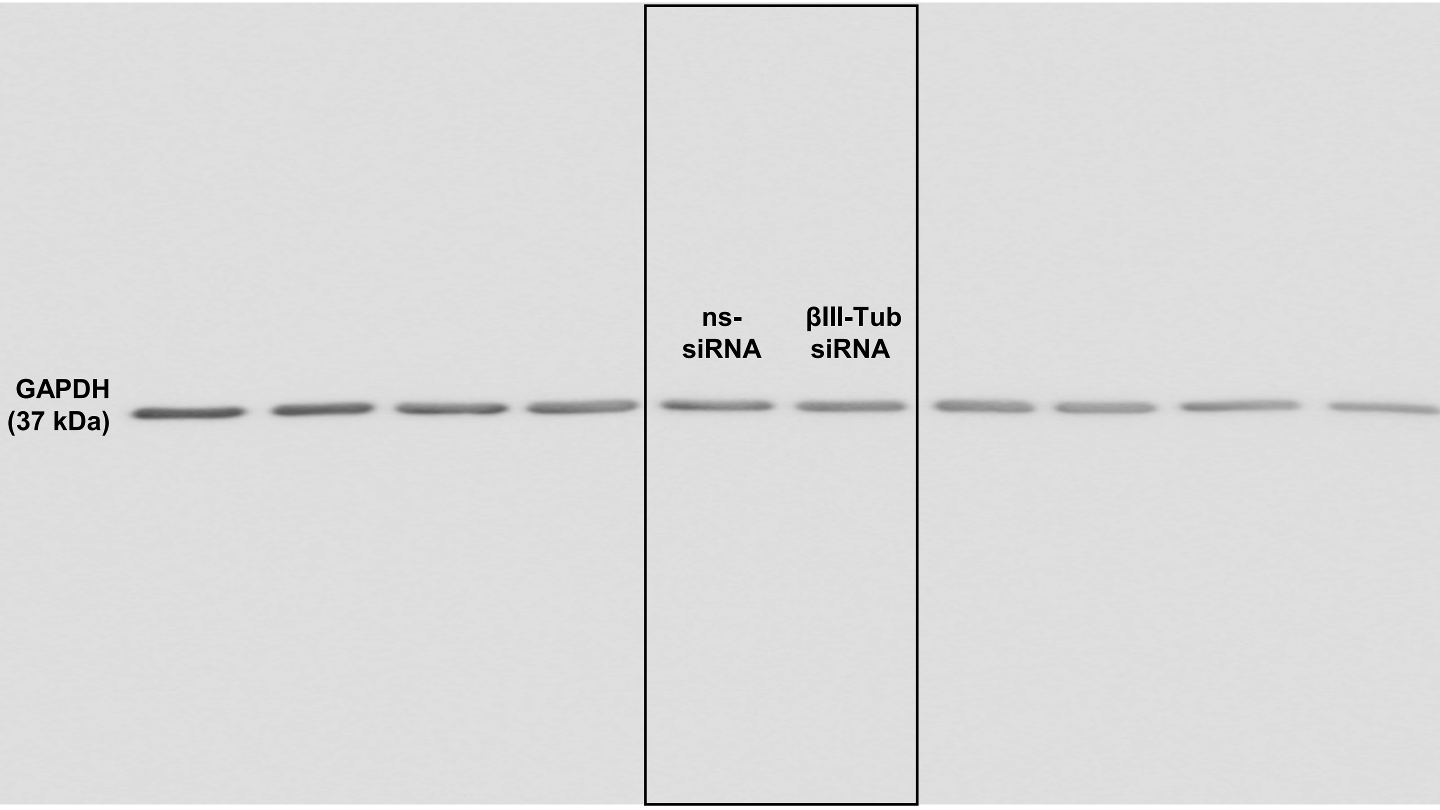

Supplementary Figure 1F Raw Western Blots.

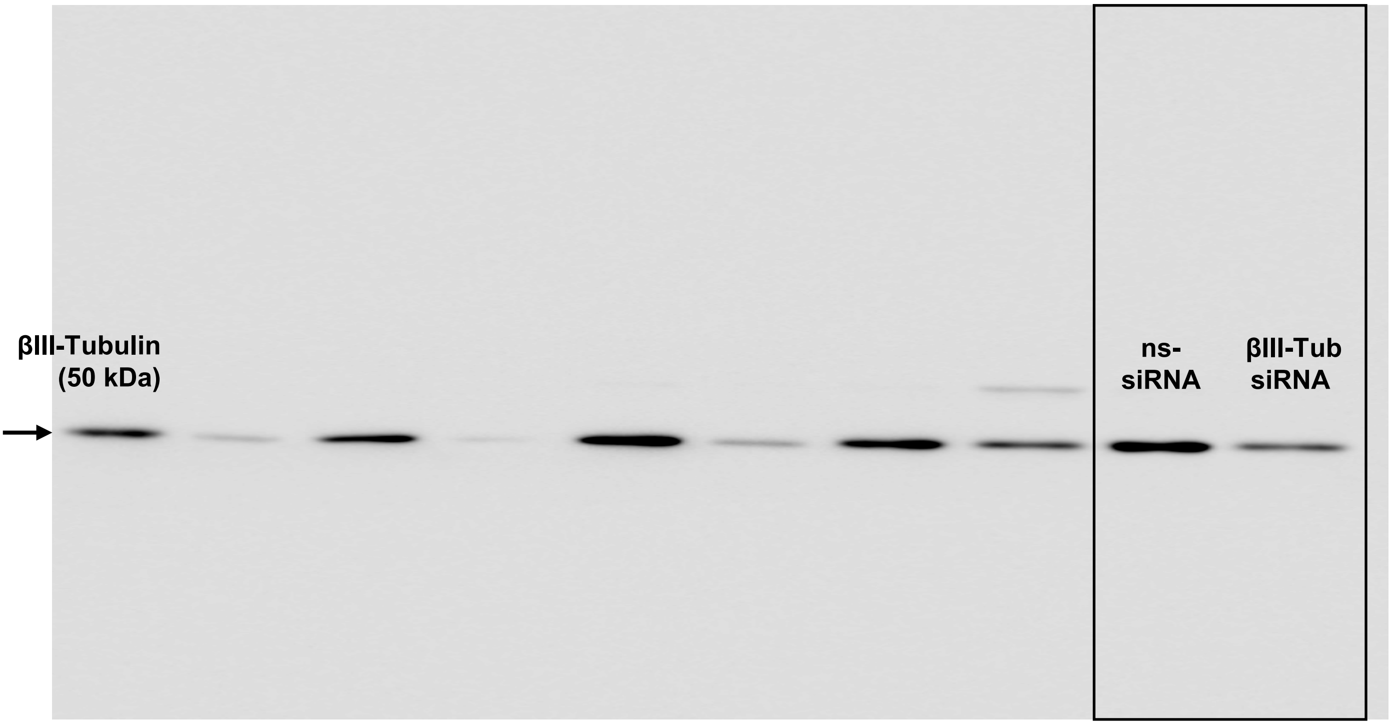

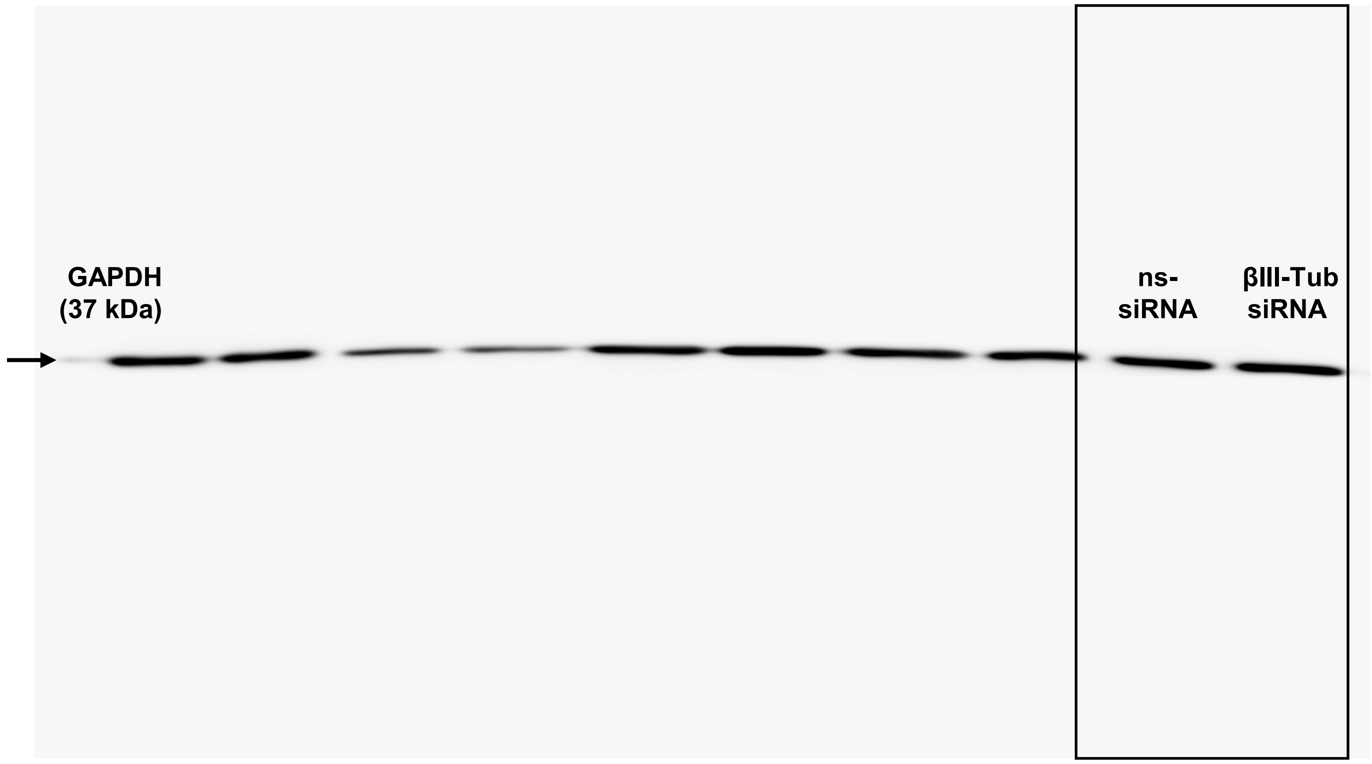

Supplementary Figure 1G Raw Western Blots.

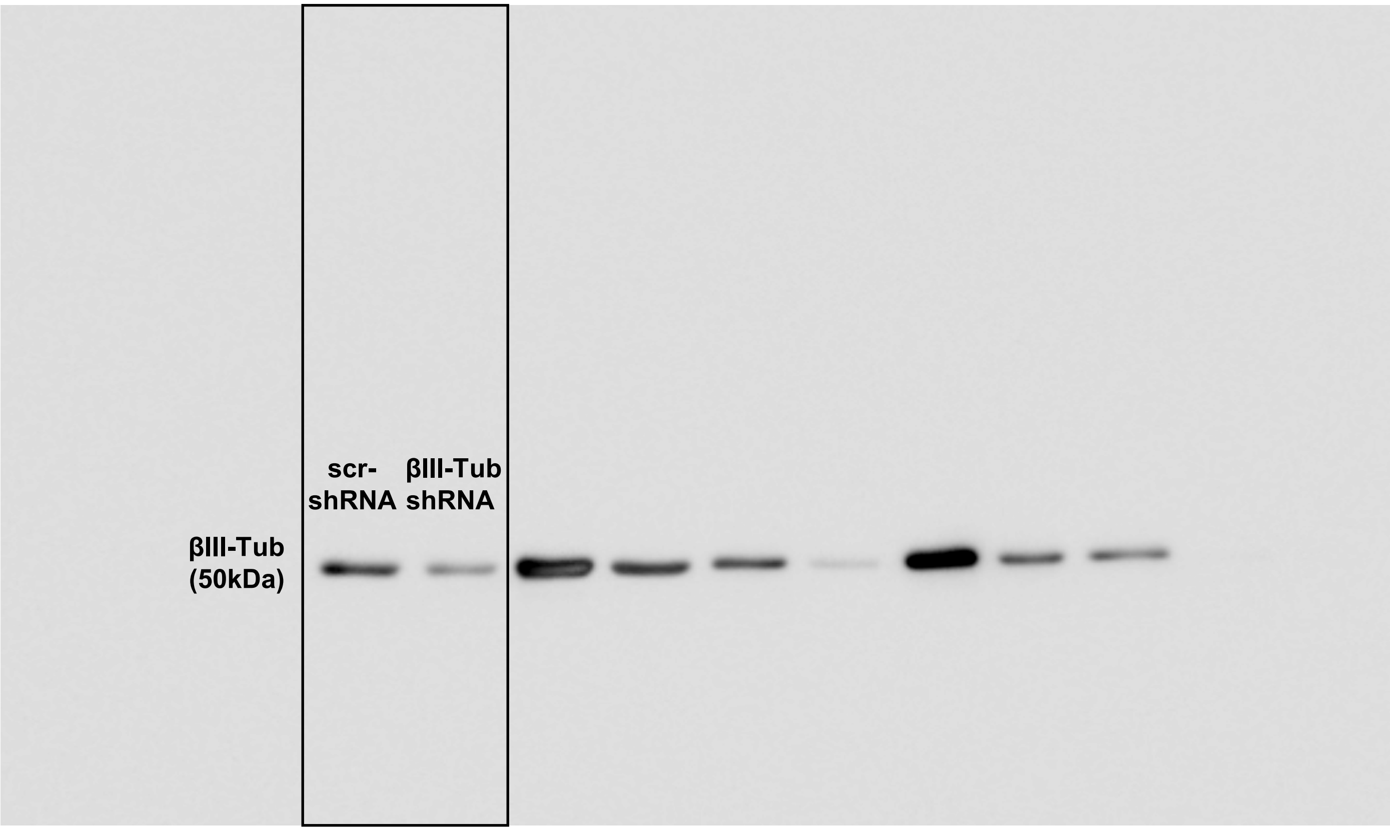

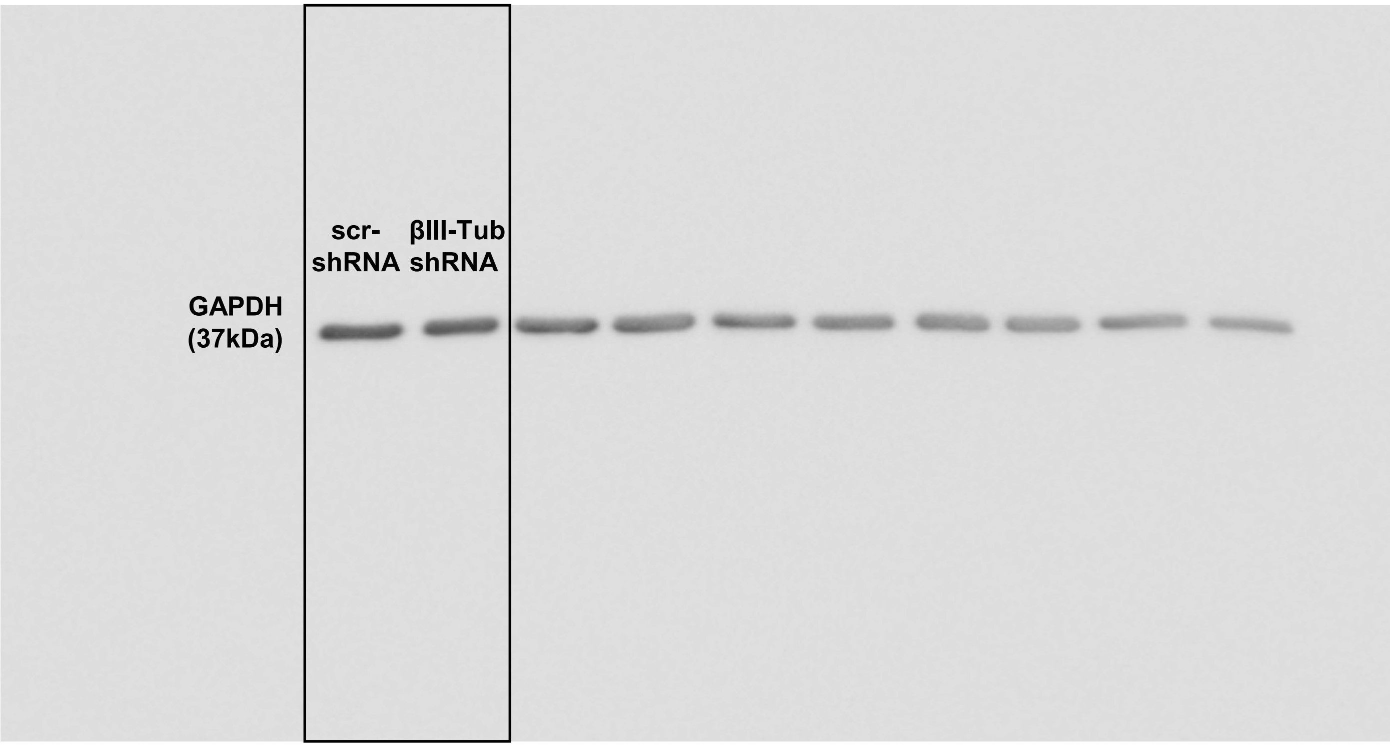

Supplementary Figure 1H Raw Western Blots.

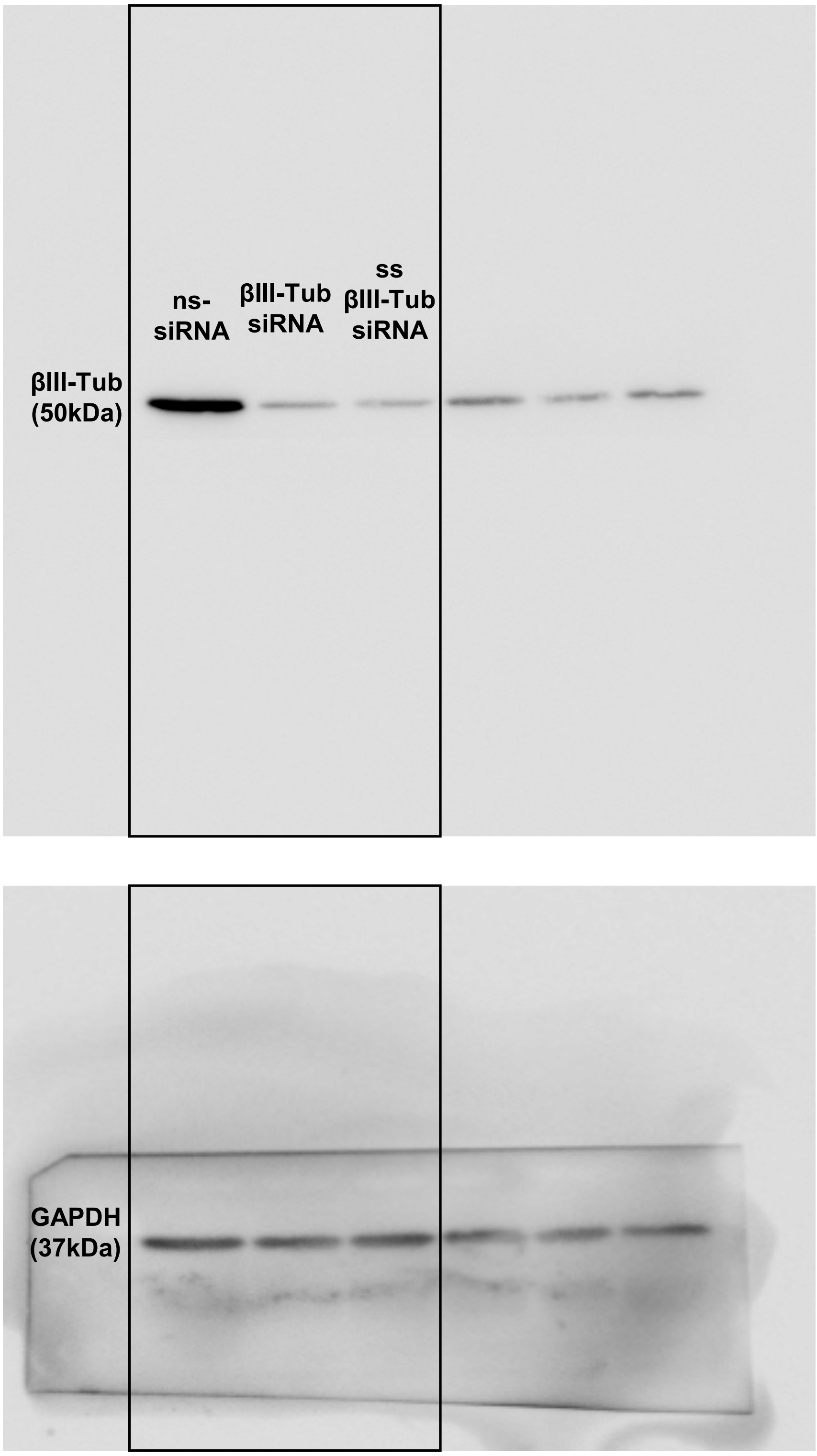

Supplementary Figure 1I Raw Western Blots.

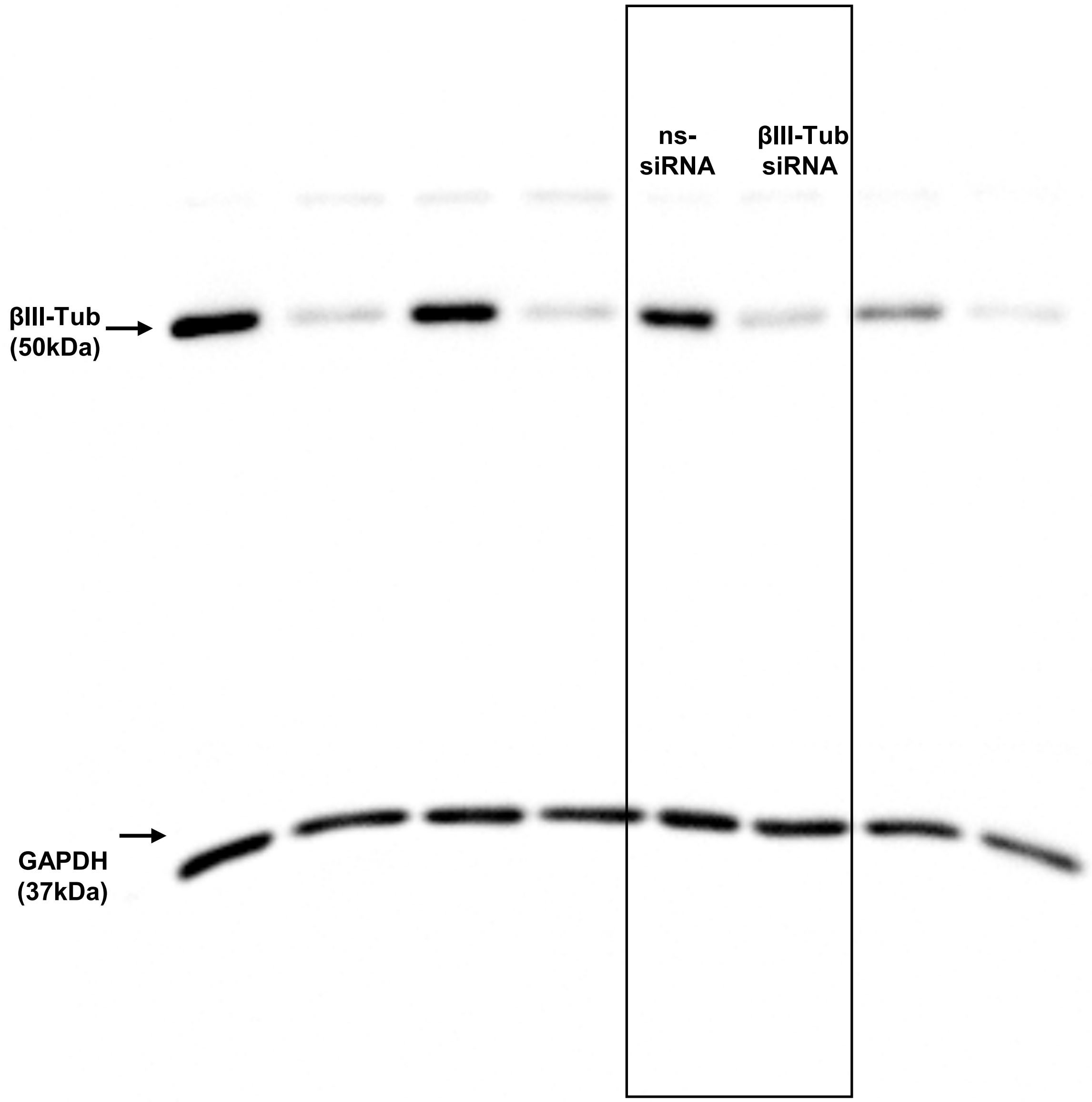

Supplementary Figure 1J Raw Western Blots.

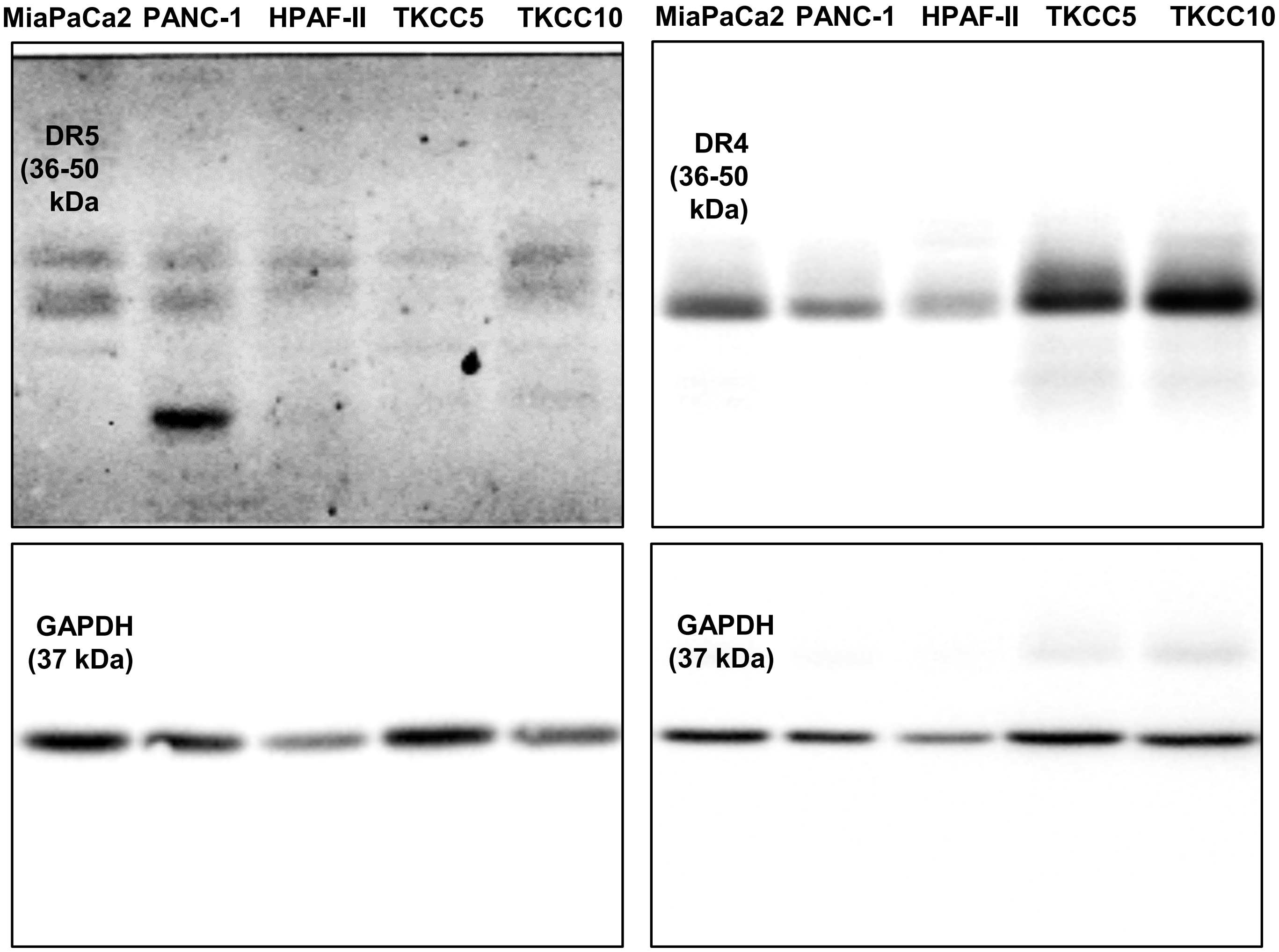

Supplementary Figure 2A Raw Western Blots.

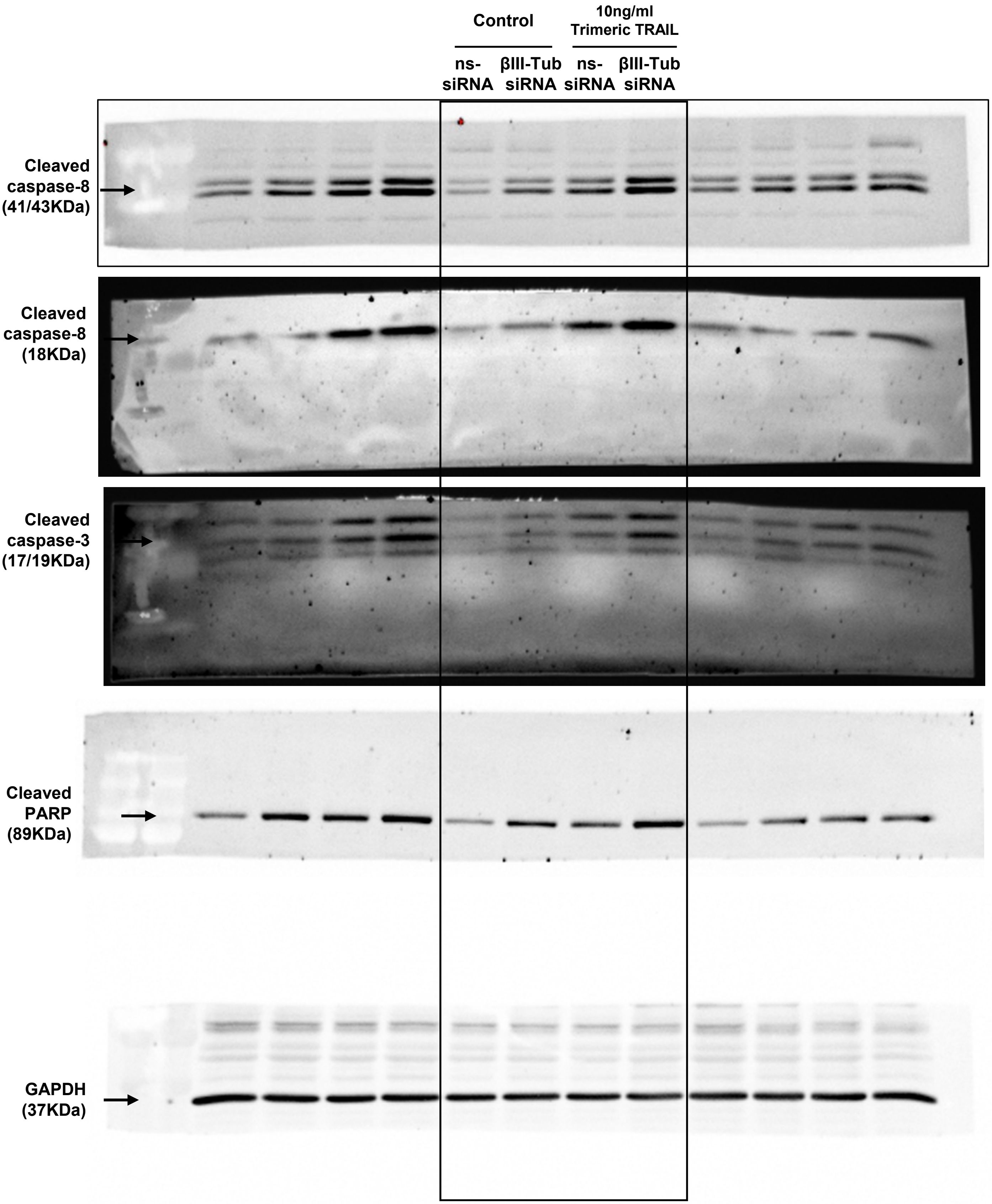

Supplementary Figure 2C Raw Western Blots. Figure 2C membrane was cut into strips and imaged. 17-19kDa strip was re-probed for cleaved caspase-3.

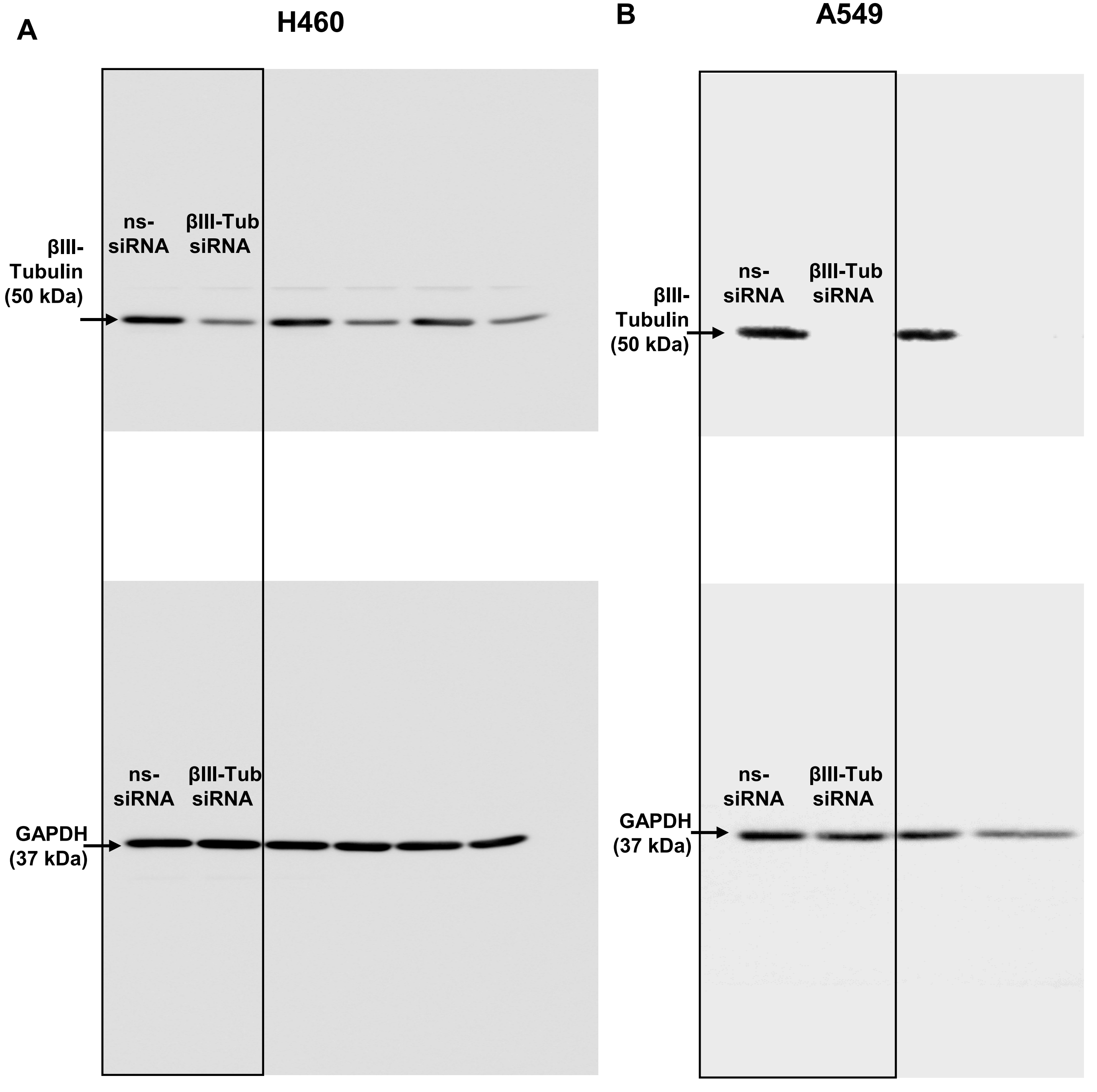

Supplementary Figure 3A-B Raw Western Blots.

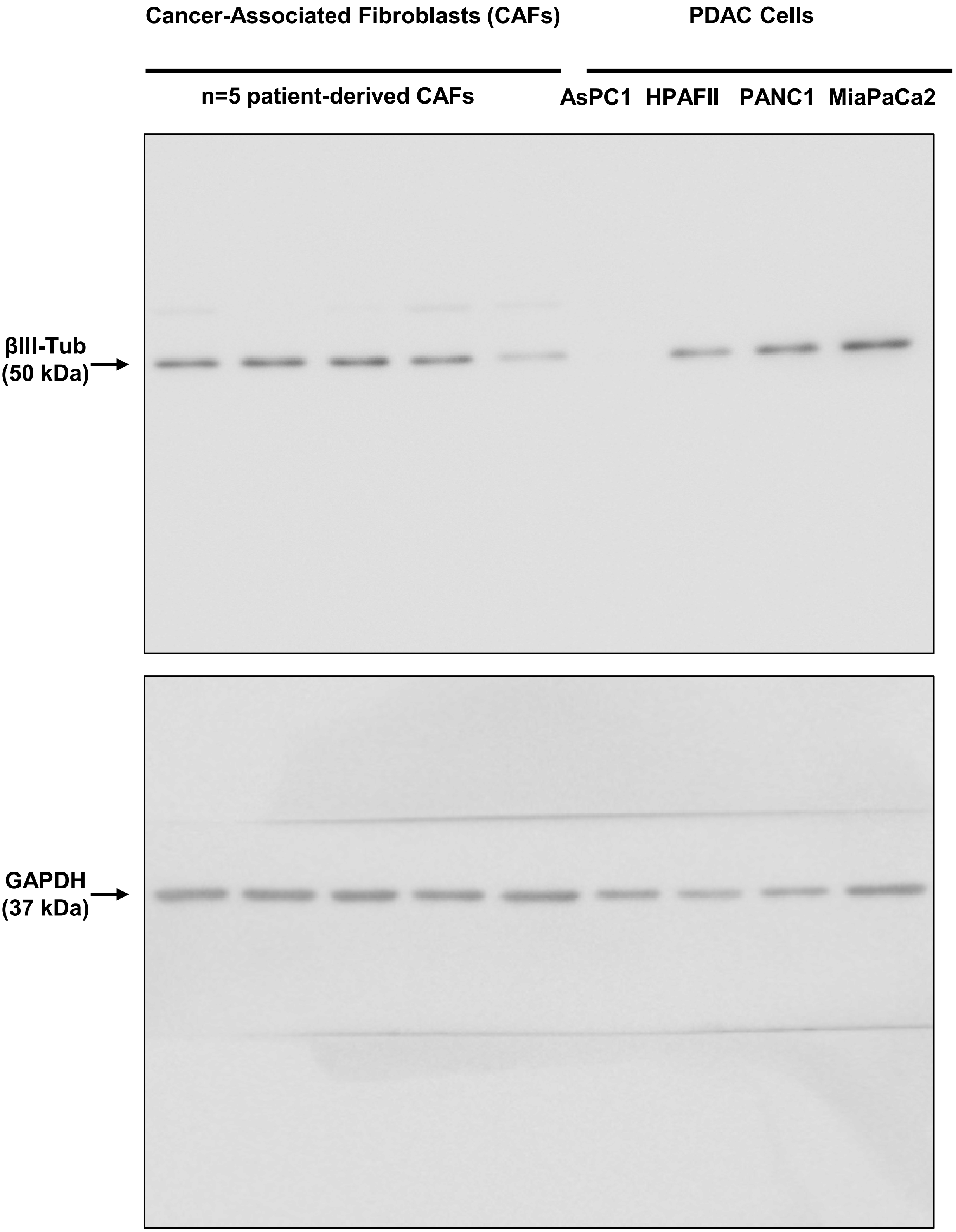

Supplementary Figure 4A Raw Western Blots.

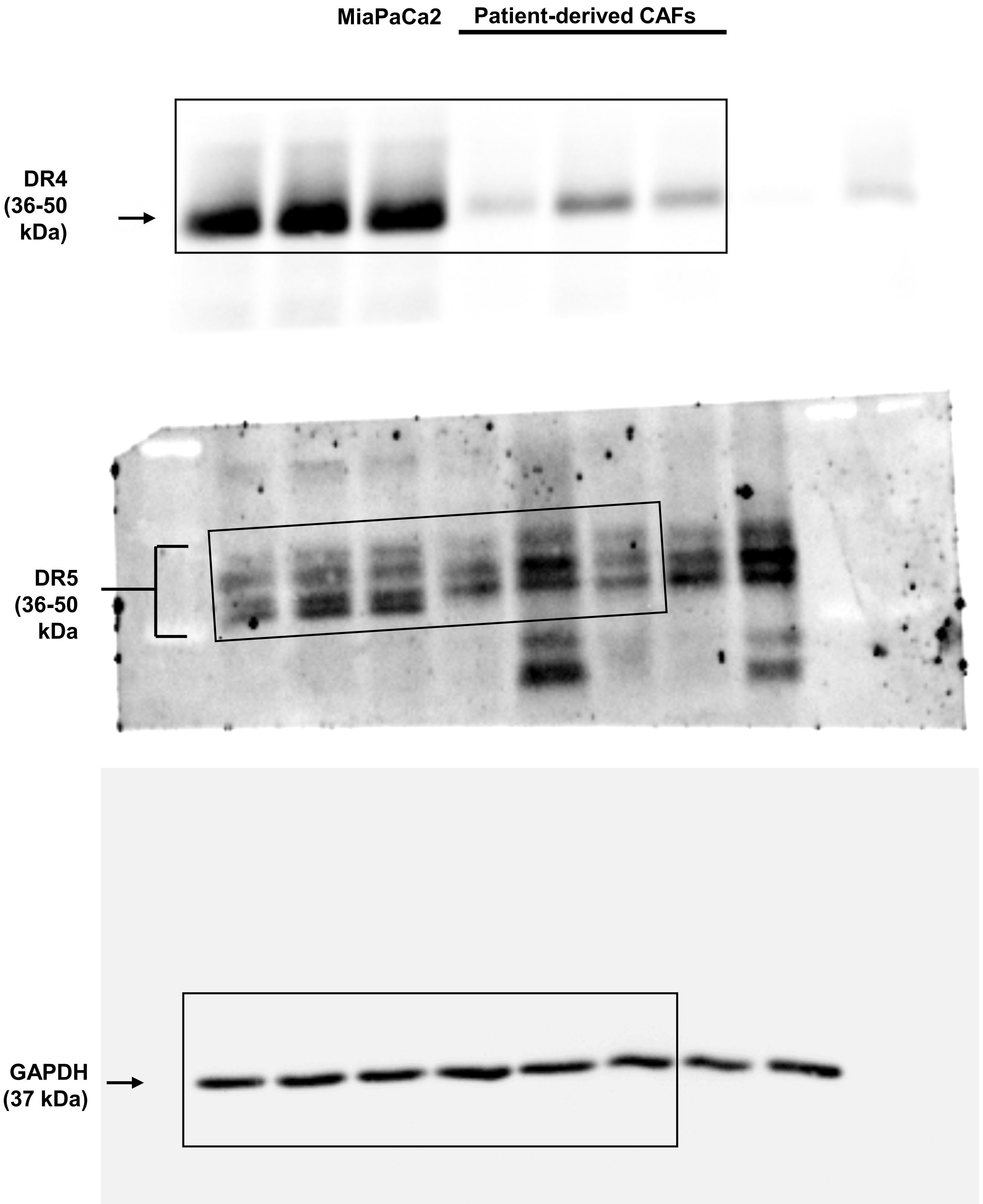

Supplementary Figure 4D Raw Western Blots.

MiaPaCa2

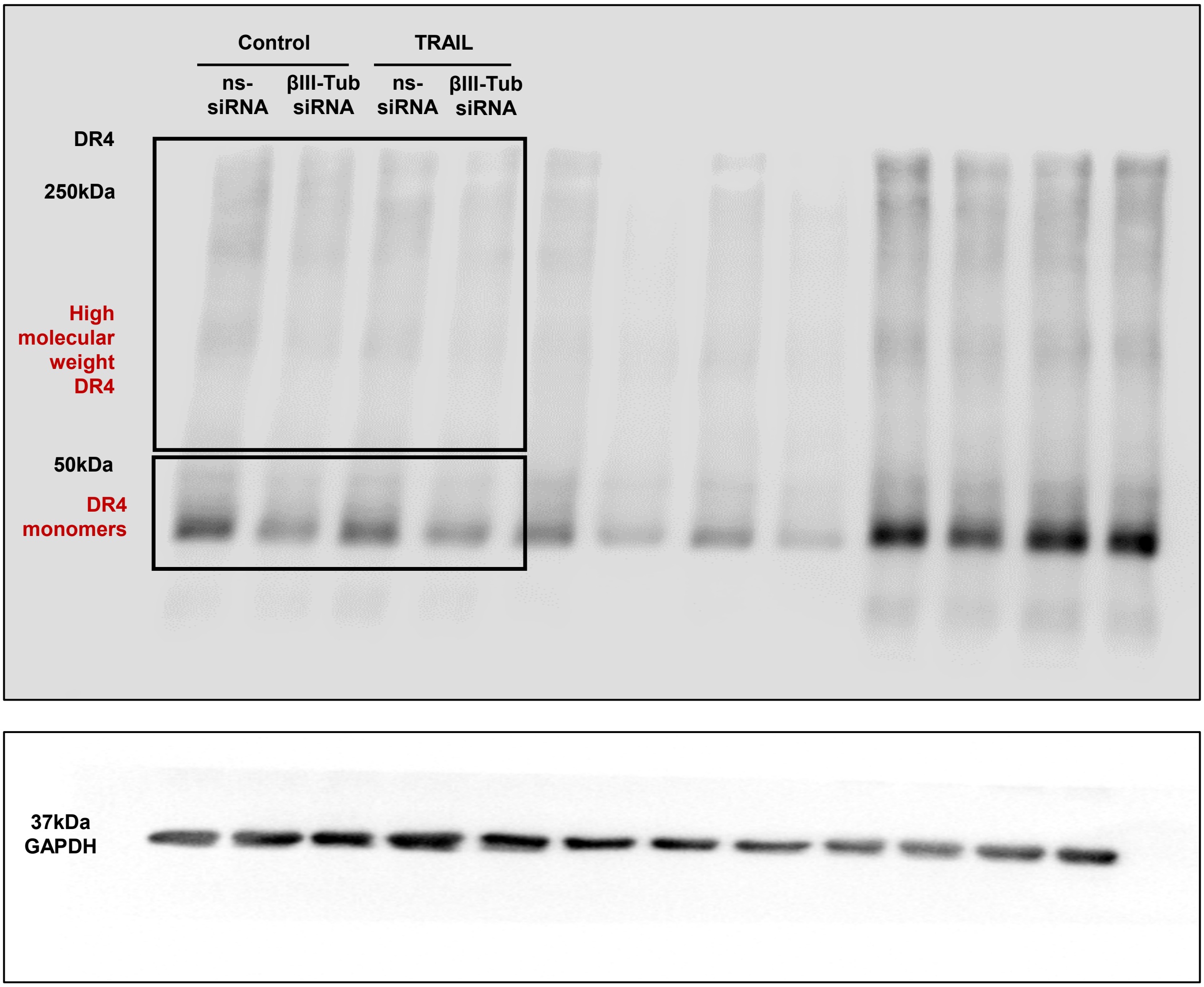

Supplementary Figure 6B.
